# Identification of Putative Markers of Non-infectious Bud Failure in Almond [*Prunus dulcis* (Mill.) D.A. Webb] Through Genome Wide DNA Methylation Profiling and Gene Expression Analysis in an Almond × Peach Hybrid Population

**DOI:** 10.3389/fpls.2022.804145

**Published:** 2022-02-14

**Authors:** Katherine M. D’Amico-Willman, Gina M. Sideli, Brian J. Allen, Elizabeth S. Anderson, Thomas M. Gradziel, Jonathan Fresnedo-Ramírez

**Affiliations:** ^1^Center for Applied Plant Sciences, The Ohio State University, Wooster, OH, United States; ^2^Department of Plant Sciences, University of California, Davis, Davis, CA, United States; ^3^Department of Horticulture and Crop Science, The Ohio State University, Wooster, OH, United States

**Keywords:** DNA methylation, non-infectious bud failure, differentially methylated regions (DMR), *Prunus* interspecific hybrids, quantitative RT-PCR, methylome

## Abstract

Almond [*Prunus dulcis* (Mill.) D.A. Webb] is an economically important nut crop susceptible to the genetic disorder, Non-infectious Bud Failure (NBF). Despite the severity of exhibition in several prominent almond cultivars, no causal mechanism has been identified underlying NBF development. The disorder is hypothesized to be associated with differential DNA methylation patterns based on patterns of inheritance (i.e., via sexual reproduction and clonal propagation) and previous work profiling methylation in affected trees. Peach (*Prunus persica* L. Batsch) is a closely related species that readily hybridizes with almond; however, peach is not known to exhibit NBF. A cross between an NBF-exhibiting ‘Carmel’ cultivar and early flowering peach (‘40A17’) produced an F_1_ where ∼50% of progeny showed signs of NBF, including canopy die-back, erratic branching patterns (known as “crazy-top”), and rough bark. In this study, whole-genome DNA methylation profiles were generated for three F_1_ progenies exhibiting NBF and three progenies considered NBF-free. Subsequent alignment to both the almond and peach reference genomes showed an increase in genome-wide methylation levels in NBF hybrids in CG and CHG contexts compared to no-NBF hybrids when aligned to the almond genome but no difference in methylation levels when aligned to the peach genome. Significantly differentially methylated regions (DMRs) were identified by comparing methylation levels across the genome between NBF- and no-NBF hybrids in each methylation context. In total, 115,635 DMRs were identified based on alignment to the almond reference genome, and 126,800 DMRs were identified based on alignment to the peach reference genome. Nearby genes were identified as associated with the 39 most significant DMRs occurring either in the almond or peach alignments alone or occurring in both the almond and peach alignments. These DMR-associated genes include several uncharacterized proteins and transposable elements. Quantitative PCR was also performed to analyze the gene expression patterns of these identified gene targets to determine patterns of differential expression associated with differential DNA methylation. These DMR-associated genes, particularly those showing corresponding patterns of differential gene expression, represent key targets for almond breeding for future cultivars and mitigating the effects of NBF-exhibition in currently affected cultivars.

## Introduction

Non-infectious Bud Failure (NBF) is a disorder in almond trees first observed in the early 1900s (Wilson and Stout, 1944; Micke, 1996) but formally described in 1944 (Wilson and Stout, 1944). While NBF was initially hypothesized to be a viral disease, no study to date has identified an infectious agent (thus fulfilling Koch’s postulates), so NBF is currently thought to be a genetic disorder (Wilson and Schein, 1956; Fenton et al., 1988). In trees exhibiting NBF, vegetative buds formed in the previous season die instead of emerging in the spring (Wilson and Schein, 1956). Affected buds tend to occur on shoots formed in warmer temperatures later in the growing season (i.e., sylleptic shoots), while buds on proleptic and epicormic shoots seem to be less affected (Micke, 1996). Typically, terminal buds exhibit greater mortality than basal buds in NBF shoots, so when the unaffected buds break dormancy, lateral shoots grow to create a “witch’s broom” like branching pattern that has lent NBF its colloquial name: “crazy top” (Wilson and Schein, 1956; Kester, 1969; Hellali et al., 1978). Subterminal buds develop on lateral branching but then fail to complete growth and do not swell. In addition to these signs, rough bark has also been described in affected trees (Wilson and Schein, 1956; Kester and Jones, 1970), appearing within the first 5 years of the tree’s life. Since vegetative buds emerge after flowering, nut set is not affected in the first year of NBF-exhibition, but growth and floral bud development are limited in subsequent years, causing yield losses of up to 50% (Kester, 1969; Micke, 1996).

The exhibition of NBF became a serious threat to the almond industry beginning in the 1940s when the promising and popular cultivar ‘Jordanolo’ began to exhibit the disorder and was subsequently abandoned (Wilson, 1950, 1952; Wilson and Schein, 1956). This pattern later repeated in the cultivar ‘Carmel,’ which was widely used until the early 2000s when widespread NBF-exhibition among clones led to its rapid abandonment (Kester et al., 2004; California Department of Food and Agriculture, 2020; D’Amico Willman, 2021). This disorder has also been observed in almond growing regions outside the United States, including in Australia, Spain, and Iran and is known to affect several cultivars of almond, including ‘Nonpareil,’ and ‘Mission.’ Due to its unknown etiology, irreversibility, and the current lack of screening methods, NBF is a threat to both current cultivars as well as any future almond breeding and production efforts (Kester and Jones, 1970).

Clones of different cultivars vary in the level and severity of NBF-exhibition (Kester, 1969; Micke, 1996). While NBF is common in cultivars such as ‘Carmel,’ the effects of NBF have become increasingly noticeable in “Nonpareil.” However, NBF has never been observed in peach, a closely related *Prunus* species that can readily hybridize with almond (Socias et al., 2017). Due to this discrepancy, test crosses have been performed to produce almond × peach interspecific hybrids to elucidate the genetic and epigenetic factors contributing to NBF-development (Kester, 1978). Almond × peach hybrids display an almond-like appearance and show 1:1 segregation of the NBF-phenotype in the F_1_ hybrid generation, suggesting that a single locus in the almond parent may contribute to the NBF-phenotype (Kester, 1978). A field scoring method for rating the degree of NBF disorder has been adopted according to the percent of affected scaffolding in almond trees: (1) possible dieback caused by NBF seen in one scaffold, (2) two scaffolds are affected, (3) three scaffolds are affected, (4) affected by yield, and (5) the tree appears to be dead with little to no leaves. Typically, NBF symptoms will occur on the southwest side of the tree first.

Due to these characteristics, NBF has long been hypothesized to be a genetic disorder (Wilson and Schein, 1956); however, more recent work suggests that NBF-exhibition may also involve epigenetic factors such as differential DNA methylation (Fresnedo-Ramírez et al., 2017). Results from this previous study support the association between NBF-exhibition and increased clonal age in a cultivar-specific manner and demonstrate an association between methylation and NBF-status in almond (Fresnedo-Ramírez et al., 2017). However, the method employed in this study (methylation-sensitive amplified fragment length polymorphism) provides only a genome-level profile of DNA methylation, which does not allow for a more precise analysis of locus-specific methylation status. Thus, the next step is to pinpoint where in the genome differential methylation occurs and identify potentially affected genes or gene features.

In this study, we examined genes and postulate gene features potentially involved in NBF development in almond using a selection of the ‘Carmel’ almond × ‘40A-17’ peach population currently available at the Wolfskill Experimental Orchards (University of California, Davis, Winters, CA, United States). ‘Carmel’ has been shown to exhibit NBF symptoms, and ‘40A-17’ is an early flowering peach. In this population, there is a 1:1 segregation for NBF-exhibition, enabling the selection of trees that exhibit NBF symptoms and trees free of NBF symptoms. DNA-methylation profiling was performed in this group of contrasting peach × almond individuals showing “crazy top” and rough bark symptoms. Following methylation analysis, regions of differential methylation associated with NBF-exhibition were identified, along with genes associated with those regions. Expression patterns of this set of genes were examined to assess the possible impact of differential methylation on gene activity. Through this work, we are attempting to identify genes that can be used as possible targets to assist in the selection and breeding of future almond cultivars without the threat of NBF-exhibition.

## Materials and Methods

### Sample Collection

Non-infectious Bud Failure was identified visually in selections produced from a cross of an NBF-positive almond cultivar ‘Carmel’ × ‘40A-17’ peach made in 2012 and again in 2014. Visual indications of NBF include tree markings that exhibit a rough appearance in the cork cambium as well as empty spurs and bare branches that turn at angles, lending the disorder the name “crazy top.” Leaves were selected for sampling and profiling as previous work in almond has demonstrated an association between NBF and DNA methylation using leaf tissue (Fresnedo-Ramírez et al., 2017). Additionally, work in apple has shown that DNA methylation profiling in leaves is representative of whole-plant level DNA methylation at a given time, providing support that DNA methylation profiles generated from almond leaves will be informative (Daccord et al., 2017). For trees with NBF symptoms (designated as NBF hybrids hereafter), leaves were collected in spring 2019 and 2021 from three areas in the canopy where there was no fruit. For trees without NBF symptoms (designated as no-NBF hybrids hereafter), leaves were collected from three different regions of the tree. Leaves collected in 2019 were stored on ice immediately following collection in the field and subsequently shipped to the Ohio Agricultural Research and Development Center (Wooster, OH, United States) for sample processing and DNA methylation analysis. Leaves collected in 2021 were placed in sterile pre-chilled 5 mL tubes with tweezers following sterile technique and placed in dry ice. Leaf samples were then frozen in liquid nitrogen and stored at −80°C until RNA extraction at UC Davis (Davis, CA, United States).

### DNA Extraction and Enzymatic Methyl-Seq Library Preparation and Sequencing

To isolate genomic DNA from leaves, samples were first ground to a fine powder in liquid nitrogen using a mortar and pestle. Approximately 150 mg of powder was used as input to the SILEX isolation method outlined in Vilanova et al. (2020). Following DNA isolation, quality and concentration were assessed by fluorometry using a Qubit™ and Qubit™ HS DNA kit (Thermo Fisher Scientific, Waltham, MA, United States). Whole-genome enzymatic methyl-seq libraries were prepared using the NEBNext^®^ Enzymatic Methyl-seq kit (New England Biolabs^®^ Inc., Ipswich, MA, United States) following the protocol for standard insert libraries (370–420 base pairs). Each sample was prepared using 100 ng input DNA in 48 μL TE buffer (1 mM Tris–HCl; 0.1 mM EDTA; pH 8.0) with 1 μL spikes of both the CpG unmethylated Lambda and CpG methylated pUC19 control DNA provided in the kit. The samples were sonicated using a Covaris^®^ S220 focused-ultrasonicator in microTUBE AFA Fiber Pre-Slit Snap-Cap 6 mm × 16 mm tubes (Covaris^®^, Woburn, MA, United States) with the following program parameters: peak incident power (W) = 140; duty factor = 10%; cycles per burst = 200; treatment time (s) = 80.

Following library preparation, library concentration and quality were assessed by fluorometry using a Qubit™ 4 and Qubit™ 1X dsDNA HS Assay Kit (Thermo Fisher Scientific) and by electrophoresis using a TapeStation (Agilent, Santa Clara, CA, United States). Library concentration was further quantified by qPCR using the NEBNext^®^ Library Quant Kit for Illumina^®^ (New England Biolabs^®^ Inc., Ipswich, MA, United States). Libraries were equimolarly pooled, and the pool was cleaned using an equal volume of NEBNext^®^ Sample Purification Beads (New England Biolabs^®^ Inc.). The library pool was eluted in 25 μL TE buffer (1 mM Tris–HCl; 0.1 mM EDTA; pH 8.0), and concentration and quality were assessed by fluorometry and electrophoresis as above. The library pool was sequenced on two lanes of the Illumina^®^ HiSeq 4000 platform to generate 150-bp paired-end reads.

### RNA Extraction and cDNA Synthesis

Total RNA was extracted from pooled leaves from trees in Table 1 using the Thermo Fisher Scientific PureLink™ Plant RNA Reagent (Thermo Fisher Scientific). Briefly, leaf tissue was ground under liquid nitrogen and insoluble PVP-40 with a mortar and pestle that had been pre-sterilized and treated with 10% bleach, ethanol, and RNaseZap (Thermo Fisher Scientific). The material was then weighed, and 100 mg was transferred to a tube containing 500 μl of 4°C PureLink reagent and a 3 mm steel bead. Samples were disrupted using a tissue lyser (Qiagen, Valencia, CA, United States) and separated using a centrifuge. The supernatant was mixed with 100 μl of 5 M NaCl and phase separation was performed using 300 μl of chloroform: isoamyl alcohol (24:1) followed by isopropanol precipitation and resuspension of the pellet in 30 μl of RNase-free water. Total RNA concentration was determined with a Qubit™ 4.0 and the Qubit™ RNA BR assay kit (Thermo Fisher Scientific), and purity was quantified with a NanoDrop™ 8000 spectrophotometer (Thermo Fisher Scientific). High-quality RNA at 100 ng/μl was used for cDNA synthesis using a High-Capacity cDNA Reverse Transcriptase Kit (Applied Biosystems, MA, United States) according to the manufacturer’s instructions.

**TABLE 1:** Identification of hybrids for RNA sampling including bud failure status and corresponding phenotype as well as year planted.

Genotype	Orchard ID	Bud failure status	Phenotype	Rough bark	Year planted
Carmel	Carmel	BF (+) control	Crazy top, major terminal dieback	0	1987
Okinawa	Okinawa	BF (−) control	No branching dieback	0	2013
NoBF2	13,5-244	BF (−)	No branching dieback	0	2013
NoBF3	15,1-142	BF (−)	No branching dieback	0	2015
NoBF4	15,1-174	BF (−)	No branching dieback	0	2015
NoBF5	15,2-457	BF (−)	No branching dieback	0	2015
NoBF6	15,2-465	BF (−)	No branching dieback	0	2015
BF7	13,5-245	BF (+)	rat tailing^1^, bare lateral branching	1	2013
BF8	13,5-246	BF (+)	3 affected scaffolds, rat tailing	1	2013
BF9	13,5-265	BF (+)	3 affected scaffolds, rat tailing	1	2013
BF10	15,1-177	BF (+)	2 affected scaffolds, rat tailing	1	2015
BF11	15,2-390	BF (+)	2 affected scaffolds, rat tailing	1	2015
BF12	15,2-410	BF (+)	2 affected scaffolds, rat tailing	1	2015
BF13	15,2-431	BF (+)	2 affected scaffolds, rat tailing	1	2015

*^1^Rat tailing lateral branch that has dieback and new growth at tips.*

### Processing and Alignment of Enzymatic Methyl-Seq Libraries

Methyl-Seq read quality was initially assessed using FastQC v. 0.11.7 (Andrews, 2010), and reads were trimmed using TrimGalore v. 0.6.6 and Cutadapt v. 2.10 with default parameters (Krueger, 2016). Forward read fastq and reverse read fastq files from the two HiSeq 4000 lanes were combined for each library to produce single fastq files for both read one and read two. Reads were aligned to the ‘Nonpareil’ v. 2.0 almond reference genome (NCBI BioProject PRJNA769745 GenBank accession: JAJFAZ000000000) developed by our group, and separately to the double haploid ‘Lovell’ v. 2.0 peach reference genome (NCBI BioProject PRJNA31227), deduplicated, and methylation calls were generated using Bismark v. 0.22.3 (Krueger and Andrews, 2011) with default parameters in paired-end mode. These reference genomes were selected as ‘Nonpareil’ and ‘Lovell’ are the most closely related individuals to the parents (‘Carmel’ and ‘40A-17’) in the interspecific cross. To test conversion efficiency, reads were also aligned to both the Lambda and pUC19 nucleotide sequence fasta files provided by NEB^1^. All analyses were performed using the Ohio Supercomputer Center computing resources (Ohio Supercomputer Center, 1987).

### Weighted Whole-Genome Methylation and Differential Methylation Analysis

Using the methylation calls generated by Bismark, weighted whole-genome methylation levels were calculated for each methylation context [CG, CHG, and CHH (H = A, T, or C)]. Weighted genome-wide percent methylation values were calculated for each individual by taking the total number of methylated reads at each cytosine and dividing this by the total number of reads (summation of methylated plus unmethylated reads) at each cytosine. These values were used as input to R statistical software v. 4.0.2 (R Core Team, 2021) to perform beta regression using the package “betareg” v. 3.1-3 (Cribari-Neto and Zeileis, 2010). Pairwise comparison of least squared means was completed by the functions *emmeans()* and *cld()* from the R packages “emmeans” v. 1.5.2-1 and “multcomp” v. 1.4-14 with an alpha = 0.05 and Sidak adjustment (Hothorn et al., 2008).

Coverage files for each methylation context produced by Bismark were prepared for input into the R package DSS (Dispersion Shrinkage for Sequencing Data) v. 2.38.0 (Wu et al., 2013; Feng et al., 2014; Park and Wu, 2016). The functions *DMLtest()* and *callDMR()* were used with a significance p.threshold set to 0.0001 to identify differentially methylated regions (DMRs) through pairwise comparisons between the NBF- and no-NBF hybrids. Comparisons were made relative to the no-NBF hybrids in each DMR test.

Following identification of DMRs in each methylation context, DMRs were further characterized based on the directionality of differential methylation. Hypermethylated DMRs are those that show increased methylation in the NBF hybrids compared to the no-NBF hybrids, and hypomethylated DMRs are those that show decreased methylation in the NBF hybrids compared to the no-NBF hybrids.

To visualize enrichment of DMRs across the eight chromosomes in the almond and peach reference genomes, circos plots were generated with one track depicting each DMR classified as either hyper- or hypomethylated and two additional tracks depicting DMR enrichment across the genome. To create the circos plots, the R package circlize v. 0.41.2 (Gu et al., 2014) was used along with the bed files for all hyper- and hypomethylated DMRs in each methylation context. The command *circos.genomicDensity()* was used to create the two tracks representing enrichment of hyper- and hypomethylated DMRs on each chromosome where the taller the peak, the higher the number of DMRs occurring in the specific region (Gu et al., 2014). Finally, percent overlap of DMRs with genome features was performed using the *annotateWithFeatures()* command in the R package methylKit v. 1.16.1 (Akalin et al., 2012).

### Quantitative RT-PCR

Complementary DNA templates were diluted in a series and run on a QuantStudio 3 (Thermo Fisher Scientific) with PowerTrack SYBR green (Thermo Fisher Scientific) and a known reference gene, *RPII* (Tong et al., 2009), to determine the optimal concentration range to use for qRT-PCR analysis. Amplification efficiency was evaluated using a standard curve where the cDNA concentration of 10 ng/μl was determined to have the most consistent Cq values. The 10 μl reaction contained 1 μl cDNA template, 5 μl SYBR PowerTrack master mix, 0.25 μl forward and 0.25 μl reverse primers, and 3.5 μl water. The conditions for qRT-PCR were as follows: 95°C for 120 s, followed by 40 cycles at 95°C for 5 s, and 55°C for 30 s, and for the melt curve, 95°C for 15 s, and 55°C for 60 s, followed by 95°C for 15 s. An *RPII* inter-run calibrator was used on each plate.

The “areaStat” statistic produced in DSS was used to select the most significantly differentially methylated regions (DMRs) in each context and alignment (peach or almond). Genes nearby or overlapping these select DMRs were used for primer design. Several DMRs identified using the peach and almond reference genomes were associated with the same gene in the corresponding genome. A subset of DMRs was only identified in the peach alignment or almond alignment, and in some cases, the gene associated with a DMR was not present in the other genome. In cases where the same gene was associated with DMRs from each genome, primers were designed based on both the peach and almond alignments; however, in cases where the gene associated with the DMR was only present in one genome, only one primer pair was designed. For primer pairs with multiple products amplified, the annealing temperature was adjusted to 53°C. A total of 48 primer pairs (Supplementary Table 1) were tested across the eight Non-infectious Bud Failure samples and seven control (no Non-infectious Bud Failure) samples.

### Gene Expression Analysis

Thermocloud Data Connect Platform (Thermo Fisher Scientific) was used to analyze the raw cycle of quantification values (Cq values) contained in experiment (.eds) files. Specificity was checked using a melt curve in the Design and Analysis app (DA2) to ensure there were no extraneous products. Some samples which displayed curves with multiple peaks, indicating more than one target amplification, were removed. Further analysis was carried out utilizing the Relative Quantification (RQ) app where global normalization was performed (Mestdagh et al., 2009) as RPII was not expressed in all samples. For relative quantification measurements, ‘Okinawa,’ a peach with no bud failure symptoms, was set as the reference sample. An auto threshold was set for determining normalized Cq values. *RPII* was set as the inter-run calibrator. Student’s *t-*test was performed for the samples in the biogroups (NBF and no-NBF). R-statistical software packages “ggplot2” and “reshape” were used for the correlation matrix heatmap design.

### Analysis of Predictability of Non-infectious Bud Failure

To estimate the predictability of NBF symptoms based on gene expression in the hybrids and considering the limited sample size, the Bootstrap Forest platform implemented in JMP Pro 15.2.1 (SAS Institute Inc, 2020) was used. Globally normalized Cq values were entered for each hybrid tested in the population (‘Carmel’ and ‘Okinawa’ were excluded, see Supplementary Table 3). Missingness was not considered informative, and there was no ordinal restriction in the order of the outcomes (NBF vs. no-NBF). In the test, 1,000 trees were considered in the forest, sampling 12 terms per split, bootstrapping a sample rate of 1, with a minimum of 10, and a maximum of 2,000 splits per tree with a minimum split size of 5. The seed number for the pseudo-random generator was set as 121221. From this first round of bootstrapped aggregation (i.e., bagging), each of the 49 genomic features (i.e., coding sequences) was ranked according to the *G*^2^ value, which is like a sum of squares estimator to measure goodness of fit for the case of nominal outcomes. Genomic features with nil prediction power were identified. Subsequently, genomic features found with any statistical predictive ability were further processed through the Bootstrap Forest, this time considering 10,000 trees in the forest to estimate importance indices as independent uniform inputs. Based on resampling of globally normalized Cq values, genomic features with higher predictive ability for NBF in the current sample were determined.

### Gene Annotation

Following expression analysis, genes were selected for further annotation based on the expression profiles in the NBF hybrids relative to the no-NBF hybrids. Both nucleotide and protein alignments were performed for those genes showing differential expression patterns associated with NBF-exhibition. Alignments were performed using the Geneious Prime v. 2021.2.2 software suite MAFFT alignment option. Alignments were visualized using mView (available at https://www.ebi.ac.uk/Tools/msa/mview/) (Madeira et al., 2019).

## Results

### Whole-Genome Methylation Analysis

Following enzymatic methyl-seq, raw reads were aligned to both the almond and peach reference genomes, and whole-genome methylation patterns were analyzed. Mapping efficiency to the peach reference genome ranged from 30.8 to 44%, while mapping efficiency to the almond reference genome ranged from 43.9 to 55.6% (Supplementary Table 2), similar to what has been observed in other perennial species using Bismark (Xu et al., 2017; Müller et al., 2020). To test for conversion efficiency (i.e., the rate at which unmethylated cytosines are converted to uracils), all reads were also aligned to the lambda genome, and methylated cytosines were called. Based on this alignment, conversion efficiency ranged from 99.1 to −99.9% for all libraries (Supplementary Table 2).

**FIGURE 1:**
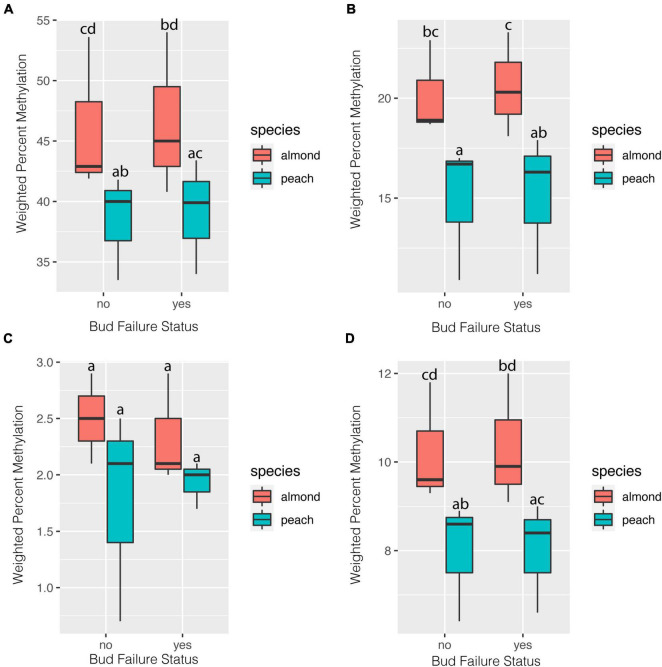
Weighted genome-wide percent methylation in NBF (*n* = 3) and no-NBF hybrids (*n* = 3) based on alignment to both the almond and peach reference genomes. Weight percent methylation is reported for each methylation context [CG **(A)**, CHG **(B)**, and CHH **(C)**] as well as total percent methylation **(D)**. Error bars represent 95% confidence intervals. Letters represent significance groupings for each methylation context and context determined by beta regression and least squares means with Sidak correction (alpha = 0.05).

Weighed whole-genome methylation levels were consistently higher in all methylation contexts and for total percent methylation when reads were aligned to the almond genome in comparison to the peach genome (Figures 1A–D). For both the CG and CHG context, methylation levels were higher in the NBF hybrids compared to the no-NBF hybrids using the almond genome alignment (Figures 1A,B). Weighted percent methylation was slightly lower in the NBF hybrids compared to the no-NBF hybrids for the CHH context using the almond genome alignment (Figure 1C). Levels of weighted percent methylation did not appear to differ based on NBF-status using the peach genome alignment.

### Identification of Differentially Methylated Regions Based on Bud Failure Status

Following genome-wide methylation analysis, regions of significant differential methylation between the NBF- and no-NBF hybrids were identified based on alignment to both the almond and peach reference genomes. Using the almond reference genome alignment, a total of 115,635 differentially methylated regions (DMRs) were identified. In contrast, 126,800 DMRs were identified using the peach reference genome alignment. For both the peach and almond alignments, the highest number of DMRs were identified in the CG context, followed by the CHG and CHH, respectively (Table 2).

**TABLE 2:** The number of differentially methylated regions (DMRs) in each methylation context [CG, CHG, CHH (H = A, T, C)] identified by comparing almond × peach hybrids with and without bud failure.

Species alignment	Methylation context	DMRs	Hypermethylated	Hypomethylated
Almond	CG	58,411	31,841	26,570
	CHG	29,324	8,727	20,597
	CHH	27,900	19,884	8,016
Peach	CG	61,404	10,883	50,521
	CHG	36,835	3,908	32,927
	CHH	28,561	4,457	17,588

*Alignments were performed with both the almond and peach reference genomes prior to calling for DMRs. Once identified, the DMRs were further characterized as either hyper- or hypomethylated when comparing the bud failure hybrids to the bud failure-free hybrids.*

The identified DMRs were further classified as either hyper- or hypomethylated based on the directionality of methylation difference. DMRs were classified as hypermethylated if there was a higher level of methylation in the NBF hybrids compared to the no-NBF hybrids, while DMRs were classified as hypomethylated if there was a lower level of methylation in the NBF hybrids compared to the no-NBF hybrids. In the CHG context, both the peach and almond alignments showed a greater number of hypomethylated DMRs compared to hypermethylated DMRs (Table 2). However, in the CG and CHH contexts, the peach and almond alignments showed opposite patterns of hyper- and hypomethylation, with the almond alignment showing a higher number of hypermethylated DMRs in both contexts, while the peach alignment shows a higher number of hypomethylated DMRs in both contexts (Table 2). These DMRs were further annotated by determining the percentage of DMRs that overlap genome features from each alignment using the almond and peach annotations (Table 3). The highest number of DMRs overlapping genes occurred in the CG context for both alignments, while DMRs overlapping 5′ and 3′ untranslated regions tended to occur in the CHG and CHH contexts (Table 3).

**TABLE 3:** Annotation of differentially methylated regions (DMRs) from the almond and peach alignments for each methylation context [CG, CHG, CHH (H = A, T, C)].

Species	Methylation context	Feature	Percent of DMRs overlapping feature
Peach	CG	Gene	52.47%
		Exon	43.48%
		5′ untranslated region	1.82%
		CDS	39.7%
		3′ untranslated region	6.88%
	CHG	Gene	26%
		Exon	19.76%
		5′ untranslated region	1.45%
		CDS	18.01%
		3′ untranslated region	1.62%
	CHH	Gene	25.34%
		Exon	17.80%
		5′ untranslated region	3.58%
		CDS	12.83%
		3′ untranslated region	3.05%
Almond	CG	Gene	44.78%
		5′ untranslated region	3.66%
		CDS	33.37%
		3′ untranslated region	9.66%
	CHG	Gene	12.14%
		5′ untranslated region	2.31%
		CDS	6.73%
		3′ untranslated region	2.68%
	CHH	Gene	22.11%
		5′ untranslated region	6.30%
		CDS	7.39%
		3′ untranslated region	6.05%

*Included are the percent of DMRs that overlap with each feature from the almond and peach genome annotation.*

**FIGURE 2:**
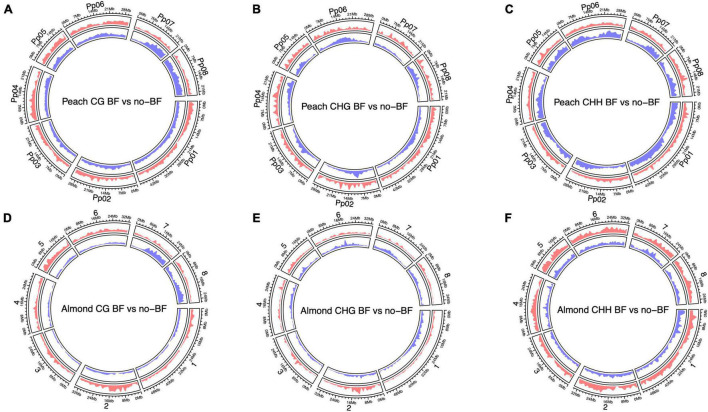
Circos plots displaying genome-wide differential methylation between NBF and no-NBF hybrids in each methylation context [CG, CHG, CHH (H = A, T, or C)] using both the almond and peach reference genomes. **(A–C)** Show differential methylation based on alignment to the peach genome for each methylation context. The red circle represents hypermethylation in the NBF hybrids compared to the no-NBF hybrids, and the blue circle represents hypomethylation in the NBF hybrids compared to the no-NBF hybrids. **(D–F)** Show differential methylation based on alignment to the almond genome with hypermethylation and hypomethylation displayed as described above. The *y*-axis represents genomic density defined as the fraction of a genomic window that is covered by a genomic region (in this case, a DMR).

Finally, enrichment of hyper- and hypomethylated DMRs across both the almond and peach genomes was visualized. Both the peach and almond genomes show enrichment of hypomethylated DMRs in the CG context on chromosomes seven and eight (Figure 2). The opposite pattern of methylation in the CHH context is evident in the visualization of DMRs, where enrichment of hypermethylated DMRs across the genome is observed in the almond alignment while there is enrichment of hypomethylated DMRs across the peach genome (Figure 2).

### Analysis of Expression Patterns of Genes Associated With Differentially Methylated Regions

Differentially methylated regions with the most significant differential methylation, based on the “areaStat” statistic produced in DSS in each context and alignment (peach or almond), were selected to search for nearby or overlapping genes. Several DMRs identified using the peach and almond reference genomes were associated with the same gene in the corresponding genome. A subset of DMRs was only identified in the peach alignment or almond alignment, and in some cases, the gene associated with a DMR was not present in the other species. Using the significance criteria (those genes associated with the most significantly differentially methylated regions), a total of 26 genes associated with 36 identified DMRs were selected for expression analysis (Supplementary Table 2). Expression patterns were tested in NBF- and no-NBF hybrids, as well as a ‘Carmel’ clone exhibiting NBF, and a peach cultivar ‘Okinawa’ known not to exhibit NBF.

### Statistical Analysis of Samples and Gene Targets

**FIGURE 3:**
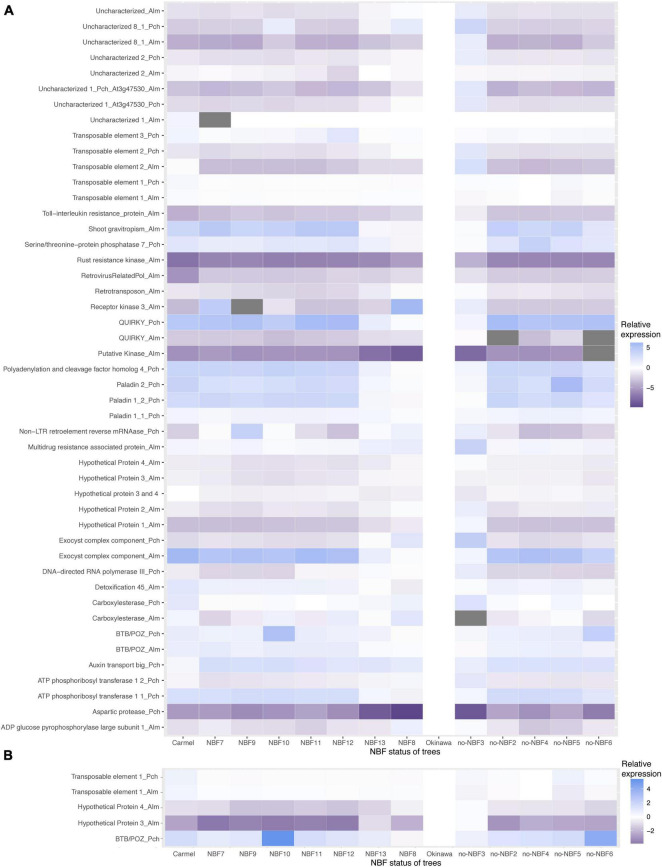
Heatmaps displaying normalized Cq values of DMR-associated genes for NBF and no-NBF trees. Samples were normalized to ‘Okinawa’ which was set to zero and displayed in white color. The scale shows increasing ΔΔCq values and lower relative gene expression with the color blue, and with lower ΔΔCq values and higher relative gene expression in purple. An underscore is used in the names to show whether the gene was from almond (_Alm) or peach (_Pch) alignments. **(A)** Displays all DMR-associated genes run at both 55 and 53°C annealing temperatures in the qPCR experiment. **(B)** Displays the top five predicted DMR-associated genes and their corresponding ΔΔCq values.

Patterns of gene expression varied among the genes tested using primers for either the peach allele or the almond allele of each particular gene (if present) (Figures 3A,B). The normalized Cq heatmap is arranged by gene expression of targets with a 55 and 53°C annealing temperatures (Figure 3A) and the top five gene targets (Figure 3B). Hypothetical proteins 2, 3, and 4 in almond showed higher gene expression in NBF trees compared to no-NBF trees. The average fold change differences between NBF and no-NBF for hypothetical protein 2 were −1.071 and −0.566, respectively. The average cycle differences between NBF and no-NBF trees for hypothetical protein 3 were −1.039 and −0.732. The average cycle differences between NBF and no-NBF trees for hypothetical protein 4 were −1.178 and −0.61. In the 53°C annealing temperature qPCR experiments, transposable element 1 peach showed high levels of expression in ‘Carmel,’ NBF7, NBF8, NBF9, NBF10, NBF11, NBF12, and NBF13, compared to the other trees tested (Figure 3A). The average fold change differences for NBF and no-NBF were −0.012 and 0.224, respectively.

In general, there is no pattern in which all the gene targets align with the classification of the hybrids according to NBF-exhibition. However, the general expression pattern showed that some hybrids showed an expression pattern resembling peach (Okinawa) and others showed an expression pattern similar to almond (Carmel). In order to statistically define coding gene targets whose expression may be associated with NBF symptoms, ensemble models were used averaging decision trees. Bootstrapping using normalized Cq values (Supplementary Table 2) was employed to rank these coding gene targets by their predictive ability (Table 4), which confirmed an association of expression and exhibition of NBF symptoms in gene targets such as *BTB/POZ domain containing protein FBL11*, and suggested an association of other gene targets such as *ADP glucose pyrophosphorylase large subunit 1*, *Rust resistance kinase Lr10*, and *ATP phosphorbosyl transferase 1* were not found at the top of main and total effect as importance indices for predictability for NBF, and their contribution was found to be negligible.

**TABLE 4:** Ranking of indices estimated in the assessment of variable importance for normalized Cq values of 48 gene targets considered in this study (RPII is not considered since it was used as a control).

Gene ID	Main effect	Total effect
Transposable element 1_Pch	0.288	0.304
Transposable element 1_Alm	0.286	0.302
Hypothetical Protein 4_Alm	0.203	0.218
BTB/POZ_Pch	0.020	0.031
Hypothetical Protein 3_Alm	0.013	0.023
Non-LTR retroelement reverse mRNAase_Pch	0.013	0.022
BTB/POZ_Alm	0.011	0.018
Rust resistance kinase_Alm	0.009	0.017
Uncharacterized 2_Alm	0.009	0.016
Serine/threonine-protein phosphatase 7_Pch	0.008	0.015
Hypothetical protein 2_Alm	0.007	0.012
Retrotransposon_Alm	0.006	0.010
Hypothetical protein 3 and 4	0.005	0.009
DNA-directed RNA polymerase III_Pch	0.003	0.006
Carboxylesterase_Alm	0.001	0.004
ATP phosphoribosyl transferase 1 2_Pch	0.002	0.003
Exocyst complex component_Alm	0.001	0.003
Paladin 2_Pch	0.001	0.003
Toll-interleukin resistance_protein_Alm	0.001	0.003
Uncharacterized 1_At3g47530_Pch	0.001	0.003
Polyadenylation and cleavage factor homolog 4_Pch	0.001	0.003
BTB/POZ_Alm 2	0.001	0.003
Multidrug resistance associated protein_Alm	0.001	0.003
Hypothetical protein 1_Alm	0.001	0.002
Transposable element 3_Pch	0.001	0.002
Receptor kinase 3_Alm	0.001	0.002
Carboxylesterase_Pch	0.001	0.002
Detoxification 45_Alm	0.001	0.002
Transposable element 2_Alm	0.001	0.002
Uncharacterized 1_Pch_At3g47530_Alm	0.001	0.002
Uncharacterized_Alm	0.001	0.002
Uncharacterized 8_1_Alm	0.001	0.002
Uncharacterized 2_Alm 2	0.001	0.002
Exocyst complex component_Pch	0.001	0.002
Auxin transport big_Pch	0.001	0.002
ADP glucose pyrophosphorylase large subunit 1_Alm	0.001	0.002
Paladin 1_1_Pch	0.001	0.002
Transposable element 2_Pch	0.001	0.002
Putative Kinase_Alm	0.001	0.002
Uncharacterized 1_Alm	0.001	0.001
Retrovirus-Related Pol_Alm	0.001	0.001
ATP phosphoribosyl transferase 1 1_Pch	0.001	0.001
Paladin 1_2_Pch	0.001	0.001
QUIRKY_Pch	0.001	0.001
Shoot gravitropism_Alm	0.001	0.001
Uncharacterized 8_1_Pch	0.001	0.001
QUIRKY_Alm	0.001	0.001
Aspartic protease_Pch	0.001	0.001
Uncharacterized 2_Pch	0.001	0.001

*Main effect describes the sensitivity of the NBF outcome (NBF or no-NBF) to the gene target expression represented as normalized Cq values. Total effect represents the total contribution to the variance of the outcome form all the gene targets that are considered in the prediction of NBF. The target maximum standard error for estimation of effects was 0.010.*

### Annotation of Potential Target Genes

Genes identified based on both association with DMRs and differential expression between the NBF- and no-NBF hybrids were further annotated to determine homology between the peach and almond genome sequence, as well as to determine putative functions. The genes: *BTB/POZ domain containing protein FBL11*, *Rust resistance kinase Lr10, Serine/threonine-protein phosphatase 7, Hypothetical Protein 3*, *Hypothetical Protein 4*, and *Uncharacterized* 2, as well as the transposable elements *Retrovirus-related Pol polyprotein from transposon TNT 1-94* and *Non-LTR retroelement reverse mRNAase* were selected as potential targets due to their association with a DMR and their expression pattern profiles. Nucleotide and protein alignments were performed for each gene using both the almond and peach sequences if available (Supplementary Figures 1A–D).

## Discussion

The goal of this study was to identify potential genomic signatures with modified gene expression and associated with differential DNA-methylation and Non-infectious Bud Failure (NBF) exhibition in almond to create a list of gene targets for almond breeding programs to mitigate the disorder. To accomplish this, we analyzed the methylation profiles in a subset of a population of almond × peach interspecific hybrids where NBF-exhibition is segregating 1:1. By comparing methylation profiles in individuals exhibiting NBF symptoms to those that are not currently exhibiting NBF, we were able to identify several regions of differential methylation and their associated genes that putatively play a role in NBF-exhibition in almond. Following identification of these genes, quantitative RT-PCR was performed to assess expression patterns in both NBF hybrids and no-NBF hybrids.

The material used in this study comes from an interspecific hybrid population of almonds resulting from a cross between an NBF-positive almond cv. ‘Carmel’ and a peach breeding selection ‘40A-17,’ which has not shown any NBF symptom as described in almond. This cross was made in 2012 and repeated in 2014, and progeny were planted at the Wolfskill Experimental Orchards at UC Davis. To select individuals for sampling for both the methylation profiling and gene expression studies, trees were identified based on apparent symptoms of NBF. For almond-peach hybrids, rough bark is the primary indicator of bud failure, but additional symptoms include “crazy top” appearance and “rat tailing.”

### Whole-Genome Methylation Profiling Reveals Differential Patterns in Hybrids Exhibiting Non-infectious Bud Failure Compared to Those That Are Bud Failure-Free

Whole-genome DNA methylation was analyzed to identify potential patterns associated with the exhibition of NBF in almond. NBF has previously been hypothesized to be an epigenetic disorder, as no causal agents or underlying mechanisms contributing to its development have been identified (Kester and Jones, 1970; Socias et al., 2017). Further, a study by Fresnedo-Ramírez et al. (2017) showed that NBF is not independent of methylation status by analyzing whole-genome DNA methylation using a methylation-sensitive AFLP approach. Additional work has been done exploring the association between NBF and DNA methylation utilizing a set of identical twin almonds with divergent NBF-exhibition (D’Amico-Willman et al., 2021). This divergent exhibition was also shown not to be independent of the methylation status of the twin with respect to BF-exhibition (D’Amico-Willman et al., 2021).

In the present study, a pattern of differential whole-genome methylation is observed when comparing NBF hybrids and no-NBF hybrids using the almond reference genome for alignment and methylation calling. Interestingly, this pattern is not observed when using the peach reference genome for alignment, which coincides with the observation that peach, regardless of its relatedness and ability to hybridize with almond, has not been reported to develop NBF (Kester, 1978). This result suggests that the differential methylation patterns and associated gene features observed in the interspecific hybrids and associated with NBF-status may be due to genetic contribution from the NBF-positive ‘Carmel’ almond parent.

Previous studies have demonstrated that NBF is inherited in progeny from both sexual reproduction and vegetative propagation (Kester and Jones, 1970), giving rise to the hypothesis that NBF has an epigenetic component underlying its development. The genome-wide methylation data generated in this study supports the hypothesis that NBF-potential is inherited from the almond parent and seems to arise from the genomic contribution of the almond parent in the interspecific hybrids tested.

### Identification of Regions of Differential Methylation Associated With Non-infectious Bud Failure Exhibition

Following the analysis of whole-genome DNA methylation patterns in the almond × peach hybrid population, specific regions of differential methylation within the genome were identified. These regions are referred to as differentially methylated regions (DMRs) and represent regions within the genome where there is significant differential methylation between two or more groups being analyzed. In this study, DMRs were identified that show significant differences in the level of cytosine methylation between the NBF and no-NBF hybrids. In total, 115,635 DMRs were identified based on alignment to the almond reference genome, and 126,800 DMRs were identified based on alignment to the peach reference genome. A high number of epigenetic polymorphisms were also observed in the Fresnedo-Ramírez et al. (2017) study, suggesting that almond, and possibly *Prunus spp.* in general, shows dynamic patterns of DNA methylation that have the potential to influence a variety of phenotypes.

When categorized by methylation context [CG, CHG, and CHH (H = A, T, or C)], the highest number of DMRs occurred in the CG context for both the almond and peach alignments. CG is the most common type of DNA methylation in plants (Lister et al., 2008), including Rosaceous species; thus, in our study, the high ratio of CG methylation, as well as the number of DMRs in this context, are not unexpected. The DMRs were further categorized as either hyper- or hypomethylated in the NBF hybrids relative to the no-NBF hybrids. An enrichment of hypomethylated DMRs was observed on chromosomes seven and eight in both the peach and almond alignments. Interestingly, a recent study showed that CG hypomethylation in rice (*Oryza sativa*) led to a transpositional burst of transposable elements in the genome (Hu et al., 2020). Further work is needed to examine the possible impact of this CG hypomethylation in NBF hybrids. Particularly interesting is the methylation pattern found on chromosomes seven and eight, which might be related to the translocation of transposable elements.

In addition to an observed enrichment of hypomethylated CG DMRs in the hybrids, CHH DMRs also showed a pattern warranting further investigation. In the almond alignment, there are a higher number of hypermethylated DMRs in the CHH context compared to hypomethylated DMRs; however, in the peach alignment, this pattern is reversed. Therefore, patterns of differential DNA methylation unique to almond could indicate a possible role in NBF development, as well as a putative epigenetic mark associated with NBF, which is desirable to know and further characterize. Interestingly, the presence of CHH islands in plants has recently been described, and hypermethylation of these regions was shown in grasses to have an impact on downstream gene expression (Martin et al., 2021). In addition, CHH methylation has been shown to be involved in vegetative growth. In a study in rice, CHH hypermethylation was observed in advance of changes in the shoot apical meristem (Higo et al., 2020). Future work is needed to investigate the presence and potential role of CHH islands in almond and their possible functions and impact on NBF potential. CHH methylation is biologically intriguing given that it is independent of demethylase activity and chromatin structure; therefore, its characterization may require additional tools to better understand its dynamics (Harris and Zemach, 2020). Additionally, CHH methylation has been shown to be involved in regulating the expression of transposable elements in plant genomes (Harris and Zemach, 2020; Wang and Baulcombe, 2020; Xin et al., 2021). Prediction analysis using gene expression data in this study indicates expression of *transposable element 1* from both almond and peach are highly predictive of NBF exhibition. Further, the differential expression patterns of *transposable element 1* from almond in NBF compared to no-NBF trees are opposite that observed for *transposable element 1* from peach. It is possible that dysregulation of transposable elements in the genome contributes to the NBF phenotype and that this regulation could be associated with differential patterns of CHH methylation. This theory will need to be tested in additional germplasm, including different cultivars exhibiting NBF.

### Expression and Annotation of Genes Associated With Highly Significant Differentially Methylated Regions

In general, the gene expression profile showed a pattern where a small proportion of the hybrids resemble gene expression of the peach control (‘Okinawa’) while a larger portion resembles the gene expression of the almond control (‘Carmel’). This pattern can be seen in the heatmaps in Figure 3A, and this expression does not necessarily fit with the categorization of the hybrids with respect to NBF symptoms. Although this result may seem to be a source of noise in our analysis, the pattern may shed light on the genomic interaction occurring in the interspecific hybrids. It has been shown that in interspecific hybrids (and allopolyploids), a relationship of dominance in terms of gene expression occurs between the two subgenomes interacting in the interspecific hybrid (Edger et al., 2017; Glombik et al., 2020). This dominance seems to be driven by the inheritance of epigenetic patterns related to the density and proliferation of transposable elements as well as their methylation levels, which also influence the expression of nearby genes (Edger et al., 2017). Results in Edger et al. (2017) showed that gene expression is greatly altered because of altered levels of methylation in CHG and CHH sites, which coincides with the results seen in our study.

The general pattern of gene expression observed in this study might suggest a pattern in subgenome dominance inheritance. This finding warrants additional research because this inheritance pattern was seen in a subset of the progeny of interspecific hybrids produced from two heterozygous parents, while previous studies analyzed one individual at a time (Glombik et al., 2020). In *Prunus*, interspecific hybrids are used for a wide variety of purposes: from simply generating additional phenotypic variation, to the use of hybrid vigor (e.g., in rootstocks), to the introgression of traits of interest (such as gametophytic self-compatibility from peach into almond). As haplotype-based genome assemblies become more affordable, these questions can be more readily addressed.

In relation to the potential impact of differential methylation on the expression of nearby or overlapping genes, the top 26 most significant DMRs were selected, and associated genes in peach and/or almond alignments were identified. In some cases, DMRs were identified in both the almond and peach alignments that were highly significant, while in others, the DMR was only present in the peach or almond alignment. The closest gene to each DMR was selected for expression analysis, and primers were designed for both the almond and peach copy of each gene if present. A ‘Carmel’ almond clone exhibiting NBF was selected as a positive control, and the peach cultivar ‘Okinawa,’ which is known to be free of NBF symptoms, was used as a negative control for comparison in the qPCR analyses. Results of this analysis revealed several target genes with expression patterns associated with NBF-exhibition in NBF hybrids compared to no-NBF hybrids.

Our results suggest that, based on the classification of NBF hybrids and no-NBF hybrids of the individuals analyzed, the expression pattern of specific genomic elements might be associated with the exhibition of NBF. These include (Figure 3B) the genes *BTB/POZ domain containing protein FBL11, Rust resistance kinase, Serine/threonine-protein phosphatase 7, Hypothetical Protein 3, Hypothetical Protein 4*, and *Uncharacterized 2*, as well as the transposable elements *Retrovirus-related Pol polyprotein from transposon TNT 1-94* and *Non-LTR retroelement reverse mRNAase*, which, according to the bootstrap analysis performed, contribute close to 70% of the prediction of NBF. These genes of interest occurred in all three methylation contexts. *Hypothetical proteins 3* and *4*, which were harbored by the same DMR, are in the CG context, *Non-LTR retroelement reverse mRNAase* is in the CHG context, and *transposable element 1*, which is associated with a *retrovirus-related Pol polyprotein from transposon TNT 1-94*, is in the CHH context. These elements together contribute close to 63% of the prediction of NBF in the samples tested, making them candidates for further exploration of their possible involvement in NBF.

Non-infectious Bud Failure was previously shown to be associated with heat stress where high levels of NBF-exhibition in orchards follow extreme temperatures in the previous growing season (Kester and Asay, 1978; Hellali and Kester, 1979). The exact mechanisms associated with heat stress and NBF-exhibition are not known, but previous work suggests potential links to alterations in hormone levels and metabolic alterations associated with the mitochondrion (Hellali and Kester, 1979; Durzan and Kester, 1997). This is the first report where expression of specific genes is being assessed in association with DNA methylation status to interrogate NBF. In conjunction, a subset of genes and transposable elements found in this study integrate a group of genomic elements involved in processes related to stresses, particularly abiotic. Below, we present information on genes and transposable elements found in this study with differential gene expression among the NBF hybrids.

### Possible Gene and/or Transposable Element Activity to Predict Non-infectious Bud Failure

Interestingly, both annotated genes and transposable elements have been linked to stress responses in plants and some are also related to genome structure, though their roles have not been reported in Rosaceae spp. The BTB/POZ proteins have been widely linked to selective ubiquitination and kinase activation (Gingerich et al., 2005) since they are transcriptional regulators with an inferred involvement in chromatin conformation. Those containing F-box proteins such as FBL11 may have an involvement in ubiquitin-dependent protein catabolic processes. For example, in Arabidopsis (check locus AT2G36370, Berardini et al., 2015), overexpression of this gene is reported when the plant is heated to 38°C, with notably high expression in the central zone of the shoot apex. Additionally, this gene might influence DNA methylation and heterochromatinization, as seen in the self-incompatibility (*S*) alleles of apple (Wang et al., 2012).

Another annotated gene that may act in regulation of defense responses and signal transduction is *rust resistance kinase Lr10* (homolog of AT3G47570 in Arabidopsis, Berardini et al., 2015). This gene codes for a leucine-rich repeat protein kinase family protein involved in protein phosphorylation (Nemoto et al., 2011) and is expressed in several tissues, producing mobile RNAs. In Scots pine (*Pinus sylvestris* L.), a similar receptor-like protein kinase was found to have a possible function in vascular tissue development, specifically the phloem (Ávila et al., 2006), which would be relevant to the necrosis of the vascular tissue which is a main symptom of NBF in almond. Additionally, *serine/threonine-protein phosphatase 7* (PP7) is a protein with phosphatase activity and metal ion binding (Kutuzov et al., 1998), which has been linked to thermotolerance via expression of heat shock proteins (Liu et al., 2007) as well as stomatal aperture (Sun et al., 2012).

It is well recognized that long terminal repeat (LTR) retrotransposons play a role in genome dynamics and genome stability in relation to stress responses (Grandbastien, 1998; Lanciano and Mirouze, 2018; Roquis et al., 2021). In plants, transposable elements TNT1, such as retrovirus-related Pol polyprotein from transposon TNT 1-94, first identified in tobacco (Grandbastien et al., 1989), have been associated with genome modifications such as structural variations (Hernández-Pinzón et al., 2012). Thus, these transposable elements may drastically alter expression of nearby genes and influence phenotypes. Due to their mutagenic ability, some of these transposable elements are used to perform insertional mutagenesis in some crops (Tadege et al., 2005; Thieme and Bucher, 2018). Specifically, TNT 1-94 has been associated with response to drought and recovery in *Pinus halepensis* (Fox et al., 2018). The mechanism that the hosts use to ameliorate the effects of insertions is based on DNA-methylation surrounding the insertion site which prevents binding of H2A.Z histone variants in regulatory elements (Hernández-Pinzón et al., 2012) and promotes changes in gene expression.

Non-LTR retrotransposons have been less studied in plants (Schmidt, 1999) despite their known role in genomic evolutionary processes (Noma et al., 1999; Setiawan et al., 2020). In mammalian systems, these elements are considered silent because of DNA methylation and tend to be inserted in gene-rich regions (Carnell, 2003). More recently, this type of transposable elements is suggested to be linked to stress activation in Scots pine (Voronova et al., 2014) and even influence the production of anthocyanin in chili pepper (Jung et al., 2019).

The hypothetical proteins identified in this study strengthen the association of NBF exhibition with stress responses. Annotation of the *hypothetical proteins 2, 3*, and *4* show that for *hypothetical protein 2*, no significant similarities with previous records in databases were found. *Hypothetical protein 3* is related to a Chaperone DnaJ-domain superfamily protein with homology to locus AT2G18465 or DJC72 in Arabidopsis (Berardini et al., 2015), which is in the chloroplast and expressed in the guard cell and the shoot apex. This clade of proteins has been identified only in flowering plants and is well conserved. The function may be related to protein recruiting in the chloroplast (Chiu et al., 2013). *Hypothetical protein 4* is related to a retrotransposon or some sort of transposable element. The structure of the protein suggests a DNA/RNA polymerase superfamily protein fitting with the theoretical function of nucleic acid binding and DNA integration (UniProt Consortium et al., 2021). This finding emphasizes the relevant role of transposable elements associated with NBF.

Annotation of *Uncharacterized protein 2* suggests this gene codes for an O-methyltransferase family protein that may be involved in the melatonin biosynthesis. Its homologous gene in Arabidopsis (AT4G35160, or acetyl serotonin methyltransferase *ASMT1*, Berardini et al., 2015) has been reported to be expressed in the epidermal L1 layer in the shoot apex. The melatonin biosynthesis pathway was recently shown to be associated with both abiotic (Wang et al., 2021) and biotic (Zhu et al., 2021) stress responses (Bhowal et al., 2021). Members of the melatonin pathway have been shown to be involved in responses to osmotic, drought, and heat stresses (Bhowal et al., 2021), and many of these genes are regulated through miRNAs. In rice, induction of the homologous gene, *OsASMT1*, occurred following exposure to the cytokinin benzylaminopurine (Bhowal et al., 2021) which is known to stimulate cell division. In tomato, melatonin enhances the expression of heat shock proteins following heat stress, improving thermotolerance (Xu et al., 2016; Wang et al., 2018), while in grape, the role of *ASMT1* was related to enhanced ozone tolerance in leaves (Liu et al., 2021).

Future studies focused on genome structure are required to understand the role of transposable elements in development of NBF in almond, including identifying insertion sites and determining the impact of insertions on expression of nearby genes. One promising technique is mobilome-seq (Thieme and Roulin, 2021), which would enable us to identify and track transposable element insertions across the almond genome and observe affected genes, as some of these elements can trigger structural variations of up to 15 kb (Tadege et al., 2005). It would also be interesting to test how the interspecific hybrids respond to heat stress and what impact that stress might have on the expression of the genes identified in this study.

In this study, expression of genes potentially involved in the development of Non-infectious Bud Failure (NBF) in almond were identified by analyzing F_1_ interspecific almond × peach hybrids. By profiling the DNA-methylation and identifying differentially methylated regions (DMRs) in hybrids divergent for the exhibition of NBF, genetic features with contrasting DNA-methylation were selected for gene expression studies. To investigate expression, homeologs of almond and peach were selected, and expression was tested in a set of hybrids with and without NBF symptoms: “crazy top,” rough bark, and rat tailing. Overall gene expression patterns did not associate with NBF symptoms, but a pattern of gene expression was observed and deserves further investigation. Specific gene features and transposable elements were found to be predictive of NBF exhibition in these interspecific hybrids and these genomic components seem to have relevant roles in stress response. These genomic features will be further investigated to uncover their potential role in NBF development for use in screening almond breeding selections for NBF-potential.

## Data Availability Statement

The original contributions presented in the study are publicly available. These data can be found in NCBI-SRA under the BioProject PRJNA770308 – Identification of putative markers of noninfectious bud failure in almond (*Prunus dulcis* [Mill] D.A. Webb) through genome-wide DNA methylation profiling and gene expression analysis in an almond peach hybrid population.

## Author Contributions

TG developed the germplasm. JF-R, KD’A-W, GS, and TG designed the study. KD’A-W, EA, GS, and BA processed samples, generated, and analyzed the data. KD’A-W and GS wrote the first draft of the manuscript. EA and BA contributed to methods section. All authors contributed to data collection and preliminary analysis, manuscript revision, and approved the submitted version.

## Conflict of Interest

The authors declare that the research was conducted in the absence of any commercial or financial relationships that could be construed as a potential conflict of interest.

## Publisher’s Note

All claims expressed in this article are solely those of the authors and do not necessarily represent those of their affiliated organizations, or those of the publisher, the editors and the reviewers. Any product that may be evaluated in this article, or claim that may be made by its manufacturer, is not guaranteed or endorsed by the publisher.

## Footnotes

1
https://www.neb.com/tools-and-resources/interactive-tools/dna-sequences-and-maps-tool
