# Supplementary figures and images for: Identification of Putative Markers of Non-infectious Bud Failure in Almond [Prunus dulcis (Mill.) D.A. Webb] Through Genome Wide DNA Methylation Profiling and Gene Expression Analysis in an Almond × Peach Hybrid Population
Source: Front Plant Sci. 2022 Feb 14;13:804145. doi: 10.3389/fpls.2022.804145 (PMC8882727; doi:10.3389/fpls.2022.804145)

## Slide 1
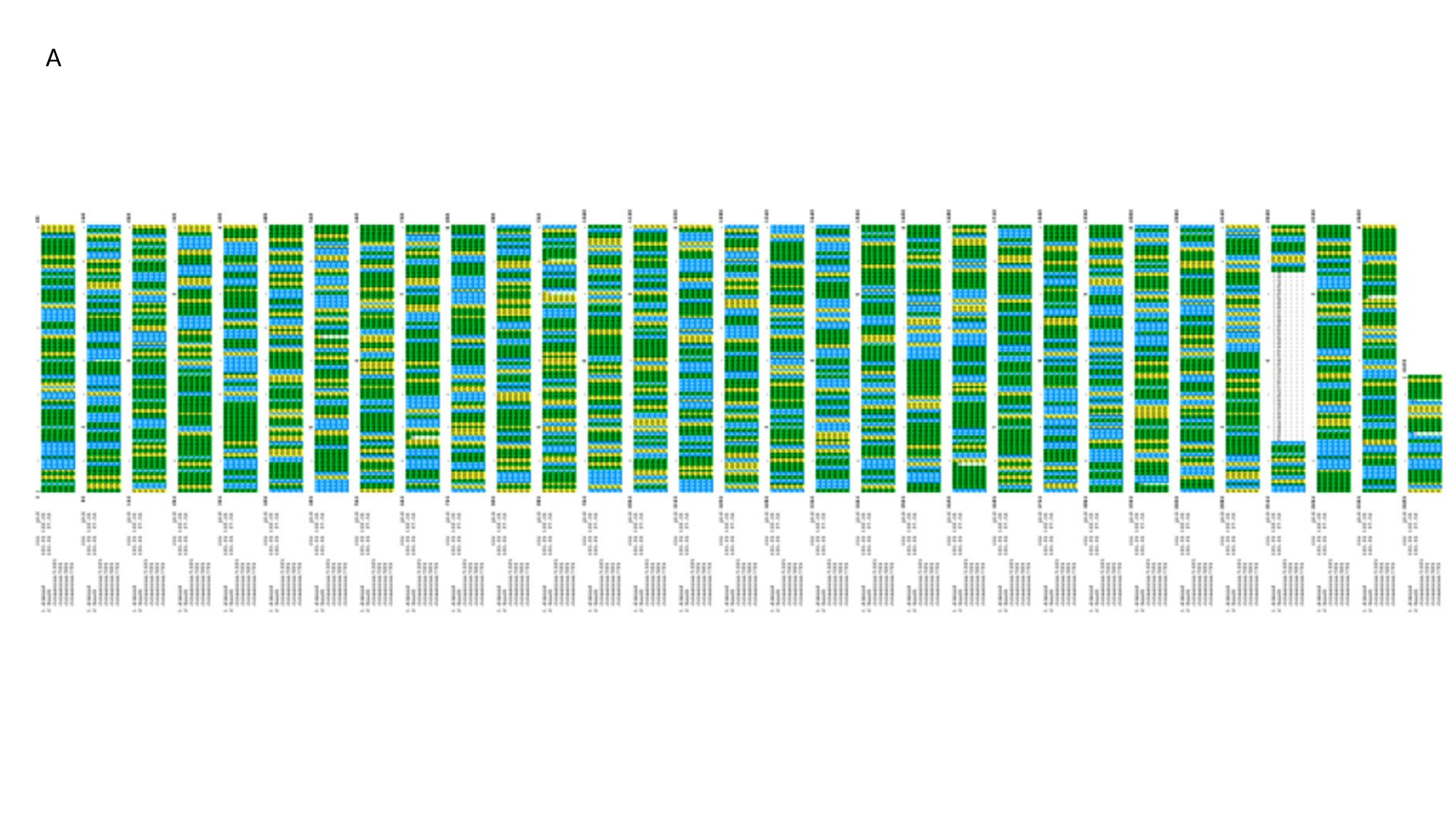

A

## Slide 2
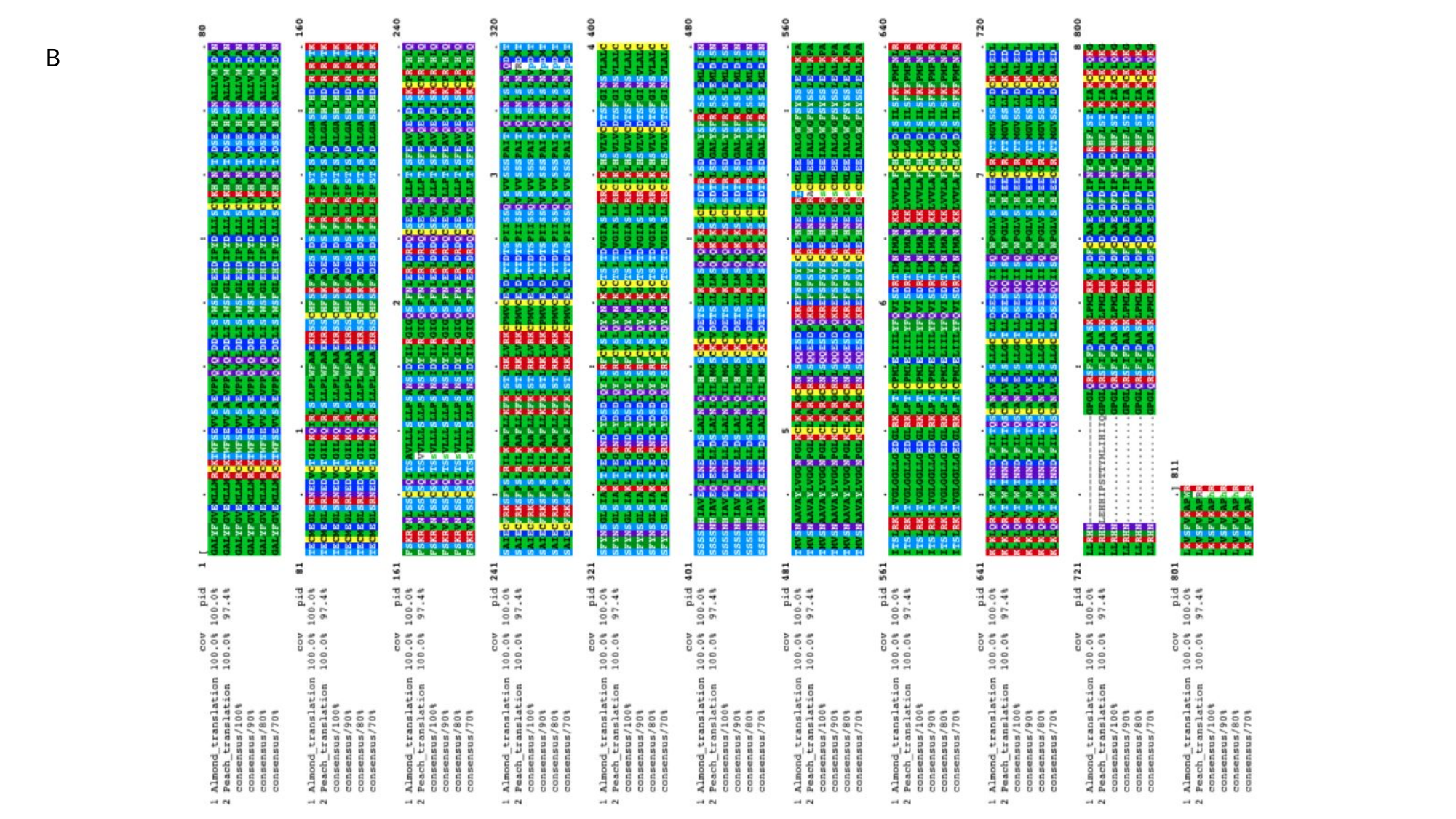

B

## Slide 3
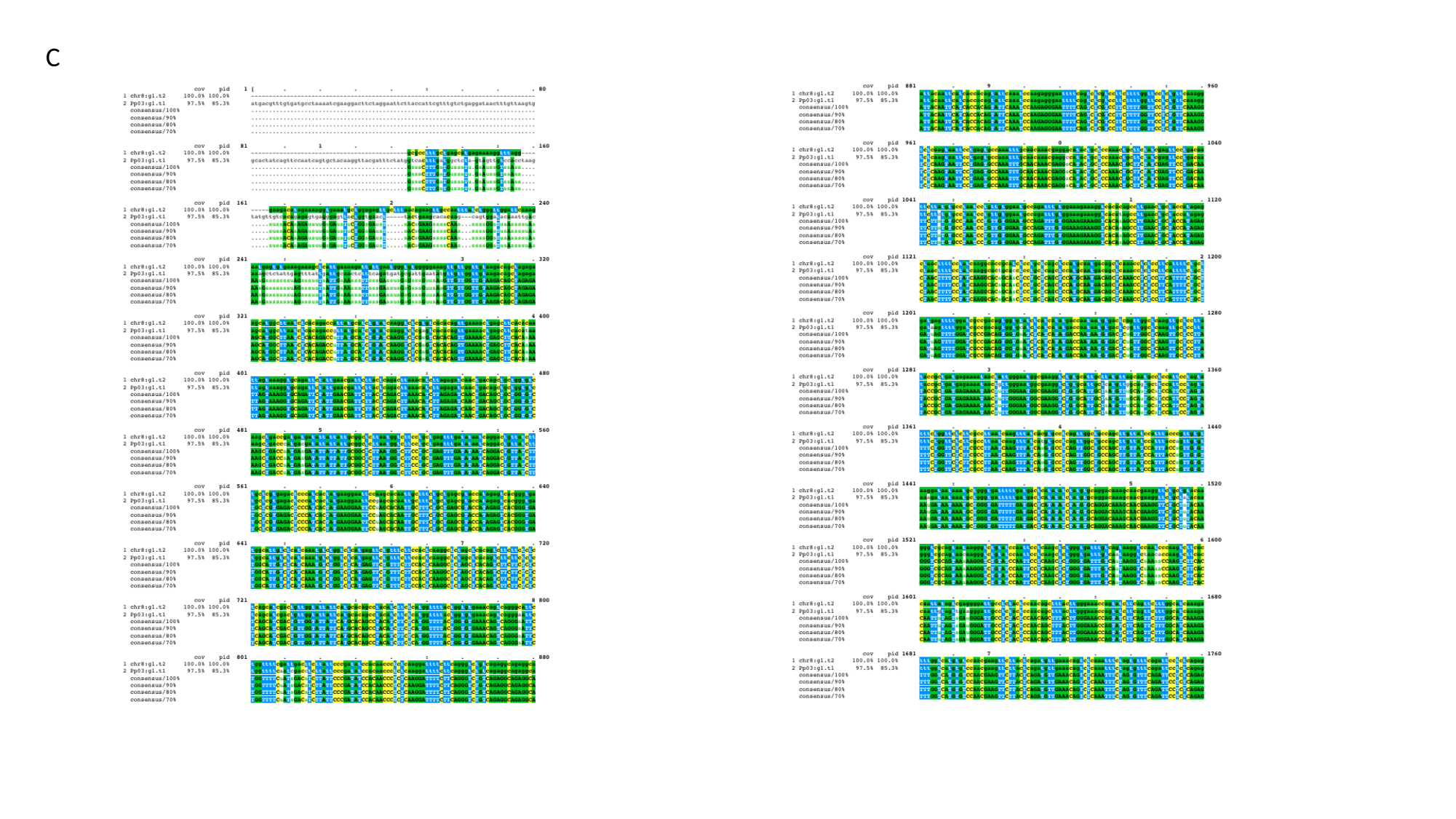

C

## Slide 4
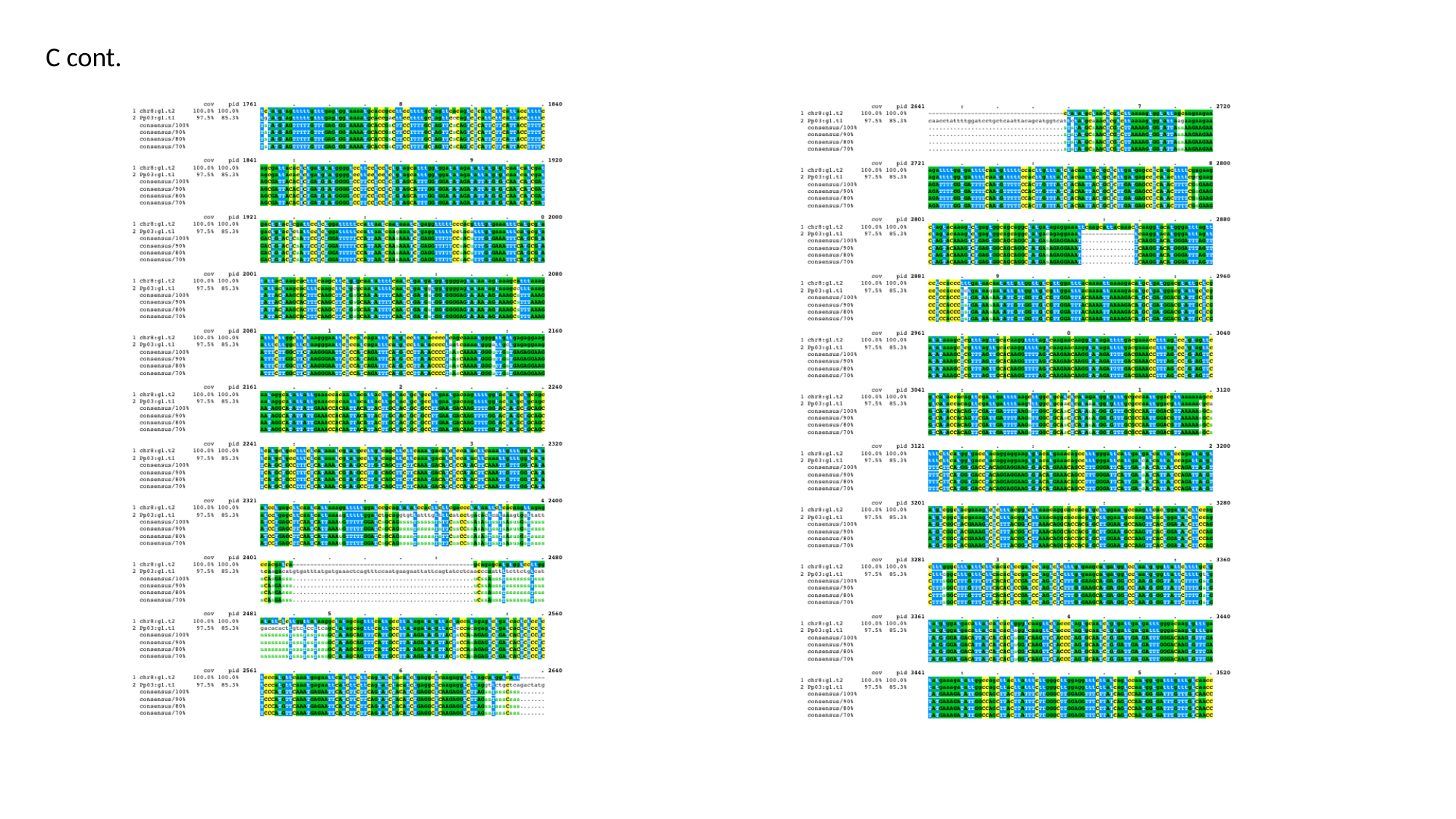

C cont.

## Slide 5
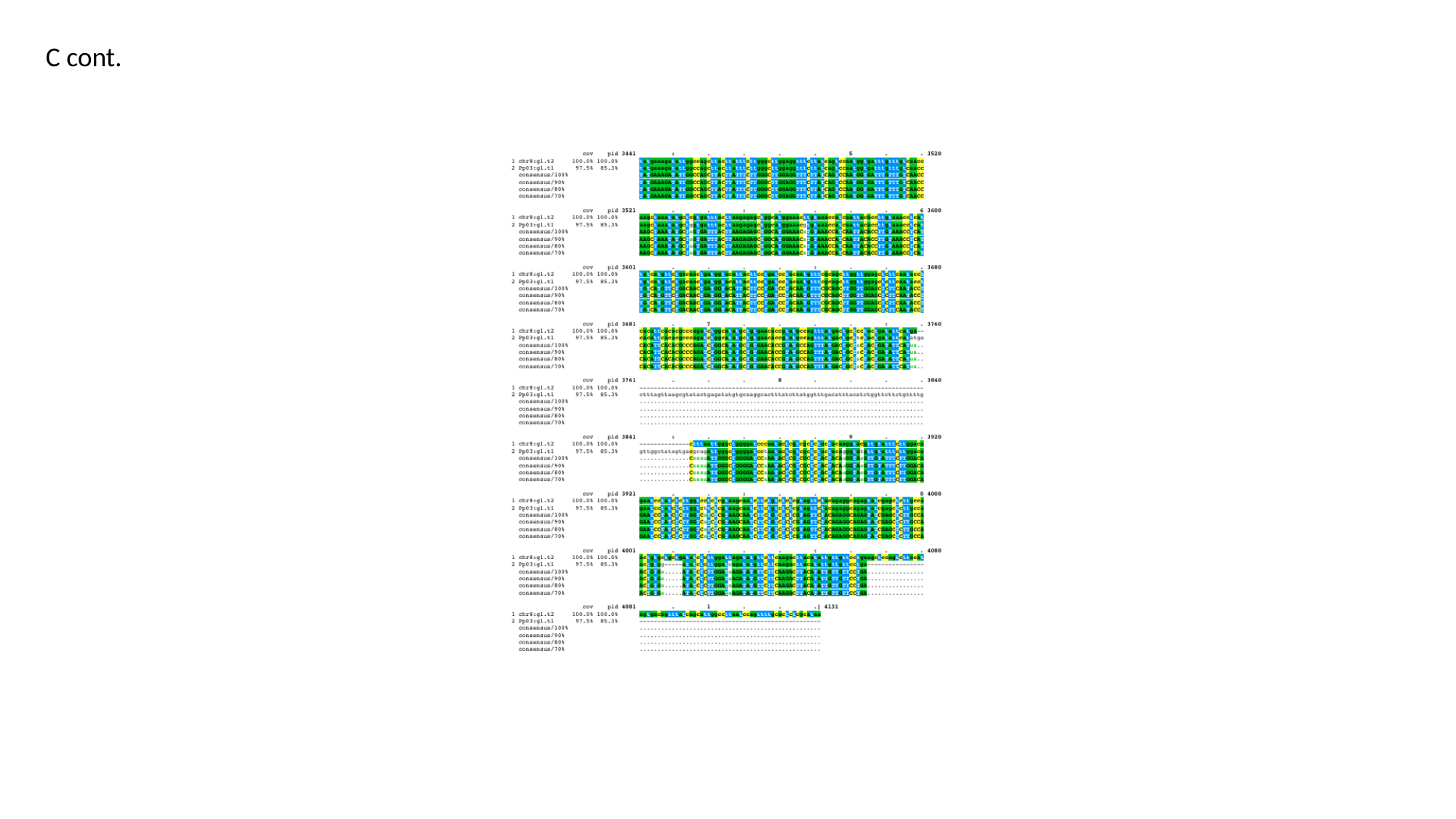

C cont.

## Slide 6
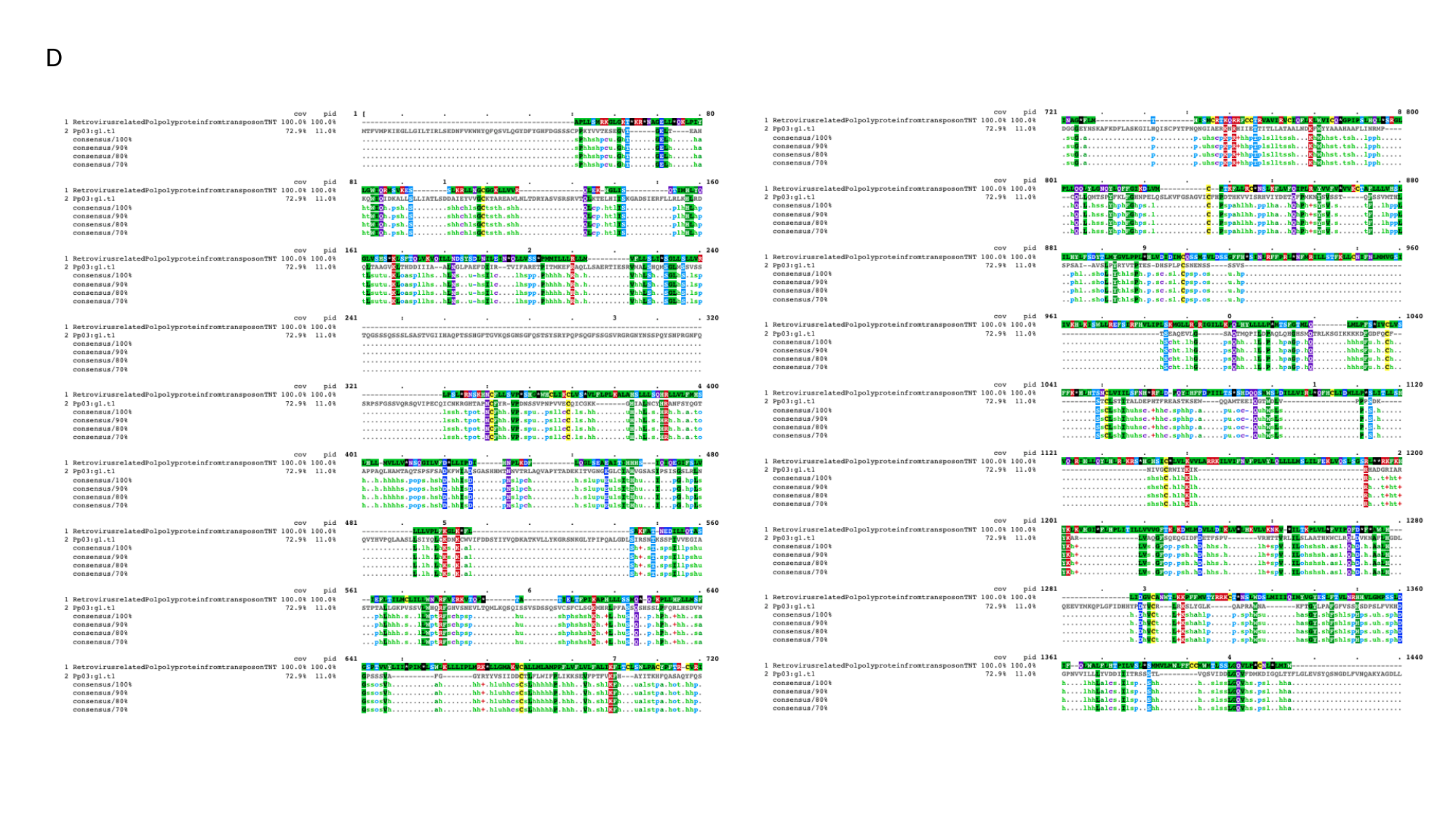

D

## Slide 7
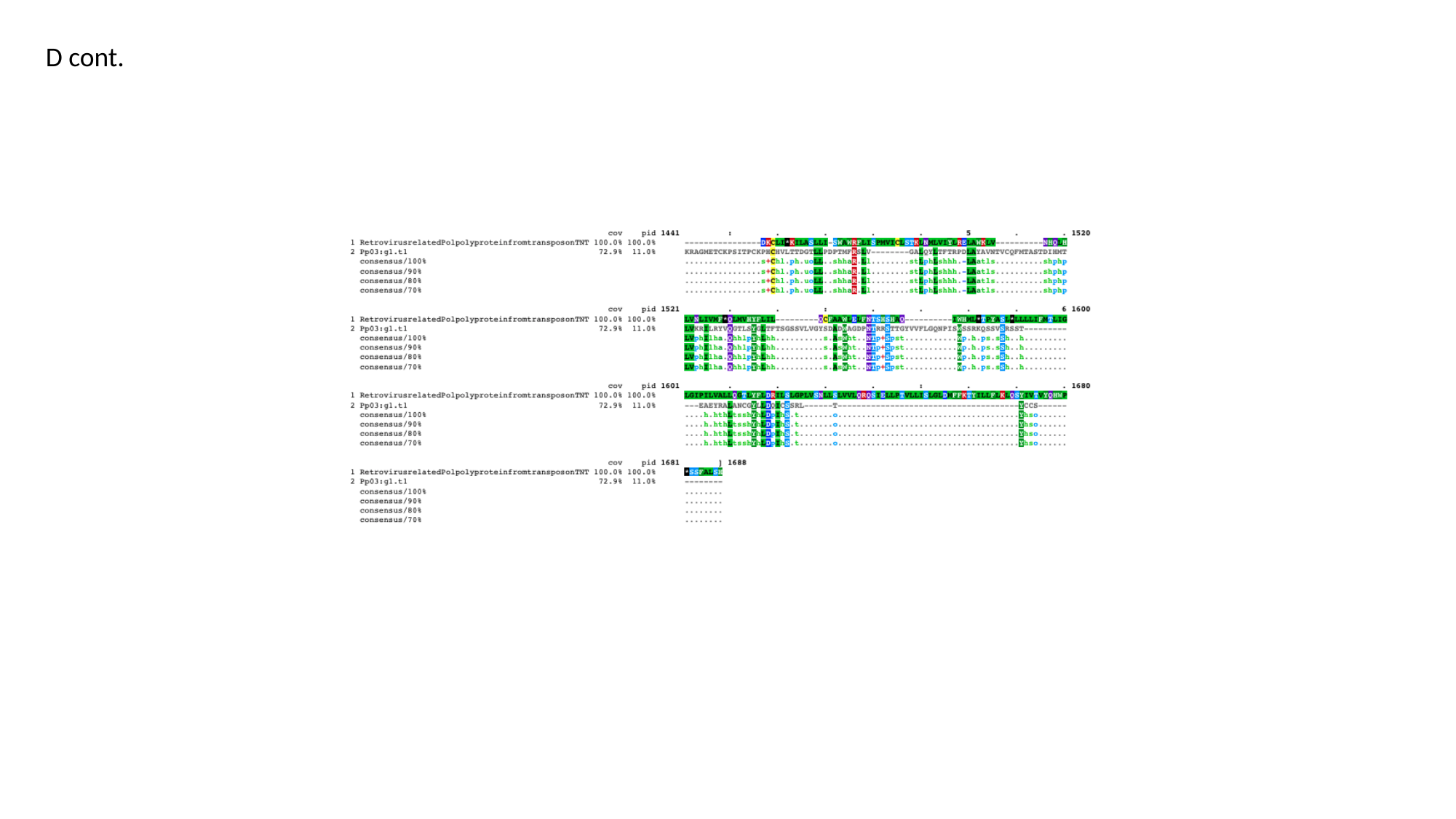

D cont.

Supplement: Supplementary file 1 [file Presentation_1.PPTX]
