# Supplementary material for: Identification of Putative Markers of Non-infectious Bud Failure in Almond [Prunus dulcis (Mill.) D.A. Webb] Through Genome Wide DNA Methylation Profiling and Gene Expression Analysis in an Almond × Peach Hybrid Population
Source: Front Plant Sci. 2022 Feb 14;13:804145. doi: 10.3389/fpls.2022.804145 (PMC8882727; doi:10.3389/fpls.2022.804145)
Supplement: Supplementary file 2 [file Data_Sheet_1.docx]

Supplementary Material

# Supplementary Data

Supplementary Material should be uploaded separately on submission. Please include any supplementary data, figures and/or tables. All supplementary files are deposited to FigShare for permanent storage and receive a DOI.

Supplementary material is not typeset so please ensure that all information is clearly presented, the appropriate caption is included in the file and not in the manuscript, and that the style conforms to the rest of the article. To avoid discrepancies between the published article and the supplementary material, please do not add the title, author list, affiliations or correspondence in the supplementary files.

# Supplementary Figures and Tables

## Supplementary Figures

**Figure S1.** Nucleotide and amino acid alignments of select genes showing divergent DNA methylation profiles and expression patterns when comparing almond × peach hybrids that exhibit non-infectious bud failure compared to those that are bud failure-free. Sequences for the genes are from either the ‘Nonpareil’ almond reference genome or the ‘Lovell’ peach reference genome. A consensus sequence is reported below, including homology between the sequences. Genes analyzed include: *BTB/POZ domain containing protein FBL11* **(A** – nucleotide; **B** – amino acid**)**, and *retrovirus-related Pol polyprotein from transposon TNT 1-94* **(C** – nucleotide; **D –** amino acid**)**.

## Supplementary Tables

**Table S1.** List of primer sequences used for quantitative RT-PCR to analyze expression of 25 genes in the almond × peach hybrid individuals. For each gene, the gene ID is provided based on genome annotation of the corresponding reference genome. The methylation context is provided for each DMR associated with the gene, and the directionality of the DMR (hyper or hypomethylated in BF compared to BF-free trees) is listed. The alignment column provides information on which genome alignment produced the DMR associated with each gene. If a DMR was found in both the peach and almond genome alignments, it is listed as Peach/Almond. The length of the DMR is listed in base pairs for each alignment, and the number of cytosines in each DMR is provided. The genomic coordinate for each DMR is also listed for both the almond and peach alignments. Finally, the annealing temperature for the qRT-PCR experiments is provided.

**Supplementary Table S2.** Results from whole genome enzymatic methyl-seq sequencing and subsequent alignment to the almond, peach, and lambda reference genomes. The number of reads indicates the total number of reads generated following sequencing. Reads aligned to the peach and almond reference genomes are shown including the mapping efficiencies for both alignments. Finally, the methylation conversion efficiency is shown for each sample based on alignment to the lambda genome and calling for methylated cytosines in that alignment.

| **Tree ID** | **Number of Reads** | **Reads Aligned to Peach Ref** | **Reads Aligned to Almond Ref** | **Peach Mapping Efficiency** | **Almond Mapping Efficiency** | **% CG Meth** | **% CHG Meth P/A** | **% CHH Meth P/A** | **Lambda Conversion Efficiency** |
| --- | --- | --- | --- | --- | --- | --- | --- | --- | --- |
| NBFHyb1 | 27,718,050 | 8,800,176 | 15,399,911 | 31.7% | 55.6% | 34.0% P 54.0% A | 11.2% P 23.3% A | 1.7% P 2.9% A | 99.6% |
| NBFHyb2 | 38,528,897 | 16,251,893 | 17,214,043 | 42.2% | 44.7% | 43.4% P 45.0% A | 17.9% P 20.3% A | 2.0% P 2.1% A | 99.3% |
| NBFHyb3 | 35,191,290 | 14,560,151 | 15,538,407 | 41.4% | 44.2% | 39.9% P 40.8% A | 16.3% P 18.1% A | 2.1% P 2.0% A | 99.9% |
| no-NBFHyb1 | 26,188,301 | 11,532,647 | 11,981,803 | 44.0% | 45.8% | 41.8% P 42.9% A | 17.0% P 18.9% A | 2.1% P 2.1% A | 99.7% |
| no-NBFHyb2 | 53,260,015 | 16,385,368 | 29,490,424 | 30.8% | 55.4% | 33.5% P 53.6% A | 10.9% P 22.9% A | 2.7% P 2.9% A | 99.4% |
| no-NBFHyb3 | 45,318,169 | 18,838,761 | 19,905,477 | 41.6% | 43.9% | 40.0% P 41.9% A | 16.7% P 18.7% A | 2.5% P 2.5% A | 99.1% |

**Supplementary Table S3.** Mean normalized Cq values for NBF-hybrids and no-NBF hybrids. Fifty-three DMR associated genes mean normalized Cq values were evaluated utilizing QuantStudio 3 qPCR machine and ThermoCloud RQ software.

**Supplementary File S1.** Nucleotide sequences in fasta file format of the genomic components considered in this study.

>Pp01:g1.t1 aspartic Protease Guard Cell 1

ATGCTCCTCCTCTCGTCTCCTCATCCAAATCTCCTCCTCTCCTCTCCACCCCATAAATTTGCCTTTGTTTTCTTTCAGTGTCCTTTACGTGATTCCTGCAATTATGCTTACTTTTCTCCGCTCTTAAATAAATCAACAGTCCTCAAATCTCACACTTCACCTCCCCACCTCCCCACCTCACACTCTCTTCCAGACCTCAACGCCCAAAATGGGTTTCCTCTCCTACATTTTCCTTCTCTTTGCTTTCTTTTGCTGTACTTCCAACTAGCTCACTCTCGAAGCTCACCGCTTAGCTCCAAAACGACAGTGCTCGACGTCGCTGCTTCAATTCATACGACCTTAAACGCCCTGTCGTCGGAGTCTCACACGCAGGCATTGAGCCAACAAGACCAGAGCTCCTCAGCTGCTTCTTCACTTTCTCTGCCGCTACATTCTCGAATTTCACTTCACAAGCCTTCCCACAGTGACTACAAGTCGCTCACTTTGGCTCGACTCGAGCGTGACTCAGCCCGAGTCAGATCTCTCACAACCCGGTTAGATCTGGCTCTTCAAGGCATTGCCACGTCGGATCTTAAACCCGTGGATACCGGCAACGGGTTGGAGCTTGAGGAAGCCAAGGGTTTTGAGGGTCCCGTCATTTCCGGGACGAGTCAGGGAAGCGGCGAGTACTTCTCCCGAGTCGGAATCGGCAAACCACCGAGTCCGGCCTACGTGGTGCTCGACACGGGTAGTGACGTCAGCTGGGTGCAGTGCGCACCCTGCGCCGACTGCTATCAACAAGCCGAACCCATCTTCGAGCCGACTTCCTCGGATTCCTTCTCGCCTCTCTCGTGCGAAAACCAACGGTGCAAGTCCCTCGACGTGTTCGAGTGCCGCAACGACACATGTCTCTACGAGGTGTCCTACGGCGACGGCTCCTACACCGTCGGCGACTTCGTCACGGAGACCATCACCATCGGCGGGGTTGCGGCGAAGGACATAGCCATCGGCTGTGGGCATACCAACGAGGGCTTGTTCATCGGCGCGTCTGGGCTCCTCGGACTTGGAGGCGGCCCGTTGTCGTTTCCTTCCCAGCTCAATGCTACGTCATTTTCCTACTGCCTCGTGGACCGTGACTCTGACTCGGCTTCAACTCTTGAGTTTAACTCCCCGCTCCATCCCAATGCCGTGACGGCTCCGTTACGCCGTAACCCTGAGCTCGACACGTTTTACTATATCGGCCTAGCCGGACTGAGCGTAGGCGGCGAGTTGCTACCGATTCCCGAGTCGGCTTTTCAGATAGACGACAGCGGAAACGGCGGAATCATCATCGACTCGGGCACGGCGGTGACTCGGTTGCAGACGGACACTTACAACGCGCTCCGTGATGCGTTCGTGAAAGGCACCAAGGACTTGACGTCCACCCAAGGCGCGGCGCTGTTCGACGTGTGTTACGACTTGTCGTCGAAGAAGAGCGTTGAGGTGCCAACGGTGTCGTTTCACTTCGCGGACGGGAAAGTGTTACCGTTACCGGCTAAAAATTACCTGATACCGATTGACTCGGAGGGGACTTTTTGCTTTGCGTTCGCGCCCACGCCCTCCTCGATGTCAATCATTGGGAATGTCCAACAGCAAGGGACACGTGTCGGTTTCGACATCGCTAATTCGGTGGTTGGGTTCTTCCCCAACCAATGCTAG

>Pp07:g1.t1 BTB POZ Domain Containing Protein FBL11

GGTGCACTTTATTTTGGAGTGGAGATGCTTCTCATGAGATGCAAAACTTGGTTTTCTGAGGTAGTATCGGCGGAGGTGCCACCACAAGTACAATTGGATGATTTGATTTCTATTTGGAGCTTTGGTTTAGAGCATGATATTCCATACGACTTATTACTATCTTGCGTGAAGCACATGAATTTGACAGTAGACAGTGAGATGCATCTTTCCAATGCACTTCTAGTTTGGATCGATGCTAACACAGAATGCATGGAAGGCTTGAGCAGAAATGAAGATGTCTGCACTGGCATTTTGAAACAGATCCGTTTAAGCCTTTTGCCGTTGTGGTTTGCTGCAGAGAAAAGAAGTTCTTGTCATTTTTCTAAGTTTGCTGATGAGAGCATTGATTCAATTTTCAGACTACTGAGAATTCCATCCACTGGCTCAGTAGATGCCTTAGGAGCTAGTCACTTGCATGATTTACGCATTCGGCTGACAAAATTTTCTAAGAGAGTAAACCTTTCAAGTTGCTCACAGATAACGTCAGTGGTGCTACTTTTGTCTTTGCTTCCTTCTGCAAACAGTATCGACTACATACTAAGGGGTATTGGGCAGTCACCATTCAACCTTGAACGTCTCGACAGAGATCAATGTTCGGAAGTACTGAATTTGTTGCCGACTTTGTCTTTTGAAGCAGTACAAGAGGTGGATATTTCCAAGTGTCCAAGGTTGCATCTTCAATCTGCCATTGAGTGCTTCCGCAAATCATTTCCATCTTTAAGAATACTGAAGGCAGCTTTTCTTTTGAAATTCAAGATAAGCACTTTGCGCAAACTGGTGCGGAAATGCCCTATGGTCTGTGAAGTTGACTTAACCACCGACACTAGTCCAATTATATCATCACAAGTGTCCGTTGTATCCTCAAGTCCAGCTATAACACCACAAATATCAAATTTGTCCTTAAATGTTCGGGATATGACTTCTTTTTACAACTCTGGACTATCGATAGCAAAACTCACATTGGAGGGCCGAAATGATCTCTATGATTCAGATCTCCAGTATATCTCCAGATTCTGTGTCTCCTTGCAATACGTAAACCTCAAGGGGTGTACTTCATTAACTGATGTTGGCATAGCAAGTCTTTTACGCAGATGTATTAAGCTACATTCTGTTTTAGTTTGTGATACCTCTTTCGGGATAAATTCAGTTTTAGCTCTTTGCTCTAGCTCCTCCAATCATATTGCTGTCGAACAAATTGAAAATGAGCTTTTGGATTCCCTGGCACTTAATCTTCAAATACTTCATATGGGCAGCTGCAAGTGTGTTGACGAAACATCTCTTCTAAAGCTTATGTCTCAAATGCAAAAGCTGAAGAGTCTTTGTTTAAGTGATACTCGCCTTTCTGATGGTGCTTTATATAGTTTCAGAGGTTCTTCTTTGGAGATGCTTGATATTTCTAATACTATGGTTTCAAATGCTGCTGTAGCTTATCTTGTCGGTGGAAATCCAGGTTTAAAGTGTCTGAAAGCAAGGGGCTGTAGGAATTTATCTCAACAGGAAAGTGATCCTCAAAAGAGAGAATTTTCTTTCTCCTATTCTTGCAGAGAACTGCATAATGAAATAGGAAGAGCTTGCATGTTGGAAGAAATTGCACTTGGATGGGGATTTTCTTACTCCTCTTTGGAAGCTCTGAAACCTGCAATCACATCACTGAGGAAAATAACTGTGGGCTTAGGTGGATTGTTAGGTGAAGATGGACTGAGAAAACTACCAACTATTTGTCCTATGCTAGAGTTGATTATTCTTTATTTTCAGGTAATATCTGATAGAACCATTATGAACATTATGGCAAACCTGAAGAAGTTGGTAGTTTTGGCTTTCTGTCACTGTCTTGGTGATATATCTATTTTAAGCTTTAAATTTCCTATGCCTAACCTGAGGAAATTAAAGCTTCAGCGGGTGACCCCTTGGATGACCAACAATGATTTGTTTATTCTTACTCAAAGCTGTGCAAATCTTGTTGAGCTTTCGCTGCTAGGGTGTACGCTTCTGGATTCAGAGTCTCAACAAATAATTTCTCAAGGATGGCCAGGCTTGGTGTCTATCCATCTTGAGGAGTGTGGACGAGTAACAACAATGGGGGTTTCTTCTCTTCTCGACTGTAAAGCTCTTGAAGATCTCTTGCTGCGTCATAATTTGGAACATCATATACCTTCCACTACTTATATGTTGATCCATATTATACAGGGTCCTGGGTTGCAGAGAAGCTTTATTTTTGATGCCGCTTCGAAGTTGCCAATGCTTAGGAAAGTGTCGCTAGACTTTTGTGATGCAGCTGAAGGTGATTTTGACATTCCAAATTATGGAGATAGGCATTTCTTAAGTACTCTCAAGATCGCGAAATGCAAGCTTCAGAAGGGCCTCAAGGTTTCATTTGTCAAGGCTCCTAGGAGGCG

>chr7:g1.t1 BTB POZ Domain Containing Protein FBL11

GGTGCACTTTATTTTGGAGTGGAGATGCTTCTCATGAGATGCAAAACTTGGTTTTCTGAGGTAGTATCGGCGGAGGTGCCACCACAAGTACAATTGGATGATTTGATTTCTATTTGGAGTTTTGGTTTAGAGCATGATATTCCATACGACTTATTACTATCTTGCGTGAAGCACATGAATTTGACAGTAGACAGTGAGATGCATCTTTCCAATGCACTTCTAGTTTGGATCGATGCTAACACAGAATGCATGGAAGGCTTGAGCAGAAATGAAGATGTCTGCACTGGCATTTTGAAACAGATCCGTTTAAGCCTTTTGCCGTTGTGGTTTGCTGCAGAGAAAAGAAGTTCTTGTCATTTTTCTAAGTTTGCTGATGAGAGCATTGATTCAATTTTCAGACTACTGAGAATTCCATCCACTGGCTCAGTAGATGCCTTAGGAGCTAGTCACTTGCATGATTTACGCATTCGGCTGACAAAATTTTCTAAGAGAGTAAACCTTTCAAGTTGCTCACAGATAACGTCAGCGGTGCTACTTTTGTCTTTGCTTCCTTCTGCAAACAGTATCGACTACATACTAAGGGGTATTGGGCAGTCACCATTCAACCTTGAACGTCTCGACAGAGATCAATGTTCGGAAGTACTGAATTTGTTGCCAACTTTGTCTTTTGAAGCAGTACAAGAGGTGGATATTTCCAAGTGTCCAAGGTTGCATCTTCAATCTGCCATTGAGTGCTTCCGCAAATCATTTCCATCTTTAAGAATACTGAAGGCAGCTTTTCTTTTGAAATTCAAGATAAGCACTTTGCGCAAACTGGTGCGGAAATGCCCTATGGTCTGTGAAGTTGACTTAACCACCGACACTAGTCCAATTATATCATCACAAGTGTCCGTTGTATCCTCAAGTCCAGCTATAACACCACAAATATCAAATTTGTCCCTAAATGTTCAGGATATGACTTCTTTTTACAACTCTGGACTATCGATAGCAAAACTCACATTGGAGGGCCGAAATGATCTCTATGATTCAGATCTCCAGTATATCTCCAGATTCTGTGTCTCCTTGCAATACGTAAACCTCAAGGGGTGTACTTCATTAACTGATGTTGGCATAGCAAGTCTTTTACGCAGATGTATTAAGCTACATTCTGTTTTAGTTTGTGATACCTCTTTCGGGATAAATTCAGTTTTAGCTCTTTGCTCTAGCTCCTCCAATCATATTGCTGTCGAACAAATTGAAAATGAGCTTTTGGATTCCCTGGCACTTAATCTTCAAATACTTCATATGGGCAGCTGCAAGTGTGTTGACGAAACATCTCTTCTAAAGCTTATGTCTCAAATGCAAAAGCTGAAGAGTCTTTGTTTAAGTGATACTCGCCTTTCTGATGGTGCTTTATATAGTTTCAGAGGTTCTTCTTTGGAGATGCTTGATATTTCTAATACTATGGTTTCAAATGCTGCTGTAGCTTATCTTGTCGGTGGAAATCCAGGTTTAAAGTGTCTGAAAGCAAGGGGCTGTAGGAATTTATCTCAACAGGAAAGTGATCCTCAAAAGAGAGAATTTTCTTTCTCCTATTCTTGCAGAGAACTGCATAATGAAATAGGAAGAACTTGCATGTTGGAAGAAATTGCACTTGGATGGGGATTTTCTTACTCCTCTTTGGAAGCTCTGAAACCTGCAATCACATCACTGAGGAAAATAACTGTGGGCTTAGGTGGATTGTTAGGTGAAGATGGACTGAGAAAACTACCAACTATTTGTCCTATGCTAGAGTTGATTATTCTTTATTTTCAGGTAATATCTGATAGAACCATTATGAACATTATGGCAAACCTGAAGAAGTTGGTAGTTTTGGCTTTCTGTCACTGTCTTGGTGATATATCTATTTTAAGCTTTAAATTTCCTATGCCTAACCTGAGGAAGTTAAAGCTTCAGCGGGTGACCCCTTGGATGACCAACAATGATTTGTTTATTCTTACTCAAAGCTGTGCAAATCTTGTTGAGCTTTCGCTGCTAGGGTGTACGCTTCTGGATTCAGAGTCTCAACAAATAATTTCTCAAGGATGGCCAGGCTTGGTGTCTATCCATCTTGAGGAGTGTGGACGAGTAACAACAATGGGGGTTTCTTCTCTTCTCGACTGTAAAGCTCTTGAAGATCTCTTGCTGCGTCATAATGGTCCTGGGTTGCAGAGAAGCTTTATTTTTGATGCCGCTTCGAAGTTGCCAATGCTTAGGAAAGTGTCGCTAGACTTTTGTGATGCAGCTGAAGGTGATTTTGACATTCCAAATTATGGAGATAGGCATTTCTTAAGTACTCTCAAGATCGCAAAATGCAAGCTTCAGAAGGGCCTCAAGGTTTCATTTGTTAAGGCTCCTTGGAGGCG

>Pp02:g1.t1 Carboxylesterase 9

ATGTCTAAATTCGACCCGTACGCCCACCTCAACATATCCCACGACCCCGCCACCGATACTCTGACCCGCCCCGCCACCACCCCCACGACGCCCACCAACACGGACCCCACCCCGGGGGACCCGGTCGTTTTCAAGGACGTCACGCTCAACCCGGAAACCCGAACCTGGGCCCGAATCTTCCGACCCACTAAACTCCCGTCAAACGACAACACCGTGGCTCGGCTACCCATCATCATCTACTTCCACCACGGCGGCTGGATCCTCCTCTCCGCTGCCGACGCCGCCGCCCACACCAACTGCTCTCAGATCACCTCCGAGGTTCCCGTCATCATCGTCTCCGTCAACTACCGCCTCGCGCCCGAGAGCCGGCTCCCGGCCCAGCACCACGACGCCCTCGACGCGATCCATTGGGTCCGGGCCCAGGCCCAGGACCCGAAGGGCGAGAACTGGATCCGAGACTACGCCGACGTGTCCAGGTGCTACTTGTACGGCTGCGGGTGCGGGGGCAATATCGTCTTTTTCTCCGGGTTGAAAGCGTACCAGCTCCAGCTTGAGCCTCTGAAGATCTCCGGGATTATTATGAACCAGCCCATGTTCGGTGGGCTGCAGAGGACTAATTCGGAGCTGCGATTCGCTACGGACCAGTTGCTTCCGTTGCCTGTGCTGGATCTCATGTGGGACATGGCTTTACCGAAGTCGACGGACAGGAACCATCCGTATTGTAATCCGATGGCGGACGAAGTGCACAGGGATTTGATCAAGCGGCTGGGACGGTGTTTGGTGATTGGGTTTGGTGGGGACCCCATGATCGATCGGCAGCAGGAATTTGTGACGATGCTGGTGGCTTGTGGGGTGAGGGTTGATGCGCGGTTTGATGATCTTGGGTTCCATAACGTTGACTTTGTTGACTCTAGGCGGGCCGCTGCCGTTTTGAACATTGTTAAGGAGTTTATCATCTAA

>chr2:g1.t1 Carboxylesterase 9

ATGTCCAAATTCGACCCGTACACCCACCTCAACATATCCCACGACCCCGCCACCGACACCCTGACCCGCCCCGCCACCACCCCCACGACGCCCACCAACACGGACTCCACCCCGGGGGACCCGGTCGTTTTCAAGGACGTCACGCTCAACCCGGAAACCCGAACCTGGGTCCGAATCTTCCGACCCACTAAACTCCCGTCAAACGACAACACCGTGGCTCGGCTACCCATCATCATCTACTTCCACCACGGCGGCTGGATCCTCCTCTCCGCTGCCGACGCCGCCGCCCACACCAACTGCTCTCAGATCACCTCCGAGGTTCCCGTCATCATCGTCTCCGTCAACTACCGCCTCGCGCCCGAGAGCCGGCTCCCGGCCCAGCACCACGACGCCCTCGACGCAATCCATTGGGTCCGGGCCCAGGCCCAGGACCCGAAGGGCGAGAACTGGATCCGAGACTACGCCGACGTGTCCAGGTGCTACTTGTACGGCTGCGGGTGCGGGGGCAATATCGTCTTTTTCTCCGGGTTGAAAGCGTACCAGCTCCAGCTTGAGCCTCTGAAGATCTCCGGGATTATTATGAACCAGCCCATGTTCGGTGGGCTGCAGAGGACTAATTCGGAGCTGCGATTCGCTACGGACCAGTTGCTTCCGTTACCTGTGCTGGATCTCATGTGGGACATGGCTTTACCGAAGTCGACGGACAGGAACCATCGGTATTGTAATCCGATGGCGGACGAAGTGCACAGGGATTTGATCAAGCGGCTGGGACGGTGTTTGGTGATTGGGTTTGGTGGGGACCCCATGATCGATCGGCAGCAGGAGTTTGTGACGATGCTGGTGGCTTGTGGGGTCAGGGTTGATGCGCGGTTTGATGATCTTGGGTTCCATAACGTTGACTTTGTTGACTCCAGGCGGGCCGCTGCCGTTTTGAACATTGTTAAGGAATTTATCATCTAA

>chr1:g1.t1 Exocyst Complex Component EXO70B1

TGCTTGGTGGGAACGGACTTGATGGAGAAAAACGCAGCGGCGCCTGAAAAGTCCGTCAGTTTTCCAAGACATTCGAGCAAGAAGAACATCCTCTTTTCGAGTCCAATCACACCAAGACGCTACGAAACCATTGATGAAGAAGCTCCACCCGAATCCCAGGACCCGGATCAAGACCCCAACTCCTCCAATCAAACAGCCACCGAATTCAGCCTCTCCGAGGTTCTGGAAGAAGTCGATCGCTTCCTGGGCTCTTTCTCCGAAGCCAAACAAAATGGCAACACCGACCCGACGGAGGAGCTGGAGGTCCCCGACTCCGTCGAGTCGCTATCCAAAACGGTCGAGTCCATGATAAACAAATACGGCCGCAGCCTGGCCAGGTTCGGCGAGAACGAAGACGATGACCAATCTTTCATCAACGCCGTGACCCGCATTTCCAGAATTTCAACAATGTTGGACGATTTCTCTTCCAGTTCCAAAACCGGTTCGGCCTTGAACCGGACCAGCACAGTCCTGCAACGTGCCATGGCCCTATTGGACGAGGAGTTTCGTAACCTCCTCCTCCTCGATCAGCAAGATGCGAATTCTGCCAAATCCGATTATAATCTGGAGCAGAGCTTCTCCAGCAAGATCACCTCCAAGCTATCCTCCTTCAACAGCAACAGCAGCGGCTCCAGTCACGATTCAGTTCGGGGACAGCAACCACAACCCGAACCAGACCGCCTTGATGAGTTTCCCTCTTTCTCCGAGGAGAGCATCTCCACCATGAACAAAATCGCCACCACCATGATCGCCGCCGGCTACGAGAACGAGTGCTGCATGGTCTACAGCATCTCGCGGCGCAACGCCTTCAAATCGGCGCTCAACGATATCGGCTACGAAAGCATCAGCATCGACGAGGTCCAGAGAATGTCGTGGGAGTCCCTCCAGGCGGAGATCGCCACGTGGATCAGCGTCGTCAAGCTCTGCTCCTCCATCCTTTTCTCCCGCGAGCGGAAGCTCTGCGACGCCGTGTTCTCCGGCCACAAATCCCTATCCGAGTCCCTATTCTGCAACCTCGCCCGAGCCGTCGCGATCCAGCTCTTCAACTTCGCCGACGCCGTCGTCCTGACGAAGCGGTCGCCGGAGAAGCTCTTCACGATGCTTGACATGTACGAGACGCTCCGGGATCTGGTCCCGGCGATCCGAGACTCGTACCCGGAGGACATCGGGAATGAGCTGATCTCGGAGGCCGAGGCGGCGAGGAACCGCCTCGGCGAGGTAGCCGTGAGTATCTTATGCGATCTCGAAAACTCGATCAAGAGCGACAACGGAAAAACTCCGGTGCCGAGTGGCGCAGTGCACCCCTTGACGCGCTACGTCATGAACTATCTAAAATACACCTGCGAGTACAAGGACTCGCTAGAGCAAGTGTTCGTGGAATTCGAAAAGACGCACGGGACCCCCGGAACGACGTCGTCCCCGTTCCAAATGCAGTTACTTACGGTTATGGACATGTTGGACAGCAACCTGGACATGAAGTCCAGGCTGTACAGGGACCCGGCGCTGCGCTTCATTTTCTTGATGAACAACGGGCGGTACATCATGCAGAAGGTGAAGGGGTCCACTGAGATCCACCAACTGATGGGCGACACGTGGTGCCGGAAGCGGTCAACGGACCTGAGAGGTTACCACAAGAACTACCAGCGGGAGACGTGGGGGAAGGTGTTGCAGTGCGTGAACCATGAGGGTTTGCAGGTGAGCGGGAAGGTGTCGAAGACGGTGATCAAGGAGAGGTTTAAGTGTTTCAACACGCTGTTCGACGAGATACACAAGACGCAGAGCACGTGGGTGGTGAGCGACGAGCAGCTGCAGTCGGAGCTTAGGGTTTCGGTTTCGGCGGTGGTGATCCCTGCGTACCGGTCCTTCTGGGGGAGGTTCAGGCAGTACTTGGAGGGGACGAAGCAGGCAGAGAAGTATATAAAGTACCAACCGGAGGATATTGAGAATTTGATTGATGACTTGTTCGATGGGAACCCTACTTCAATGATGAGGAGGAGGACGTGA

>Pp01:g1.t1 Exocyst Complex Component EXO70B1

ATGGAGAAAAACGCAGCGGCGCCTGAAAAGTCCGTCAGTTTTCCAAGACATTCGAGCAAGAAGAACATCCTCTTTTCGAGTCCAATCACACCAAGACGCTACGAAACCATTGATGAAGAAGCTCCACCCGAATCCCAAGACCCAGATCAAGACCCCAACTCCTCCAATCAAACAGCCACCGAATTCAGCCTCTCCGAGGTTCTGGAAGAAGTCGATCACTTCCTGGGCTCTTTCTCCGAATCCAAACAAAATGGCAACACCGACCCGACGGAGGAGCTGGAGGTCCCCGACTCCGTCGAGTCGCTATCCAAAACGGTCGAATCCATGATAAACAAATACGGCCGCAGCCTGGCCAGGTTCGGCGAGAACGAAGACGACGACCAATCGTTCATCAACGCCGTGACCCGCATTTCCAGAATTTCAACAATGTTGGACGATTTCTCTTCCAGTTCCAATACCGGTTCGGCCTTGAACCGGACCAGCACAGTCCTGCAACGTGCCATGGCCCTATTGGACGAGGAGTTTCGTAACCTCCTCCTCCTCGATCAGCAAGATGTGAATTCTGCCAAATCGGATTATAATCTGGAGCAGAGCTTCTCCAGCAAGATCACCTCCAAGCTATCCTCCTTCAACAGCAACAGCAGCGGCTCCAGTCACGATTCAGTTCGGGGACAGCAACCACAACCCGAACCAGACCGCCTTGATGAGTTTCCCTCCTTCTCCGAGGAGAGCATCTCCACCATGAACAAAATCGCCACCACCATGATAGCCGCCGGCTACGAGAACGAGTGCTGCATGGTCTACAGCATCTCGCGGCGCAACGCCTTCAAATCGGCGCTCAACGATATCGGCTACGACAGCATCAGCATCGACGAGGTCCAGAGAATGTCGTGGGAGTCCCTCCAGGCAGAGATCGCCACGTGGATCAGCGTCGTCAAGCTCTGCTCCTCCATCCTTTTCTCCCGCGAGCGGAAGCTCTGCGACGCCGTGTTCTCTGGCCACAAATCCCTATCCGAGTCCCTATTCTGCAACCTCGCCCGCGCCGTCGCGATCCAGCTCTTCAACTTCGCCGACGCCGTCGTCCTGACGAAGCGGTCGCCGGAGAAGCTCTTCACGATGCTTGACATGTACGAGACGCTCCGGGATCTGGTCCCGGCGATCCGAGACTCGTACCCGGAGGACATCGGGAATGAGCTGATCTCGGAGGCGGAGGCGGCCAGGAACCGCCTCGGCGAGGTAGCCGTGAGTATCTTATGCGATCTCGAAAACTCGATCAAGAGCGACAACGGAAAAACTCCGGTGCCGAGTGGCGCAGTGCACCCCTTGACGCGCTACGTCATGAACTATCTAAAATACACCTGCGAGTACAAGGACTCGCTAGAGCAAGTGTTCGTGGAATTCGAAAAGACGCACGGGACCCCCGGAACGACGTCGTCCCCGTTCCAAATGCAGTTACTTACGGTTATGGACATGTTGGACAGCAACCTGGACATGAAGTCTAGGCTGTACAGGGACCCGGCGCTGCGCTTCATTTTCTTGATGAACAACGGGCGGTACATCATGCAGAAGGTGAAGGGGTCCACTGAGATCCACCAACTGATGGGCGACACGTGGTGCCGGAAGCGGTCAACGGACCTGAGGGGTTACCACAAGAACTACCAGCGGGAGACGTGGGGGAAGGTGTTGCAGTGCGTGAACCATGAGGGTTTGCAGGTGAGCGGGAAGGTTTCGAAGACGGTGATCAAGGAGAGGTTTAAGTGTTTCAACACGCTGTTCGACGAGATACACAAGACGCAGAGCACGTGGGTGGTGAGCGACGAGCAGCTGCAGTCGGAGCTTAGGGTTTCGGTTTCGGCGGTGGTGATCCCGGCGTACCGGTCCTTCTGGGGGAGGTTCAGGCAGTACTTGGAGGGGACGAAGCAGGCAGAGAAGTATATAAAGTACCAACCGGAGGATATTGAGAATTTGATTGATGACTTGTTCGATGGGAACCCTACTTCAATGATGAGGAGGAGGACGTGA

>chr1:g1.t1 hypothetical protein 1

GAGTGACAGTCTTAAATCCAATTCCTATAAAGCCGAGTTGTCTGTCAATCGAAATGTTTTCATTGTTACTCACAAAGCTGTCAGCATTGTTCCAGGAGATGAATTGAGTGATATCAGACTCCACCACCCAATACAAACAAATCGCTACAACTATAGTTATAGCGGCGGATTCTTGTTTTATGGTTGCTTCGTCCGGCTCAATGGCAAACCAGATAAACATGAGTCGCTTACTCGGTTGCTTACTCGGTTGTGCCGCTATAAACGCATTCTAAGCATCTTCAAGAAGCTCACTTGCCAATCGAATATCTTGGTAAGAAGCTGCACTATTATTCTCCTCTACCTCAATCCAGTCATTGAAGCCCTTGATAGACGAAGGCCAATCAAGCACAAGAACGTCATTGCGAAGCGTGTGGAGGAGGAGAAGATCCAACTTGAGTTCCAACTAGAGTTTGTGGTGGAGCCTGTGGCAAAGCTGAAGATCTAA

>chr1:g2.t1 hypothetical protein 2

ATGTCTGTAGCGAACGCGATAATCGTGGAGGCTATGGGGAAAGTGAAGATTACGCCAGAAGAGGCGTTGGAGCCCAAGATACAACTGGAGTTCATGGAGGAGCCCAAGATTACTCTAGAGTTGAAGATCACGCCAGAGCCCGTAACTAAGCCAAAGATCCAACTGGTGGAGTCGAAGATAGCGCTAGAGCCGGTGGTGCAACCGAAGATCCAATGTGAGTCTGTGGCAGAGTCGAAGATAGTAGAGGCTATGGCGGAGTGGACGATTGTGAAGGCTATAGCGGAGGTGAAGATGGCACTAAAAGCAGCTTTGGATCCTAAGATCGCATTGGTGGCCACGGCGTATCCGAAGATGGCACCGAATGCTGTGCTAAACGCCCTCCTGCCTTCAGCCAAGCTTCAAATCCTTCTATATTGTAAAGGAAAAATATTACAAGAGGCTTTGGCGGAAAAGACGACGGCACTGAAGGTTAGGGACGCTGTGTTAGGGAACGATGATATGGGAGGTTTCAGCGGAGGGGACGAAGACACTAGAGATCAGGGACGCTGTGCTAGAGTCAAATGTTGCGGATTGAGCAATGTAAAGGGAGGCCAAGCTTTAAATCCTTCTATATTGTTAGGGAATGATGATACGAGAGGCTTTGGCAGAGGAGACGACGACACCAGAGGGAAAGATGATATGGGAGGCTTCAAAGGAAGAGACGACAATACTGGAGGGAAAGATGATACGAGAGGCTTCGGCGAAGGAGACGATGACACTGGAGAGGAAGAGACGACAACACTGGAGGTTGGGGATGCTGTGCTAGAGTTAAATGTCGCTGATTGCACCATGGAAGCTTGGGCTGAGGAGGTGGAGGGGGGAAGGGGCACCCTTGCAGCAATACAATCGGCTCATGGCTTCAGCCAAGCTTCAAATCCTTCTATATTGTTAGGAATAGATGATACGGGAGGCTTCAACAGAGGGGACGCGGCACTAGTAGTTTGGGACACTATGCTAGAATCAAATGTCGCGGATTGA

>chr6:g1.t1 hypothetical protein 3

AGCCCACCATGGGTTCTGGGAAAGCTACCGTACACGAGCTGAGCCCGACCACCATCTCCATGGACGTTCGCCATGACCACCTAGAAACCCAACTCCAGGATACCCAAGACACCATCACCACCCTCCTTTCTACCCAAGAAGCTCACCAAACCTACATTTCCTCTCTCCGCAAGTCAGTCGACAACCTCGCGACCGCCCAAACCAACCTTCAGTCCTCCATCAAAAACCAGTTTGGTCAGCTGCAGCAAACCTTTTTCGACGAGCTCCGCCACCTCCGCCCTCACCCACACCTCTCACCTCTCACCTTCCTTCAACACCCACCGCCAAGTCTCCACCTTTAG

>chr6:g2.t1 hypothetical protein 4

ATGTTTTTCATTCTCCTACTTCATTTACCATCGCACCACACTCTGTTTTTTCCCACCCAGACTTCCCTTCTGCCCACCCCAAACTTTTCCCATGGATCTCTGAGGCCACTTAAACTTGATCTCGAACGCTTTTCCGAGGATGACCCTTACGGATGGATCGCTTCTGCGGAGCACTTCCTCAAGTATTATGGGGTGCCTGACGAAGATAAAGTTATTGTCCCTGCTGTTCACCTCTCCGGCGACGCTTCTTTATGGATGTGTTGGTTCGAACAGCGCTTTCCAAAAGATTGGACTTTTTTTACCACATCCTTGCTTCAGTACTTTGGCTCGACAGATATGTGCGATTTTGAAGCCTCCCTCTCTCATGTCCAGCAAACTGGATCTTTAGCTGACTATCTTACCCTCTTTACCAAGCTCGCTTGCAGAGCCTCGGAATGGCCCGACGTACCTCTGCCCGAACCTTTTACCCCCGTACCTCTCCCAACCCCCAACCACACTCCCACAACTCTCCTTCACCTATGCCACCTTTATCTTCACCCTAGCCCTTACCCCCTTCTTCCCGCCACCTCTCCCAAGCCGAAGCACAGAAGCGTCGCTCCAAGGGCCTCTGTTTTACCTGCGACGAGAAATACAGACCTGGTCATCGTTGTCTCAAGCCCATGCTTGCTCTCATTGAGGCCACTGTTCCGGATGATGACTCATCAGTATTTCATGATTGTCACCAGGAGTTGGCCTCTCCAGAGCAGATTTTGGAGCAACATTCAATTTCCTCAACCCAAGCCTTGCTACATGTTTAGGCTTCCCAGTTTATCACTCTGCCCCCCAAACCCTATACACAGCCACATGGGAACAATTACTCATGCAAGGCACCATTCAAAACCTCTCTGTCCAGATTCAAGATTACTCTCTTCTAATTTCAAGTTATTACCCGTTTCTGGTTGTGATATATTATTGGGCGCCGAATGGCTCGAGACATTGGGTCTTATTGAATGGGACTTCAAGAATAAGATTATGCATTTCCACCTTGGCGAACACTCCTATTGCTTGACAGGTATCCATAGCTCTCCCACCACTGCAATTGATGCCAAGCTCATGACTCAAACTCTCTTGGCTGAACCAGAGGGCTTCTTGGCACAGCTTATCCTGTGCTTACCAGACCATGGAGACAATACAACCACTGCACCACCTCTTGCTCTCCACCACCTTTTGCATACCTTCTCTGACTTGTTCAATACCCCTGCCGCATTACCGCCACCTTGCCATATTGACCACCGTATTCCCTTACTTCCGGGTGCTACCCCCGTCAACGTCCGGCCTTATCGATACCCGCATCTCCAAAAATCAGAGATTGAAAGCCTCATCCATGAAATGCTTGCAGTGGGCATCATTCGACCAAGTGCAAGCCCTTACTCCTCACCAGTTCTTTTGGTTAAAAAAAAAGGACGGCTTTTGGCGTCTATGCGTCGATTACCAACTGATCACCTCTTTGTGAAGAAATCCAAGTGTGTTTTTGCCCAACCACAGATCGAGTACCTTGGCCACACCATTTCCTGCCAAGGCATTGCAATGGATCAAACAAAAATTGATTGCATTCAAACATGGTCCAAGCCGTCTTCCCCAAAGTCTCTGAGCGGCTTTCTCGGACTTGCAGGCTACTACCGTAAGTTCGTGAGGAACTTTGGTTTCATTGCTCGCCCACTAACGCAGCTACTCAAAAAAGACAATTTTGTGTGGAACCATGAAGCTGACGCTGCTTTTGCTGCCCTAAAAAATGCATTATCTTCCACGCCAGTGCTTCAGCTGCCCGATTTTTCCAAACAGTTTACAATAGAATGTGATGCCTCTCAAGGAGGACTAGGTGCTGTTCTTTCACAAAATGACCACCCAATTGCCTTACTTTCCAAACCTCTGTCAGGAAGAAATTTGGCACTCTCCGTCTACGAAAAAGAAACGATGTCCGTCATTTTTGCAATTAAAAAATGGCTCCCATACCTTCTTGGCCAACAATTCCGCATCATCACAGACCACCAGACCCTTCGCCACTTCTTGGATCAACGTATTACGACCCTGATCCAACAACGCTGGCTGCTCAAACTAATGGGTTACAACTTTGTGCTCCATTACC

>Pp03:g1.t1 paladin 1

GCTGATTCACTACATGTTCACGGTGTTGCCATTCCAACAGTCGATGGAATCCAGAATGTTCTTAACCATATTGGAGCTCAACAAATTGATGGGAAGCGAACACAAGTTCTTTGGATTAACCTCCGCGAGGAGCCGGTTGGAGACTGGACCAAGTACAATACGACTCTTGGTGCAGTGTCAGGCTAGCAATACCACGAAACTGTAAACTCAGTCGAAGTCGAGTTAATTAAGGGGACAGGCTTATGTTGGTCTATTCTGAAGTTGTGCAGGGCTTTATTAAGATTGAAAAGAATAGGAGAGTTTTTGAGATGCTCTCGGCAAGGAGGTGGAGTGTTTATGGGATATGGTGCACTTCCAGTCTTCTTTGTGGCATCTGTGGTCTATATTAATGGGCGCCCTTTTGTTTTGCGTGACGTGGAGAGGCCCTTTTCCAACCTTGAGTATACGGGAATTAACAGGGCTAGGCTTGAACAAATGGAAGCCCGATTAAAAGAAGATATTTTAATTGAAGCTGCAAGATATGGGAATAAGATCCTTGTCACTGACGAACTACCAGATGGTCAGATGGTGGATCAATGGGAACCAGTATCACGGGATTCTGTGACGACACCGCTAGAGGTTTATGAGGAATTGCAAGTACAAGGATACCTTGTTGACTATGAACGTGTCCCTATAACTGATGAAAAATCACCCAAGGAGCTGGATTTTGATATTTTGGTTCATAAAATTTCTCAAGCTGATATAAATGCAGAAATAATTTTTAACTGCCAAATGGGACGTGGACGAACTACAACAGGAATGGTGATAGCAACTTTGATATACCTCAACCGTATAGGAGCTTCCGGAAACTTGCAGGAATTCACACCCTATTCTCAGGTTTGGAATTCAAAAGCACCTCCAAAGGTAGAAATTCTAGCATGGCAGGTGGCCCTTGGGAAGTTTTCTGGAACATTTGGTTGGAGCGCAATAGTAATCTTTGAGGATTACAAGGTCGCGTGGGTAAATGATTTTTGGGATAGAGTGAGGTTCTGGGCAGCTTTATGGGCATCGGTTTCGGCTGATTTTAAGGATTATGCTCATTCTTCTGTTATGTTGGATTTGTTAGCAGCTGTAAAGTGA

>Pp03:g2.t2 paladin 1

ATGGAGAGGAATAAGAGAATTTTTTATGATGATAAGGGTGACGAGATCGACCAGCTTTGGGACCTAGTTCGTTTTTGGGCATCATTGTGGGCATCAGCTTCTTCAGTATTTAAGGATATTAACTTCAGTTTTCATTCATTTGGATTGGAAAGTGGTATTCCAAGAACCAATTCAATCGGGAAAGTTTCTGACTCCAGTGCAATTGTCACTGACAATTTTCCAAACTCAGAAGATGCGATTCGTAGAGGAGAATATGCAGTCATAAGAAGCTTGATTCGAGTATTAGAGGGTGGTGTTGAAGGCAAAAGACAAGTGGATAAAGTTATTGACAAGTGCGCCTCTATGCAGAACTTACGTGAAGCAATTGCCACTTATCGCAATAGTATTCTGCGCCAACCAGATGAGATGAAAAGGGAGGCGTCACTTTCATTTTTCGTGGAGTATTTGGAACGATACTACTTTCTTATATGCTTTGCCGTGTACATTCATTCAGAGAGAGCAGCCCTCCGCTCTAGTTCCGTTGGTTATAGCAGTTTTGCTGACTGGATGAAAGCTAGGCCAGAACTGTATAGCATTATTCGCAGGTAA

>Pp03:g5.t1 paladin 2

ATGGGTGCACTTGGATATGCAAGTTTGAAACCATCTTTGAAGAAGATTGCTGAATCTGCCGATGGCCGGCCTTATGAGATGGGTGTAGTTGCGGCCTTGAGAAAGGGCGAGGTTCTTGGTAGCCAAACAGTTTTGAAAAGTGACCACTGTCCTGGTTGTCAAAACCAAAATTTACCAGAAGGAGTGGATGGTGCCCCTAATTTTCGGGAGGTCCCTGGATTTCCAGTTTATGGAGTCGCGAATCCAACAATTGATGGTATACGATCTGTCATCCAAAAGATTTGTAGCTCCAAAGATGGTCGACCAGTTTTTTGGCATAATATGAGAGAAGAACCTGTGATTTACATCAATGGAAAACCGTTTGTACTCCGTGAGGTTGAACGACCGTACAAAAATATGCTTGAATACACGGGTATCGATCGTGAGAGAGTAGAGAGGATGGAAGCTCGACTGAAAGAAGATATACTGCGAGAAGCTGAGCATTATGGGGGTGCTATAATGGTTATTCATGAAACAGATGATGGACAAATATTTGATGCTTGGGAACATGTAAATTCTGAGGCTATTCAGACCCCACTTGAGGTTTTTAAAGGTTTGGAGACAGATGGTTTTCCCATAAAGTATGCGCGTGTGCCCATCACTGATGGTAAAGCTCCCAAAAGTTCTGACTTTGACACGTTGGCTATTAATATTGCTTCTGCATCCAAGGACACTGCTTTTGTTTTCAATTGCCAGATGGGCAGAGGAAGGACAACCACAGGTACTGTAATTGCTTGCCTTTTGAAACTTCGAATTGAGCATGGGAGACCTATCAAAATCCTGGTTGACAATATTACCCTTGAAGAGGTGGATGGTGGTAGCTCAAGTGGTGAAGAATCAGGAGGTAATAGTGCTGCATCAACCTCCAGTGTTACAGCTGTAAGAAATGAAAAGGATCAAGGCCGTGTATTTGGCATGAATGACATCCTCTTGTTGTGGAAAATAACAAGATTATTTGATAATGGGGTGGAATGCCGAGAAGCCTTAGATGCTATAATTGATAGATGTTCTGCTCTACAGAACATACGCCAAGCCGTTCTGCAATATAGAAAGGTATTCAATCAACAACATGTTGAGCCAAGGGTAAGGAGGGTGGCATTGAATCGTGGCGCTGAGTACTTGGAGCGCTACTTTCGTTTAATTGCTTTTGCAGCATACTTAGGAAGTGAAGCATTTGATGGATTTTGTGGGCAAGGAGAATCTAGGATGACATTTAAGAATTGGTTGCATCAGAGACCAGAAGTTCAAGCAATGAAATGGAGCATAAGATTAAGGCCTGGACGATTTTTTACTGTCCCTGAGGAGTTGAGAGCACCGCATGAATCTCAACATGGAGATGCTGTCATGGAGGCCATTGTCAAGGCCCGTAGCGGTTCTGTTTTGGGGAAAGGATCTATACTTAAAATGTATTTCTTTCCAGGGGTAAGTGAGTCTGCGATGCAATTGGACAAGGCTGCAAATCAACTGTATAAAATGCATTTAACAGGATTACTTCATTTGGCGATAGAGTTGTCTGGAAGACAGCTGCTTTCTCTTTGA

>Pp07:g1.t1 polyadenylation cleveage factor homolog 4

ATGGATTTGAAGAGCCTATACTGGAACATCTTCTCCTTCCCCTTTCCCTTCTCATTCGGGAAGCATTTTGGTGTTTGGCCTTTGGTTTATCAGTCACTTATTTGTTCAAACAATTTGGCTGTAACTAGTGTTTCGTATCTAATTGTAGAAAGTAATTTGCAGGTTTCTAGTGAGCAGAAGTTACCATCACTTTATCTTTTGGACAGTATTGTAAAGAATATTGGAAGGGATTACATAAAATATTTTGCTGCCAGACTCCCTGAGGTGTTTTGCAAGGCATATAGGCAGGTTGAACCTAATGTACATCAAAGCATGCGCCATCTTTTTGGAACATGGAAGGGAGTATTTCCTGCTCAGACACTTCAGATGATCGAGAAAGAACTTGGGTTTGCTTCAACAGCAAATGGTTCATCCTCTGGAGCTGCAACATCAAGACTAGATTCCCAGTCGCAACGACCGGCACATAGCATTCATGTGAATCCCAAATATTTGGAAAGGCAACGTCTTCAGCAACCAACAAGGACAAAAGGAATGGCCAGTGACTTTTCTGGGGCTATGGCAAACTCAATTGATGATGCAGAGAGGCCGGACAGAGTAGCAAGTCTCAGTGCTGGACGACCGTGGGTTGATCCTACTGTTAAAATGCATAATATGCAGCGTTCTAATACAGATGCACTAAGTGAGCGTGTTCATGAGAAGAACATCGGCGCTGAATATGGAGAATATGAATATGGTTCTGATCTTCCAAGGAGTTCAAACTTAGGAATTGGAAGAATTGGTGGGAAGATTACAGAGCAAGGAAATGATAAACCTTGGTATGGTGGTGGCAGCAGTGTTGCAGAAACAATATCCAGTCAAAGAAATGGTTTTAACATCAAGCATGGACTTACAAATTACTCAGCACCTAAATCTGCAAATGCTGATCCTCGCCTGAAGACAGCACCGGCCATTGCAAGTCGAAGCAGCGGTGTGCTGTCTAATAGCTGGAAGAATTCCGAGGAAGAGGAGTTCAAATGGGATGATATGAACTCGAGGTTAACAGATCATGGTCCCCCCGATATTTCTAGTAACTCAAGAAAAGATTGCTGGACTTCTGATGATTCAGAAAAATTGGGGTTTGGAGGCCACTTTCGTAAACCAAAAGGTGCAAATGACTTTGCAACAACAGTTGATCTGGATACGTCTGCTGATCCTACTGAACATAATGACCTATCTGCTCTTGGGCACCGGATGTCATCACCTTGGCCATTATCGGACTCTCACGGTATGGATGGGCTGACCCCTACTGGCACTCCTGTGATTAGTTCAGTTCATTCAGAGCGTTATGCTTCAAGTTTAAGTGGGCTATCAACAAGTGGAGATTCTTCTGTAGCTAGGCTGGGAAGTCGAGCACAGGTAGCGTCTTCTCGTATTGGAGCCTCAAGTTTCGGGTTTGGTGCAACATCAGGGCCTGCTGTAGCTGTGGGGAAACAGAAGCAGCTTCAGTCTGTGAGAGCCGCATCACCATCTGGACAGTCACTGGTGCACCAGCATTCTCCCGCACCTACATCAACAGTACATCATCCTCATCATCATTTGCAAAGTTTACCTGAACAAGACTATCTTGAGTCCCCGTCCCTTCCTCCTCCTGATTCAAAAGTATCTCAACTTTTGGGAAAGTCAGACTTAGGATTGCATAACCACTATACTGAGGACTCTGCGCCTATCTCAACTCCCAATGTCCGGCTGGGTAGCATAGCAAAATCACGGCCACAAGATTTGCATTCTTCATCTTCGTCAATTAAGAATCCCTCTTTGCCACAGTTGTCTACTTATGTAACTCCCTCAACGGCTGGCATTTCTTTACCAGATCATTCAAATCTCCGTGCTGCAGAAACTTCAGGACAATCAAGCACAAGTAGTTTGTTGGCTGCTGTTATGAAGACCGGAATTCTTTCTGACAAATCAATTACTGGTAGCCTACCCAGTTTGAATCTTCGGGACATGGGACAAAATCAATCACAGTCAGGTGTTCAGCCCCCCTTGCCAAGTGGACCTCCCCCTACCCAGGTTGCACTCCCAGGGTCCAAGGTCGCATCAGCACCTTCATCAAGTCACCTGTCCCATGAGAACTCACCAGCTTCCTCAGATATTTCACTGAAGAAGGTAGGACATCCGCCACTTCCTCCTAGCCAACCCCTCTCTTCTTCCCTTGAGGGTACTGCATCAGCAAATGCTTCAACTGTGGTGAATAATGCCTCAGATCCAATTTCAAACCTTCTAAGCTCTTTAGTTGCAAAGGGTTTGATATCTGCATCAAAGTCGGAGTCACCCACTCCTGTGTCATCCCAAATGCCGAATGAACTGCAAAACCAGAGTGTATCTACACCTGTCACCAGTTCAGTGTCAGTATCTCCAGTTTCAGCTTCTCCAAGTCTTCCTGTTTCATCTCGAACTGATGATGTATCTCTCGCGGAACCTCTTGCTAAAACCTCTGCTGCCTTACCTCAATCCAGCAAGATAGAAACAAAAAATCCCATTGGCATTGAGTTTAAGCCAGATAAAATCCGAGAATTCCATCCATCTGTGATCGAGGAACTTTTTGATGACCTTCCTCATAAGTGCAGCATATGCGGCCTTAGGCTCAAACTTAAAGAACGGCTTGAGAGACACTTAGAGTGGCATGCTTTGAAAACTCCTGAATTCAATGGTTCAGTTAAGGCATCAAGGAGGTGGTATGCAGATTCAACCAATTGGGTTGCTGGAAAGGCAGGACCTCCTTTAGGACCTGAAGACAACATGTCAATAGATAAGCCTAGCGAGACAATGGACAATGGTGAGCCCATGGTTCCAGCCGACGAAAGTCAATGTGTATGTGTTATATGTGGTTATATTTTTGAAGATCTTTACTGTCAAGAAAGGGATGAATGGATGTTCAAAGGAGCTTCATACTTGAGCATTCCATATGGCGTTGGTGATTTGGGAACTACAGAGGAGAGTGTTGTGAAAG

>chr1:g1.t2 putative serine threonine protein kinase

CGTTATCAGCACGAATTCGCTCTCAAAAATTTACCAGCTGAATTCACTGATGATGAACCTCTGCAAACTCCTATCTTCAAAGAAACCCGAACTGGGTTTTCGACTCGTCCTGTTCAGCTCCCTGTAATGGTTAATGAGCTTGAAAAACGAGTCGTCCTCCTCTGCAATTACAGAGCCCAACAGGTACCTTCACCTTCTTCTTATTCTTCCTCTGCCGATATAACATCATCAGATGAAGCCGTCAGGTTCTTAAAGTTAGGCCTTCTTTGTGTGCAAGAGATGGCCAAGCTCCGGCCGCCAATGTCAAGGGCTGTTAAGATTTTGTCTGGTGAGGTAGACATAAAAGATTCTCAGATTTCAGAACCGAGACTTATTTCTGATATCATGGAAATCAAAATGGGGCAACAGCAGTCATGTGATCAGAGCACTTTCTCCAAAAGCCTCCACAGCCAATCATGTGAGCTCCGAGAACTTGAACACCCTCAGATCATTGGCTCGCCTCAGGCTCAGCAACTCCTGGAAGGCGACCGAGTCGGACAGGTCCCTCGAGTCCAAGTCGAACTCAGACCGCCGAGTGGTGTCGTAGCTGCTGGAGGCCACGCTGAAAGCTCAAGGTCCCTGTCCTCTTCCTCTTCTTGCTCAGACGTTTCGCTGAGCTTGCCCCTGCTAAAAGACTCGGATTGCGAATCGGTGGACTCGCCGAGAATGAACTCGTCTGAAAAGTCGTGTTTCAGAGCTTGGAGCTCAAGTCGGCAAACAGAGGGATCGTTAAGGGCTCTGACGACAATGCCACCTGCCTTCTCTTCTGAAGGAAGCTTGCGGACAAAATTGCAGCTGCTGGGTTCATTGTTGTGCTCCCTGACTTCTTCAACGGAGACCCTTTTGATGGGAACGGGAGTGGACAAGACAATCGAAGATGCAAAACCAGGTGTGGTTGAACTTGCAAAGTATGACTTTATCCAAGCTGCTGTTCTGTGTCATCCTTCATTCGTCACTGTGGATGATATCAAAGCTGTTAAAGTTCCCATTTCTGTACTTGGAGCTGAGATTGATCAGCTGTCTCCACCAGAAGTCGTGAAGCAATTTGAAGAGGTTTTATCTGCAAAATTGCTGCTACTTCTGCTGCTCTGGGTGTACAAATCCCAGAACTTGTAG

>chr7:g1.t1 Quirky

ATGGCTGAAAACTCTGATCACCGCAAGCTGATCGTGGAAGTGTGCAACGCCAAGAACTTAATGCCGAAAGACGGCCAGGGAACGGCCAGCGCCTACGCCATCGTCGACTTCGACGGGCAGAGGAGGCGGACCAAGACCAAGCAGAGAGATCTCAATCCCGAGTGGGATGAGAAGCTTGAGTTTCTCGTCCACGACAACGACTCCATGGCCTCCGAGATCCTCGAAATCAATCTCTACAACGACAAGAAGACCGGAAAGCGCAACACTTTTCCTCGGCAAAGTCAAGATCCCCGGAAACACCTTTGCCAAAACTGGCGCCGAGGCCTTGGTTTATTTCCCGTTGGAGAAGCGCAGCGTGTTTTCTCAGATCAAGGGGGAAATCGGATTGAAAATATATTACATCGACGAGGGACCCTCCCGCGGCGGCGGAGGCCCGCGGCGAAAGCAGAGGAAAATAAAAAAGTGGAGGCTAAAACAGAAGAAAAGCCTAAAGAGGAGGAGGCGAAATCAGAGGAGAAGCCCAAGTCACCGGAAGACAGTAAACCGAAAACGGAGGAGGCTTCAGCCCCGCCCCCGGAGGTTGAGAATCCGCCAATCGCATACGGTTCGGAGAAGTCAAAACAAGTGGACAAAGAAAAGCACGTGGAGGCAAATGGCGGCATCAACGAGGTTGAGCTTCTTCAGCCATTGGCCCGGGACCGGAGCCGGAGCGCATACGATCTGGTGGACCGGATGCCGTTTCTTTACGTACGAGTTGTGAAAGCTAAGAGGGGCAATGGCGGCGCTACTGCCGAACCGGTTTACGCCAAGCTCGTGATCGGAATGCACAGCATCAAGACCAAGACCCAGCCAAGCGACAAAGAGTGGGACCAGGTCTTCGCCTTCGACAAGGAAGGCTTAAACTCGACCTCTCTGGAAGTTTCGGTGTGGGCAGAAGCGGAGGAGAAGAAGGAAAACGAGCCTCCTACCAAAACAGAGACTTCTCTCGGAACGGTGTCGTTTGACTTGCAGGAGGTGCCTAAGCGAGTTCCGCCAGACAGCCCCTTGGCTCCGCAATGGTACACTCTGGAGTCGGAGAAGGAAGCTGGGAATGACGTCATGCTCGCCGTGTGGATAGGAACGCAGGCGGACGAGGCGTTTCAGGAGGCGTGGCAGTCGGATTCGGGTGGGTTGATACCCGAGACCCGAGCCAAGGTCTATTTGTCCCCCAAGCTTTGGTATTTGAGACTAACGGTCATCCAAACCCAGGATCTCCAGCTAGCTTCGGGATCCGAACCCAAGCTCAAGGTTCGGAGCCCAGAACTTTATGTCAAAGCTCAACTTGGCGCTCAGCTTTTCAAGACTAGTAGGACCTCCGTGGGCTCCACGTCATCAAGCTCCGCCAACCCCACTTGGAACGAAGACTTGGTTTTTGTAGCAGCTGAGCCGTTCGAGCCGTTTTTGGTTCTAACCGTGGAAGACGTGACTGATTCTCAGACTGTGGGCCATGCGAAGCTCCACGTGCGATTTTCATGCTCATCTTGGCTTAATTTGATCCAAGCGTACACCAGCCCAATACTTCCCAGAATGCACTACGTCCGCCCGCTGGGCCCTGCGCAACAAGACATCCTGAGGCACACGGCCATGCGAATCGTTACGGCTCGGCTCGCGAGGTCGGAGCCGCCGTTGGGGCAAGAGGTGGTTCAGTTCATGTTGGACTCCGACACGCACGTGTGGAGCATGAGGAGAAGCAAGGCCAATTGGTTTCGCGTGGTGGGCTGCCTGTCAAGTGCGGCTATGTTTGCCCGTTGGCTCGACGGAATCCGCACCTGGCAGCATCCACCCACCACTGTTTTGGTGCACGTGCTGCTCGTGGCAGTTGTGCTATGTCCGCATCTGGTGTTCCCCACCGTATTCATGTACGCCTTCTTGATCCTATTGCTGCGATGTCGATATAGGCAGAGGGCCCCGCCCAATATGGACCCAAGGATCTCTTACGTTGATGCGGTGAGCCCGGATGAGCTGGACGAGGAACTTGATGGGTTCCCGTCCACGCGACCGTCGGATGTAATACGCATCCGATACGATCGGCTGCGGGCATTAGGAGGTAGGGCCCAGACTCTGTTAGGTGACGTGGGCAGCGCAAGGCTTTTGTGTTGGTGTCAGGGTTCTATTATTTGCGCCCACCCAAGGTTCCACGACGACATGCCGTCTGTTCCGGTCAACTTTTTCCGGCGACTTCCGTCTCTCTCTGACCAGATTATGTAG

>Pp07:g1.t1 Quirky

ATGGCTGAAAACTCTGATCACCGCAAGCTGATCGTGGAAGTGTGCAACGCCAAGAACTTAATGCCGAAAGACGGCCAGGGAACGGCCAGCGCCTACGCCATCGTCGACTTCGACGGGCAGAGGAGGCGGACCAAGACCAAGCAGAGAGATCTCAATCCCGAGTGGGACGAGAAGCTTGAGTTTCTCGTCCACGACAACGACTCCATGGCCTCCGAGATCCTCGAAATCAATCTCTACAACGACAAGAAGACAGGAAAGCGCAACACTTTTCTCGGCAAAGTCAAGATCCCCGGAAACACCTTTGCCAAAACTGGGGCCGAGGCCTTGGTTTATTTCCCGTTGGAGAAGCGCAGCGTGTTTTCTCAGATCAAGGGGGAAATCGGATTGAAAATATATTACATCGACGAGGACCCTCCCGCGGCGGCGGAGGTGAAGCAGGAGACTGCTCTCCCGCAGGCGGAACAGGAGAAGCCGCTGGAGAATCCGAAAGCGGAAGATCAAAACCCCGAGGCCGCGGCGAAAGCAGAGGAAACTAAAAAAGTGGAGGCTAAAACAGAAGAAAAGCCTAAAGAGGAGGAGGCGAAATCAGAGGAGAAGCCCAAGTCACCGGAAGACAGTAAACCGAAAACGGAGGAGGCTTCAGCCCCGCCCCCGGAGGTTGAGAATCCGCCAATCGCATACGGTTCGGAGAAGTCAAAACAAGTGGAGAAAGAAAAGCACGTGGAGGCAAATGGCGGCATCAACGAGGTTGAGCTTCTTCAGCCATTGGCCCGGGACCGGAGCCTGAGCGCATACGATCTGGTGGACCGGATGCCGTTTCTTTACGTACGAGTTGTGAAAGCTAAGAGGGGCAATGGCGGCTCTACTGCCGAACCGGTTTACGCCAAGCTCGTGATCGGAACGCACAGCATCAAGACCAAGACCCAGCCAAGCGACAAAGAGTGGGACCAGGTCTTCGCCTTCGACAAGGAAGGCTTAAACTCGACCTCTCTGGAAGTTTCGGTGTGGGCAGAAGCGGAGGAGAAGAAGGAAAACGAGCCTCCTACCAGAACAGAGACTTCTCTCGGAACGGTGTCGTTTGACTTGCAGGAGGTGCCTAAGCGAGTTCCGCCAGACAGCCCCTTGGCTCCGCAATGGTACACTCTGGAGTCGGAGAAGGAGGCTGGGAATGACGTCATGCTCGCCGTGTGGATAGGAACGCAGGCGGACGAGGCGTTTCAGGAGGCGTGGCAGTCGGATTCGGGTGGGTTGATACCCGAGACCCGAGCCAAGGTCTATTTGTCTCCCAAGCTTTGGTATTTGAGACTAACGGTCATCCAAACCCAGGATCTCCAGCTAGCTTCGGGATCCGAACCCAAGCTCAAGGTTCGGAGCCCAGAACTTTATGTCAAAGCTCAACTTGGCGCTCAGCTTTTCAAGACCAGTAGGACCTCCGTGGGCTCCACGTCATCAAGCTCCGCCAACCCCACTTGGAACGAAGACTTGGTTTTTGTAGCAGCTGAGCCGTTCGAGCCGTTTTTGGTTCTAACCGTGGAAGACGTGACCAATTCTCAGTCTGTGGGCCATGCGAAGCTCCACGTGCCCAGCGTTGAGAGGAGGAGCGATGATAGGGCTGAACCAAAGTCCAGGTGGCTCAATCTGATTGGCGATGAGAGCCGTCCGTATGCCGGACGAATACATTTGCGAGTTTGTTTGGAAGGTGGGTATCACGTGCTGGATGAGGCGGCGCACGTGACCAGTGATGTTAGAGCCGCGGCTAAGCAGCTAGCCAAGCCTCCTATTGGGTTGCTCGAAGTTGGTATTCGCGGGGCCACAAATTTGCTTCCGGTGAAGACCAAAAACGGTGTGCGTGGGACAACCGATACTTACGTGGTTGCCAAATACGGACCAAAGTGGGTCCGGACCCGAACCATTCTCGACCGATTTAATCCACGCTGGAACGAGCAGTACACATGGGACGTATACGATCCTTGTACGGTCCTCACTATAGGAGTCTTCGACAACGGAAGGTACAAGCGCGACGAAGCTGGAAAACCCGAGAAAGATATTCGGATCGGGAAGATCCGCGTGCGGTTGTCCACTCTCGATACGAATCGGGTGTACAAAAGCTCATATTCCCTTACGGTGTTGCTTCCTGGTGGGGCCAAGAAAATGGGAGAGATTGAGATAGCCGTCCGATTCTCTTGCTCATCTTGGCTTAATTTGATCCAAGCGTACACCAGCCCAATACTTCCCAGAATGCACTACGTCCGCCCGCTGGGCCCTGCGCAACAAGACATCCTGAGGCACACGGCCATGCGAATCGTTACGGCTCGACTCGCTAGGTCGGAGCCGCCGTTGGGGCAAGAGGTGGTTCAGTTCATGTTGGACTCCGACACGCACGTGTGGAGCATGAGGAGAAGCAAGGCCAATTGGTTTCGCGTGGTGGGCTGCCTGTCACGTGGGGCTATGTTTGCCCGTTGGCTCGACGGAATCCGCACCTGGCAGCATCCACCCACCACTGTTTTGGTGCACGTGCTGCTCGTGGCAGTTGTGCTATGTCCGCATCTGGTGTTCCCCACCATATTCATGTACGCCTTCTTGATCCTATTGCTGCGATGTCGATATAGGCAGAGGGCCCCGCCCAATATGGACCCAAGGATCTCTTACGTTGATGCGGTGAGCCCGGATGAGCTGGACGAGGAACTTGATGGGTTCCCGTCCACGCGACCGTCGGATGTAATACGCATCCGATACGATCGGCTGCGGGCATTAGGAGGTAGGGCCCAGACTCTGTTAGGTGACGTGGCAGCGCAAGGGGAGCGGTTGGAGGCGCTTTTTAATTGGAGGGACCCAAGAGCAACGGGCATATTTGTGGTGTTTTGTTTGGTGGCGTCTTTGGTGTTTTACGTGGTGCCTTTCAAGGCTTTTGTGTTGGTGTCAGGGTTCTATTGCTTGCGCCACCCAAGGTTCCACGACGACATGCCGTCTGTTCCGGTCAACTTTTTCCGGCGACTTCCGTCTCTCTCTGACCAGATTATGTAG

>chr7:g1.t2 Shoot gravitropism 6

ATGATTATTCCACAGGCTTATACTGGTGCAGTTGCTATGGTAGGACTTCCTCTGCATCCTCATATTATTTCCCTTTTTTGCATGGAGGTTTGCAGATGCATCTCAGAATTGTGCAGGCATAGATCTAATGGCAATACTATGCTTGCTGAGTGCAAAGCTCGTGCTGATATACCAAATCCCGAGGAGCTTTTTGTTCGCTTGGTGGTGCTTTTGCATGATCCTTTAGCTAGGGAGCAGCTGGCGAGTCAGATTTTGACAGTTCTTTGCTATCTGGCACCTCTCTTTCCAAAAAATATCAACTTGTTTTGGCAAGATGAGATTCCAAAATTAAAGGCATATGTTAGTGACACAGAAGATTTAAGGCAAGATCCTTCTTATCAAGAGACTTGGGACGACATGATAATCAATTTTTTTGCAGAATCTTTGGATGTGATTCAAGATTCTGACTGGGTGATACCCCTTGGAAATGCAATTACCAAACAGTATGGACTTTATACATCTGATGACGAACACTCTGCACTTCTTCATCGCTGCTTTGGTGTGTTTCTTCAAAAAGTGAATGACAGGGCCTATGTCCGTGATAAGATAGATTGGATGTATAAACAAGCTAACATCACAATTCCAACAAATAGGCTTGGTTTGGCAAAAGCCATGGGGCTGGTTGCAGCATCCCACTTGGACACAGTCCTGGAAAAGCTAAAAGGCATTCTGGATAATGTTGAGCAAAGCATTTTTCGAAGAGTATGTTTGCAGATCATTAAGAAATTGCCATCATGATTCTTATCTTTTTTCTCTGATGATTTCAAAACCGAAGAATCTGATGATATACATGCTGCTTTGGCTCTAATGTATGGATATGCTGCGAAATATGCTCCGTCAACCGTAATTGAAGCCAGAATAGATGCACTTGTGGGGACTAATATGCTCTCACGGCTTCTTCATGTGCGCCACCCTACTGCAAAGCAAGCAGTCATTACTGCTATTGATTTACTAGGGAATTTTGTTTCTTTCATGCATGTTTCCATAGAGATTGGGGCCATATGTGTGGCTGATTCTTGTTCATGTGGTGCTTGCATAGTTGATTCCAAGGGTGTAGGTCGTGCTGTCATTAATGCTGCAGAAAATGGTTCATCATTTCCTTTAAAAAGAAGGGACCAAATGCTTGACTATATATTAACTTTAATGGGACGAGATGACAGTGAAAGCTTTTCAGATTCTAGTCTGGAACTTCTGGACACGCAAGCCCGTGCTTTAAGTGCCTGCACTACATTGGTCTCTGTGGAGCCAAAACTAACGATTGAAACCAGAAACCATGTATTAAAGGCCACCTTAGGGTTCTTTGCTTTACCAAATGATCCAATAGATGTCGTCAATCGTCTTATTGACAACCTCATCACTCTCTTGTGTGCGATTCTTCTTACAAGTGGAGAAGATGGGAGAAGTCGAGCAGAGCAGTTATTGCACATCTTGAGACAAATTGATCAGTATGTTTCTTCACCTATGGATTATCAAAGGAGAAGAGGTTGTCTTGCAGTACATGAGATGCTTCTCAAGTTTCGGACAGTTTGCATCACTGCACATTGTGCCCTGGGCTGCCAAGGAAGTTGCACGCACAACAAGCAATTTGACCGTAACTTGCATGGGAACTTTTCCAACTTACCATCGGCTTTTGTATTGCCAAGTCGTGAGGCCTTGTCATTGGGAGACAGGGTCATTATGTATCTTCCACGTTGTGCAGACACTAATTCTGAAGTTAGAACAGTCTCTGCGCAGATTCTTGATCAGCTTTTTAGCATCTCACTTTCACTTCCACGGCCTGAAACTTCCAGTTATGGTGTGGACATAGAATTGTCCTACAGTGCTTTATCTTCCCTTGAGGATGTCATCGCTATTTTGAGGAGTGTAGATCCATCTGAGGTTTTCAGCAGAATTATTTCCTCTGTTTGCATATTACTGACAAAGAACGAGCTTATAGCTACTCTGCATGGTTGCACATCAGCTATATGTGATAAGATCAAGCAATCAGCTGAAGGGGCCATTCAAGCTGTAATTGAGTTTGTCACAAGAAGAGGGAAAGAACTGAGTGAAGCTGATGTTTCAAGGACGACCCAAGCTCTGCTGATGGCTGCCACCCATGTAACTGAGAAGCATTTACGTCAGGAAACTCTTGCTGCTAAGATTTCTTCTCTAGCCGAGAGCACTAGTTCGAAAGTTGTCTTCAATGAAGTTTTGGCCACATCTGGAAGGGATATAGTAACAAAAGATATATCTAGACTACGTGGTGGCTGGCCAATGCAGGATGCATTTTATGCATTTTCCCAGCATACAGTTCTTTCATCTTTGTTCCTGGAGCATGTGATAGGTGTCTTTGGCCAGTATCCCATCCATAAAGGTGATTCAGTAAAAGGAGACAATCCTAGCCATTTGGTTGACGGTCAAATGGAGAATGACATTCTACAAGCTGCTATTATTGCCGTCACTGCATTTTTCAGGGGTGGTGGTAAAATAGGCAAGAAGGCTGTTCAACAAAATTATGCTTCTGTTCTTGCTGAACTCACTCTCCAATTGGGAACTTGTCATGGTCTAGCTAGCTGTGGTCAACATGATCCATTACGGGCTCTTCTAACTGCATTCCAAGCGTTCTGTGAATGTGTTGGAGATCTTGAGATGGGAAAGATTCTGGCTAGAGATGGAGAGCATAATGAAAATGAGAGGTGGATCAATCTTATTGGAGACATAGCGGGCTGCATCTCTATAAAAAGACCAAAAGAGGTTCAAAGCATAAGTGTAATTTTAAGTAAATCATTAAATCGACACCAAAGATACCAAAGGGAAGCTGCAGCTGCTGCATTATCAGAGTTTGTTCGCTACAGTGATGGATTTGGCTCCCTATTGGAGCAGATCGTGGAGGTGTTGTGTCGACATGTATCAGATGAGTCTCCAACAGTTAGGCGCTTGTGTTTGAGAGGACTAGTACAGATACCTTCAATCCATATGTTGCAGTACACAACTCAAGTTCTGGGTGTAATATTAGCTTTACTCGATGATTCTGATGAATCTGTGCAATTAACTGCAGTCTCTTGCTTACTCACGATGCTTGAGGCATCACCCAACGATGCAGTGGAACCCATTTTGCTTAATCTTTCTGTACGGCTTCGAAATCTTCAG

>Pp07:g1.t1 Shoot gravitropism 6

GCTGTTTGGCAATATAGTCTTGATATCCCTTCGCATTCACCTCTGGATGGTGACATCATGTCATTTCTGAATTCTGTTTTTGAGCTTTTGTTGAGAGTTTGGGCGGCTTCACGGGATCTAAAGGTTCGCATATCTTCTGTAGAAGCATTAGGTCAGATGGTTGGCCTTATTACCCGGACACAATTGAAGGCGGCTTTACCCAGACTTGTCCCCACAATATTGGAATTGTATAAAAGAGATCAAGATGTTGCCTTTTTGGCAACATGCAGTCTTCACAATCTCTTACATGCTTCTCTACTGTCAGAAAGTGGTCCTCCTTTACTTGATTTTGAGGAGCTCACAGTCATTCTATCAACACTCCTGCCGGTGGTTTGCATCAATAATGACAACAAGGAGCATTCAGATTTCTCAGTTGGACTGAAGACATATAATGAAGTTCAACGTTGCTTCCTGACAGTTGGTTTGGTATACCCTGAGGATTTATTTGTGTTCCTTATTAATAAATGCAGATTGAAGGAAGAACCTTTGACGTTTGGTGCGCTATGTGTTCTAAAACATCTCTTGCCAAGGCTATCTGAAGCTTGGCACAGCAAACGGCATAATCTCGTTGAAGCTGTACAGTTCCTGCTAGATGATCAAGATTTAGGTGTCCGAAAGGTGCTTTCAGAGTTGATTGTCGTTATGGCTTCACATTGTTACTTAATTGGTTCGTCTGGCGAGCTGTTTGTGGAATATCTTGTGCGTCATTGTGCTCTAACCAATAAGGATAGTAATGACCTTGAGAGGTCCAAGGATGCATCAGGCAACCCCAATATTCCTTTCCAATACAAGAGATTGGAGGTGAAAATTGGGACACTTTGTCCAGCAGAGTTAAGGGCAATCTGTGAAAAAGGCCTTCTTCTGCTGACTATTACGATCCCAGAAATGGAGCATATACTTTGGCCTTTTCTACTGAAGATGATTATTCCACAGGCTTATACTGGTGCAGTTGCTATGGTTTGCAGATGCATCTCAGAATTGTGCAGGCATGGATCTAATAGCAATACTATGCTTGCTGAGTGCAAAGCTCGTGCTGATATACCAAATCCCGAGGAGCTTTTTGTTCGCTTGGTGGTGCTTTTGCATGATCCTTTAGCTAGGGAGCAGCTGGCGAGTCAGATTTTGACAGTTCTTTGCTATCTGGCACCTCTCTTTCCAAAAAATATCAACTTGTTTTGGCAAGATGAGATTCCAAAATTAAAGGCATATGTTAGTGACACAGAAGATTTAAGGCAAGATCCTTCTTATCAAGAGACTTGGGACGACATGATAATCAATTTTTTTGCAGAATCTTTGGATGTGATTCAAGATTCTGACTGGGTGATACCCCTTGGAAATGCAATTACCAAACAGTATGGACTTTATACATCTGATGACGAACACTCTGCACTTCTTCATCGCTGCTTTGGTGTGTTTCTTCAAAAAGTGAATGACAGGGCCTATGTCCGTGATAAGATAGATTGGATGTATAAACAAGCTAACATCACAATTCCAACAAATAGGCTTGGTTTGGCAAAAGCCATGGGGCTGGTTGCAGCATCCCACTTGGACACAGTCCTGGAAAAGCTAAAAGGCATTCTGGATAATGTTGAGCAAAGCATTTTTCGAAGATTCTTATCTTTTTTCTCTGATGATTTCAAAACCGAAGAATCTGATGATATACATGCTGCTTTGGCTCTAATGTATGGATATGCTGCAAAATATGCTCCGTCAACGGTAATTGAAGCCAGAATAGATGCACTTGTGGGGACTAATATGCTCTCACGGCTTCTTCATGTGCGCCACCCTACTGCAAAGCAAGCAGTCATTACTGCTATTGATTTACTAGGGAATTTTGTTTCTTTCATGCATGTTTCCATAGAGATTGGGGCCATATGTGTGGCTGATTCTTGTTCATGTGGTGCTTGCATAGTTGATTCCAAGGGTGTAGGTCGTGCTGTCATTAATGCTGCAGAAAATGGTTCATCATTTCCATTAAAAAGAAGGGACCAAATGCTTGACTATATATTAACTTTAATGGGACGAGATGACAGTGAAAGCTTTTCAGATTCTAGTCTGGAACTTCTGGACACGCAAGCCCGTGCTTTAAGTGCCTGCACTACATTGGTCTCTGTGGAGCCAAAACTAACGATTGAAACCAGAAACCATGTATTAAAGGCCACCTTAGGGTTCTTTGCTTTACCAAATGATCCAATAGATGTCGTCAATCGTCTTATTGACAACCTCATCACTCTCTTGTGTGCGATTCTTCTTACAAGTGGAGAAGATGGGAGAAGTCGAGCAGAGCAGTTATTGCACATCTTGAGACAAATTGATCAGTATGTTTCTTCACCTATGGATTATCAAAGGAGAAGAGGTTGTCTTGCAGTACATGAGATGCTTCTCAAGTTTCGGACAGTTTGCATCACTGCACATTGTGCCCTGGGCTGCCAAGGAAGTTGCACGCACAACAAGCAATTTGACCGTAACTTGCATGGGAACTTTTCCAACTTACCATCGGCTTTTGTATTGCCAAGTCGTGAGGCCTTGTCATTGGGAGACAGGGTCATTATGTATCTTCCACGTTGTGCAGACACTAATTCTGAAGTTAGAACAGTCTCTGCGCAGATTCTTGATCAGCTTTTTAGCATCTCACTTTCACTTCCACGGCCTGAAACTTCCAGTTATGGTGTGGACATAGAATTGTCCTACAGTGCTTTATCGTCCCTTGAGGATGTCATCGCTATTTTGAGGAGTGATGCTTCTATAGATCCATCTGAGGTTTTCAACAGAATTATTTCCTCTGTCTGCATATTACTGACAAAGAATGAGCTTATAGCTACTCTGCATGGTTGCACATCAGCTATATGTGATAAGATCAAGCAATCAGCTGAAGGGGCCATTCAAGCTGTAATTGAGTTTGTCACAAGAAGAGGGAAAGAACTGAGTGAAGCTGATGTTTCAAGGACGACCCAAGCTCTGCTGATGGCTGCCACCCATGTAACTGAGAAGCATTTACGTCAGGAAACTCTTGCTGCTAAGATTTCTTCTCTAGCTGAGAGCACTAGTTCGAAAGTTGTCTTCAATGAAGTTTTGGCCACATCTGGAAGGGATATAGTAACAAAAGATATATCTAGACTACGTGGTGGCTGGCCAATGCAGGATGCATTTTATGCATTTTCCCAGCATACAGTTCTTTCATCTTTGTTTCTGGAGCATGTGATAGGTGTCTTTGGCCAGTATCCCATCCATAAAGGTGATTCAGTAAAAGGAGACAATCCTAGCCATTTGGTTGACGGTCAAATGGAGGATGACATTCTACAAGCTGCTATTATTGCTGTCACTGCATTTTTCAGGGGTGGTGGTAAAATAGGCAAGAAGGCTGTTCAACAAAATTATGCTTCTGTTCTTGCTGAACTCACTCTCCAATTGGGAACTTGTCATGGTCTAGCTAGCTGTGGTCAACATGATCCATTACGGGCTCTTCTAACTGCATTCCAAGCGTTCTGTGAATGTGTTGGAGATCTTGAGATGGGAAAGATTCTGGCTAGAGATGGAGAGCATAATGAAAATGAGAGGTGGATCAATCTTATTGGAGACATAGCGGGCTGCATCTCTATAAAAAGACCAAAAGAGGTTCAAAGCATAAGTGTAATTTTAAGTAAATCATTAAATCGACACCAAAGATACCAAAGGGAAGCTGCAGCTGCTGCATTATCAGAGTTTGTTCGCTACAGCGATGGATTTGGCTCCCTATTGGAGCAGATCGTGGAGGTGTTGTGTCGACATGTATCAGATGAGTCTCCAACAGTTAGGCGCTTGTGTTTGAGAGGACTAGTACAGATACCTTCAATCCATATGTTGCAGTACACAACTCAAGTTCTGGGTGTAATATTAGCTTTACTCGATGATTCTGATGAATCTGTGCAATTAACTGCAGTCTCTTGCTTACTCACGATGCTTGAGGCATCACCCAACGATGCAGTGGAACCCATTTTGCTTAGTCTTTCTGTACGGCTTCGAAATCTTCAGGTATGCATGAACCCAAAGATGCGAGCTAATGCTTTTGCAGCTTTTGGAGCACTAAGCAACTACGGAATTGGGGCACAGCACGAAGCATTTCTTGAGCAGGTACATGCTGCCATCCCACGCTTGGTTCTGCATTTGCATGATGATGATGTTAGTGTGCGACAGGCTTGCCGGAGTACTCTCAAACGAATTGCCCCCTTGCTGGAAATGGAAGGATTGTTGCCCTTGTTCAACATGCATTGCTTCAATCATGATCATCGAACTGACTACGAGGACTTTGTCAGAGACCTTACAAAGCAATTTGCTCAGCATCTTCCCTCTAGAGTTGATACTTACATGGCATCGACAATACAGCATGAGGTAGCCTTAACCCTGCTAAGGGGTGGCAGCACCAAATGCCTATCCAAAAGGAAAGTTAAGGGTTCACTGTTTCTCCTACTCCATACACCTACATCTCATCTTAGATCCTACACCTTTTGGCTTGATAAGATGGTGTGGCAGCTTGCATGGCAAGCCACTGATGGCTCGTTACATGCTGGGGCTTTCGATGCACCATGGCCAATAATTCAGGCAAATGCTATATACTTCTCCAGTTGTATGCTATCTCTGTCAGATGATCAGCACATTTTGACTCTCTATTATGCACAGGTGTTTGGAACATTAGTAGGCAAAATGAGTAAGTCAGCAGATGCGGTGGTGAGAGCAACATGTTCTTCTGCTCTTGGTTTGTTATTAAAATTCTCAAAGTCATCCTCATGGAAAGCAGCTCGAGTTGATCGTGTGGAATCAGGTCGAAGAAGTCATGATTCTTCAGCCTAA

>chr8:g1.t1 Uncharacterized 8_1

ATGAAAAAACAAGTGCCGCTGCAAGTCCGTTTCACTTTCACAAGGATTCTGAAAGTAATGGCGGCTCCTACTCCTACAATTCCAACTTCAAAAGCTCTTATTGTCGGTCCTCCAACACTGCTCACAAGAAGATTAATGGCTTCCAACTCCAACGGTGACATGGCCAAGAAGATCTCCATCTCCCCTATTTCCTCCTCCTCCTCTTCCTACGGTGATCCCTGCCATGATCTGTTTTTCCAGGTGACACGACCAGAAACACGCAGCGATGAGGAGACGACCCAGCAGCAGCAGCAGCAGAACCAGGTGTCCCTTGAATACCTGAAGACACTGCTGCCGCTGGCCTGGTCCCACAATCCCCTAACCACCCTCAAGCTCATCTTCAATCTCCATGCTATTCGTAGCAGCGGAAAATGTTATCCAGAAGGCTTCTACACGGCTGTGTTTTGGCTCCAACAGAAGCACCCCAAGACGCTATTATGCAACCTGCCGTCCATTGCTGATTCGTTCGGTGGTTTGTATGTCCTTATCGAGATTCTCTACCGCCTTCTAGAACAAGACCAATACGCTGCAGAGAGGCTCCACTCTGACCCGGACTATCAGTTGTTACACGACCGGGCAATGGATGTCTTCGTGGAGCGGTTGAAGTCTGATATTGACCAAATGAAGCAGCACAAGCTGGATTTGAAGCCATCAGATTATATAACTGACGGTGATCATGACGACGACGATGAAGATGATAAAGATGGTACTCTTGACGCTGACGCTTATGCTGATCTTTTTGTTAGCGAGGCTGCAGGGTGTTGCGTTACCAAACAACCCCAGGACTCACGCGCTGCCCGCACCATTTTTCTGTGTGAAAGCATTGCGAGGAGGCTTTGCCCACCCAAATCAGACCAACCAAATCAATCTTATGAATCGGAAGAGTGGGAGCGGCTTAGGAATGAGGTTTTGGCGCCCTTGAACAGGTACTGGAAGCGTCAAGACATGTTTATTGGACGACAACGCTCTGAAGTTAAGATGTATTTGGAGAAGGTGAAAAAAGCAGGAGGAAGAGGAGGCAATTTGAGTGGCCATGGCGGCATAATAAAGCCAGATGCTATGCTCCCAAATGAGATCATACGGTATGTAGTAGAAGATGGGAATGTCAGGGAAGGGGCTGAGCTTCAGTGGAAGGCAATGGTGGAGGATATGTCCCTAAAGCAGCAGCAGCAGCAAAAGCAAGGGGAGGGTTTGGGAAAATTTAAAAACTGCTTGGCAGTGTGTCACATAAGCGATTACAATGGCCTAACGCGTTTGGCGGTGAGTTTGGGACTTTTGGTGTCTGAACTGAGTGAAGAGCCGGCATGGAAAGGAAAGGTGATCAGTTCTGGTCATTTGCCGGATCAGCTGATGCTGCATTCGATACAAGGGGATGATCTCAAGTCCAAGTGCGAGTTGATGATGAGGACATGCAACAGAAACTTTGTATCTTTTGCTGATAATTGGCAGATATGGGATTTTATTCTGGAAGTGGCTGCGAAAGAGAACTTGAAGGCAGAGCAGATGGTTAAGAAGGTGTTTGTGTTCGCCGACTACTATGGATATGTTGGGGGTACATCCTGGAAGACTCTGTATGAGGCAAAACGGAGAGAGTTTAAGGAGAAAGGGTACGGGGATGATGCAGTGCCACACATTTTGCACTGGAATATTTCGTACCAGAACATGCCTCGCATAGAAGAACATCATCCAGGGGTGACGCTGTTGAGTGGCGTCTCTGACAATTTGGTCAAGTCCTTCTTGGACAACTATGGGGAAATTGGCCCGCACCATCTAATGGAAGCAGCCATTGCTGATAAAGCGTATCAAGCTCTCATTGTGGTCGACTGA

>Pp08:g1.t1 Uncharacterized 8_1

ATGACAAAACAAGTGCCGCTGCAAGTGTGCTTCACTTTCACAAGGATTCTGAAAGTAATGGCGGCTCCTACTCCTACAATTCCAACTTCAAAAGCTATTGTTGTCGGTCCTCCAACACTGCTCACAAGAAGATTAATGGCTTCCAACTCCAACGGTGACATGGCCAAGAAGATCTCCATCTCCCCTATTTCCTCCTCCTTCTCTTCCTACGGTGATCCCTGCCATGATCTGTTTTTCCAGGTGACACGACCTGAAACACGCAGCGATGAGGAGACGACCCAGCAGCAGCAACAGCAGAACCAGGTGTCCCTTGACTACCTGAAGACACTGCCGCTGGCCTGGTCCCACAATCCCCTAACCACCCTCAAGCTCATCTTCAATCTCCATGCTATTCGTAGCAGCGGAAAATGTTATTCGGAAGGCTTCTACACGGCTGTGTTTTGGCTCCAACAGAAGCACCCCAAGACGCTATTATGCAACCTGCCGTCCATTGCTGATTCGTTCGGTGGTTTGTATGTCCTTATCGAGATTCTCTGCTGCCTTCTAGAACAAGACCAAGACGCTGCAGAGAGGCTCCACTCTGACCCGGACTATCAGTTGTTACACGACCGGGCAATGGATGTCTTCGTGGAGCGGTTGAAGTCTGATATTGACCAAATGAAGCAGCACAAGCTAGATTTGAAGCCATCAGATTATATAACTAACGGTGATGATGACGACGACGATGAAGATGATAAAGATGGTACTCTTGACGCTGACCCTTATGCTGATCTTTTTGTTAGCGAGGCTGCAGGGTGTTGCATTACCAAACAACCCCAGGACTCCTGCGCTGCCCGCACCATTTTTCTGTGTGAAAGCATTGCGAGGAGGCTTTGCCCACCCAAATCAAACCAACCAAATCAATCTTATGAATCCGAAGAGTGGGAGTGGCTTAGGAATGAGGTTTTGGCGCCCTTGAACAAGTACTGGAAGCGTCAAGGCATGTTTATTGGACGACAACGCTCTGAAGTTAAGATGTATTTGGAGGAGGTGAAAAAAGGAGGAAGAGGAGGCAATTTGAGTGGCCATGGCGGAATAATAAAGCCAGATGCTATGCTCCCAAATGAGATCATACGGTATGTAGTAGAAGATGGGGATGTCAGGGAAGGGGCTGAGCTTCAGTGGAAGGCAATGGTGGAGGATATGTACCTAAAGCAGCAGCAGCAGCAAAAGCAGGGGGAGGGTTTGGGAAAATTTAAAAACTGCTTGGCAGTGTGTCACATAAGCGATTACAATGGCCTAACGCGTTTGGCGGTGAGTTTGGGACTTTTGGTGTTTGAACTGAGTGAAGAGCCGGCATGGAAAGGAAAGGTGATCAGTTCTGGTCATTTGCTGGATCAGCTGATGCTGCATTCGATACAAGGGGATGATCTCAAGTGCGAGTTGATGATGAGTACATGCAACAGAAACTTTGTATCTTTTGCTGATAATTGGCAGATATGGGATTTTATTCTGGAAGTGGCTGCGAAAGAGAACTTGAAGGCAGATGAGATGGTTAAGAAGGTGTTTGTGTTCGCCGACTACTATGGATATGTTGGGGGTACATCCTGGAAGACTCTGTATGAGGCAAAACAGAGAGAGTTTAAGGAGAAAGGGTACGAGGATGATGCAGTGTCACACATTTTGCACTGGAATATTTCGTACCAGAACATGCCTCGTATAGAAGAACATCATCCAGGGGTGACGCTGTTGAGTGGCGTCTCTGACAATTTGGTCAAGTCCTTCTTGGACAACTATGGGGAAATTGGCCCGCACCATCTAATGGAAGCAGCCATTGCTGACAAAGCGTATCAAGCTCTCAGTGTGGTCGACTGA

>chr2:g1.t1 Uncharacterized 1

ATGGGTAGAGAGCGAGCTTATGTTGATGACGATATTGATGAAAGTAAGTTCAAGACTTCGGAAGCAATGAACCGCTACAAGAAAATCTTCAGTGTTCAAGCTGTGACGGTTGAGCGGGAGGTCAAATTGAGTGACTTCGAGGATCTTGGGCTTCCCAGAATCTTCAAATCAAGAGGTTGGCTTTTGGCAATGGGTCCTTCAGAGCCTGCTAATATCCAGATTGTCCAAGAATTCTACGCAAACATTCCTCCTTTCTCCGCCGAGCAGAAAACCCCACCTGGGTTGGGCCCTTTTGGTTCTTGTTTGTGGGATGAGATTCTGCATTTCTCTAGCGGTCGAGAATACCCTGACTCATTTGATGTGTATTTGCGAGGTAAGGTGTTAAATTTCTCTGTTTCTGGCATTGCCCAGTTGCTTAAACTTACCAGGCCAAACCCAAATGAAAAATCACCTGGTTTTCCTGGGCTTCTTGTTGATAATCATGACTTGAAGGTGGTGAAATCTACTTTGGGTTGGAACAAGAGGGTCAGGGTTTTGCGTGAAAATCGACTCAGTGATTTGTACAAAGTGCTGAACAGCATAGTGAGATATAATATCGATCCTCCTTGTCACGTTATCCCTTCTTTGCTTAGCCCTGATAGAGCTCGCCTTCTTTATGCCATTGGGAACAATGTACCTATTGATCTGGCCACCTACATTTTTCGTGCCATCTGCCGTGCTGCATTCCCAACTTCCATGCCCGACTCCCTGCCTTTTACTTCCTTGATCACACGCTTCGCCATGGCTTCTCACGTGCCAGTTGAGCCTACGGATAAGCTTTATTCTCCTTGGTCGCCTTTGGACAACGTGGATATTTTTGGCTATAGTTTTACTCCATCTTCTCCTGCAGTCAAGCAACCTCAAGCGGTGGTGAGCCACACGCCCAGTATGGCCATGCTTACCAATGGCGTTGCTGAGCACAGGGGTGATGTGCACCCTGTTTCCACCCACTCGGTCATCCCATCTTCTTCGAAACAGCCATCTGCAGAAATAATGGAGGCTGAAGAAGAAAATAACAGCAATTTGATTCCTAACTCGCATGCTTGTATAAATCCGTTAACTTCAACTTCTGACTCGCATCCAAATAAGAAAGTGAAAAAAGATCCATTGGAACATGCAGCTGGTGAGGTGTCAAAGTTACTGCAGAAGTTTTTGGCAAGTCAAATGAAACTACAACTCAAAGGAGAGGAGGTCCTTGGAGTGGTTTCAAAGATACCTAACCTCAGTAGATTGCAAGTTTTTAAAGCTGTACGCATAATATTGAATGGTAACCCAGAAGAATTCTCTCTACTGAAATCTCTTCCTGATGTTGAGAAGACAAAGTGGATACTCTTACTAATTAGTCAATCTGAAGGAAAAGAAGAAAGTTTGAGATACGCTATCCCTTCTGGCCATG

>chr3:g1.t1 ADP glucose pyrophosphorylase large subunit 1

ATGGATGATGTCACAATCATTGAAAAAATTCTGCGTTCAATGTCACCAAAGTTTAACTATGTTGTTTGTGCAATTGAGGAAGTAAACGACATTGAAACAATGTCATTAGATGAATTGCAAAGTTCACTGTTGGTACATGAGCAGAAATTTAAAAGTCATGACACTTCAAAAGAAGAGAAAGCTTTAAAAGTCTCCACCAGCAACGACTCATCATCATCATGGAGAGGTTCTGGACATGGACGAGAGAAAGAAGAAGAAGCTACACTTTTGCTGGTGTGTCAAGCCGTGGAGAACACTCATCAGGATGTATGGTACATTGACACAGGATGCAGTAACCATATGAGCGGTAACAAGTCATCATTCTCTTACTTAGATGAGTTTTTTCGATCTACTGTTCGTTTTGGTGATAATTCTGCAATTTCTGTTATGGGAAAAGGCAATATTTCAATATGGACTAAGGATAACACTACACAAACCATCTCTAGTGTTTTCTATGTCCCAGATTTAAAAAGCAATTTATTAAGTGTGGGGCAGCTTCAGGAAAAAGGCTATGAGATCACAATTAAAGAAGGAATGTGTCAGATTCAAGATTCAAAGAAGGGACTCATTGCACAGGGATTGAAGACTCTAAAACAGCTGAACATGGCGAGAGGTCTTCCAAACATTGATTGTCCAACTCAAATTTGCGAAGAATGTGTTCTTGGGAAGCAGCATCGTGAAACTTTTCCGAAAGGAAAAGCATGGAGAGCCAAAAATCCTTTGGAGCTTATTTTCTCAGATATTTGTGGGCCCATCAATCCAACTTCCAATGGTGGCAAGAGCCCTGTTGAAAAGGGAGTCGGAAGAAATATGAAAGTTCTTCGAACTGATCGTGGTGGAGAATTCATCTCACAAGAATTTGAGGATTTCTGTGAGACACTAGGAATTCGAAGGCAACTAACGGCACCGTATACACCACAACAGAATGGTATTTGTGAAAGGAAGAACAGAACAGTCTTGAACATGGTTCGCAGCATGCTGAAAGTTCGTGGTGCTCCAAAAACGTTTTGGCCAGAAGCAGTAAATTGGTGTATACACATCTTAAATAGAAGTCCGACTCTTGCTGTCAAAAACATGACGCCGGAAGAAGCTTGGAATGGTCACAAACCAGCTGTAAATTACTTCAAAATTTTCGGTTGTATAGCATATGTTCAAGTTCCCGACGAGAAGAGGAAGAAGCTTGATGATAAAGGAGAAAAATGCATTTTTCTTGGGAAACAATATGGGACAATATGGGACTGGACAGATAAAGGCTCAACGCAGCAGCAGCAGCAGCAGATTTCTGTTGTTTTTGATGAAGAAGCAGCAAGCAAAGAAACTGAAGAGGAATTGCAGCCCTCTAATGACAGCCAGCCATCCAGTTTTCGAACCCAACGCACAAAGAAAAGGCCAGCATGGATGATGGATGAGGTTAGTGATGATGATCAATCCGAAGATGATGGACTGACTTATTTTGCACTGTTTACAGACTCTGATCCTGTAACTTTTCAACAAGCTCACAAAGAAACCAAATGGCAAAGAGCCATGGATGAGGAAATTAGTTCCATTGAGAGAAATAATACATGGGAGTTGACAGATCTTCCTAAAGGGCAGAAGTCGATTGGTGTAAAATGGGTCTATAAAACGAAGCTGAACAAGGAGGGTGGAGTTGACAAATACAAGGCACGGCTGGTGGCTAAAGGATATAAGCAGAAGTATGGCATTGACTATAAAGAAGTGTTCGCTCCGGTCACTAGGATGGATACAATCAGACTGGTACTTTCACTGGCAGCACAAAACTCATGGCCCATCTTTCAATTGGATGTGAAATCAGCGTTCCTACATGGGAACTTACAAGAACAAGTCTATGTCGATCAACCTCCTGGATATGTGCAGCCTGGAAAGGAAGGGAAGGTATACAAGTTGAGAAAAGCATTATACGGGCTGAAACAAGCACCTAGAGCTTGGTACAGCCGTATAGATGCTTATTTTGCTAAAGAAGGTTTTCAGAAGTGTCCGTATGAACACACGTTGTTTACTAAAATTGGTGCTGATGGAAAAATTCTTATTGTCTGTTTGTATGTAGATGATTTAATCTATACAGGCAATGACATGACTATGTTTGATGTCTTTAAGAAATCCATGATGGCAGAATTTGAAATGTCTGATCTTGGTTTGATGCACTACTTCCTTGGCATCGAAGTAGAGCAATCTCCTGCTGGGAATTTCATATCCCAAAAGAAGTATGTTCATACTATTTTGGACAGGTTTCAGATGTTGAATTGCAATCCTGTTGGCACTCCTACTGAATGTGGTTTGAAGCTAACAAAGGATCCTGAAGGAGAGAAAGTAAATAGCACCCTCTTCAAACAAATTGATCGATCCATTTTGGAGACTATTCTCCAGAAGGACACTTCCAAGCAGATATGGGAGTCTATGAAGAAAAAGTATAAGAGTTTCAAAATCTCTCCGGAGTGCTTGAAGCTGTGCACGCTTCACCTTGGTGGAACCTTGATACTTTTTCTTCATAGACTCCCATATCTGCTTGGAAGTGTCCTTCTGGAGAATAGTCTCCAAAATGGATCGATCAATTGCTTGGAAGAGGTAGTTTTTCACTTTCAGGTCCTTCAATTTCTGTTCATCAAGTCTTTTCTGTTGCGCTTCTGTCAACAACGCTCCCTCTGCTGGTTCAGCAATTCCATTTTCAATGAGACTCCAATACTCTTTGGATCTCAGAAAATTCTCCATCAACATGCCCCAATGGTCATAATGACCATCGAAGCGAGGTATGGCCGGCTGCACGTAGTTCTCTTGAACCTTGTCGGATGCCATCAATTCTTGCTGCTGCAAGTTAAGAAAGCTGCTGCTTTTTGGAGAATTTTAAGTCAGGCCCAGTGGGGGCTCTGA

>chr2:g1.t1 alpha-ketoglutarate-dependent dioxygenase alkB homolog 6-like

ATGGGTAGAGAGCGAGCTTATGTTGATGACGATATTGATGAAAGTAAGTTCAAGACTTCGGAAGCAATGAACCGCTACAAGAAAATCTTCAGTGTTCAAGCTGTGACGGTTGAGCGGGAGGTCAAATTGAGTGACTTCGAGGATCTTGGGCTTCCCAGAATCTTCAAATCAAGAGGTTGGCTTTTGGCAATGGGTCCTTCAGAGCCTGCTAATATCCAGATTGTCCAAGAATTCTACGCAAACATTCCTCCTTTCTCCGCCGAGCAGAAAACCCCACCTGGGTTGGGCCCTTTTGGTTCTTGTTTGTGGGATGAGATTCTGCATTTCTCTAGCGGTCGAGAATACCCTGACTCATTTGATGTGTATTTGCGAGGTAAGGTGTTAAATTTCTCTGTTTCTGGCATTGCCCAGTTGCTTAAACTTACCAGGCCAAACCCAAATGAAAAATCACCTGGTTTTCCTGGGCTTCTTGTTGATAATCATGACTTGAAGGTGGTGAAATCTACTTTGGGTTGGAACAAGAGGGTCAGGGTTTTGCGTGAAAATCGACTCAGTGATTTGTACAAAGTGCTGAACAGCATAGTGAGATATAATATCGATCCTCCTTGTCACGTTATCCCTTCTTTGCTTAGCCCTGATAGAGCTCGCCTTCTTTATGCCATTGGGAACAATGTACCTATTGATCTGGCCACCTACATTTTTCGTGCCATCTGCCGTGCTGCATTCCCAACTTCCATGCCCGACTCCCTGCCTTTTACTTCCTTGATCACACGCTTCGCCATGGCTTCTCACGTGCCAGTTGAGCCTACGGATAAGCTTTATTCTCCTTGGTCGCCTTTGGACAACGTGGATATTTTTGGCTATAGTTTTACTCCATCTTCTCCTGCAGTCAAGCAACCTCAAGCGGTGGTGAGCCACACGCCCAGTATGGCCATGCTTACCAATGGCGTTGCTGAGCACAGGGGTGATGTGCACCCTGTTTCCACCCACTCGGTCATCCCATCTTCTTCGAAACAGCCATCTGCAGAAATAATGGAGGCTGAAGAAGAAAATAACAGCAATTTGATTCCTAACTCGCATGCTTGTATAAATCCGTTAACTTCAACTTCTGACTCGCATCCAAATAAGAAAGTGAAAAAAGATCCATTGGAACATGCAGCTGGTGAGGTGTCAAAGTTACTGCAGAAGTTTTTGGCAAGTCAAATGAAACTACAACTCAAAGGAGAGGAGGTCCTTGGAGTGGTTTCAAAGATACCTAACCTCAGTAGATTGCAAGTTTTTAAAGCTGTACGCATAATATTGAATGGTAACCCAGAAGAATTCTCTCTACTGAAATCTCTTCCTGATGTTGAGAAGACAAAGTGGATACTCTTACTAATTAGTCAATCTGAAGGAAAAGAAGAAAGTTTGAGATACGCTATCCCTTCTGGCCATGCTATCATTCTCATTTATATACTTGTTCTTGTCCTTATTCTTATTCACACTCACACTTCCAAGCACTACTGCATAACGTCTTATGTTGGTTACAATTATCTTCTCGACTTGTTAGCTATGAATTACATGCTTCAAGAACATAACTTATAA

>chr8:g1.t1 At3g47530

GAAGGGTTAGGGATGAAAAAACAAGTGCCGCTGCAAGTCCGTTTCACTTTCACAAGGATTCTGAAAGTAATGGCGGCTCCTACTCCTACAATTCCAACTTCAAAAGCTCTTATTGTCGGTCCTCCAACACTGCTCACAAGAAGATTAATGGCTTCCAACTCCAACGGTGACATGGCCAAGAAGATCTCCATCTCCCCTATTTCCTCCTCCTCCTCTTCCTACGGTGATCCCTGCCATGATCTGTTTTTCCAGGTGACACGACCAGAAACACGCAGCGATGAGGAGACGACCCAGCAGCAGCAGCAGCAGAACCAGGTGTCCCTTGAATACCTGAAGACACTGCTGCCGCTGGCCTGGTCCCACAATCCCCTAACCACCCTCAAGCTCATCTTCAATCTCCATGCTATTCGTAGCAGCGGAAAATGTTATCCAGAAGGCTTCTACACGGCTGTGTTTTGGCTCCAACAGAAGCACCCCAAGACGCTATTATGCAACCTGCCGTCCATTGCTGATTCGTTCGGTGGTTTGTATGTCCTTATCGAGATTCTCTACCGCCTTCTAGAACAAGACCAATACGCTGCAGAGAGGCTCCACTCTGACCCGGACTATCAGTTGTTACACGACCGGGCAATGGATGTCTTCGTGGAGCGGTTGAAGTCTGATATTGACCAAATGAAGCAGCACAAGCTGGATTTGAAGCCATCAGATTATATAACTGACGGTGATCATGACGACGACGATGAAGATGATAAAGATGGTACTCTTGACGCTGACGCTTATGCTGATCTTTTTGTTAGCGAGGCTGCAGGGTGTTGCGTTACCAAACAACCCCAGGACTCACGCGCTGCCCGCACCATTTTTCTGTGTGAAAGCATTGCGAGGAGGCTTTGCCCACCCAAATCAGACCAACCAAATCAATCTTATGAATCGGAAGAGTGGGAGCGGCTTAGGAATGAGGTTTTGGCGCCCTTGAACAGGTACTGGAAGCGTCAAGACATGTTTATTGGACGACAACGCTCTGAAGTTAAGATGTATTTGGAGAAGGTGAAAAAAGCAGGAGGAAGAGGAGGCAATTTGAGTGGCCATGGCGGCATAATAAAGCCAGATGCTATGCTCCCAAATGAGATCATACGGTATGTAGTAGAAGATGGGAATGTCAGGGAAGGGGCTGAGCTTCAGTGGAAGGCAATGGTGGAGGATATGTCCCTAAAGCAGCAGCAGCAGCAAAAGCAAGGGGAGGGTTTGGGAAAATTTAAAAACTGCTTGGCAGTGTGTCACATAAGCGATTACAATGGCCTAACGCGTTTGGCGGTGAGTTTGGGACTTTTGGTGTCTGAACTGAGTGAAGAGCCGGCATGGAAAGGAAAGGTGATCAGTTCTGGTCATTTGCCGGATCAGCTGATGCTGCATTCGATACAAGGGGATGATCTCAAGTCCAAGTGCGAGTTGATGATGAGGACATGCAACAGAAACTTTGTATCTTTTGCTGATAATTGGCAGATATGGGATTTTATTCTGGAAGTGGCTGCGAAAGAGAACTTGAAGGCAGAGCAGATGGTTAAGAAGGTGTTTGTGTTCGCCGACTACTATGGATATGTTGGGGGTACATCCTGGAAGACTCTGTATGAGGCAAAACGGAGAGAGTTTAAGGAGAAAGGGTACGGGGATGATGCAGTGCCACACATTTTGCACTGGAATATTTCGTACCAGAACATGCCTCGCATAGAAGAACATCATCCAGGGGTGACGCTGTTGAGTGGCGTCTCTGACAATTTGGTCAAGTCCTTCTTGGACAACTATGGGGAAATTGGCCCGCACCATCTAATGGAAGCAGCCATTGCTGATAAAGCGTATCAAGCTCTCATTGTGGTCGACTGA

>Pp08:g1.t2 ATP phosphoribosyl transferase 1

GGGAATGAATATCTTATCATTGTTCATGATGCTCTTGATTATGGGGTTGCTGCTTATCCCTTACAAAATTCACTGAAGGATTTAGCACAAATGCCTCAATGGGCTGAAGAGAAACCTCTGCGAGTTGCTACTGGCTTCACCTATCTGGGTCCGAAATTTATGAAAGAAAATGGACTGAACCATGTGAGCTTTTCAACTGCCGATGAAGCGCTCGAGGCAGCTCCCACAGTAGTTCTTGTTGCAAGCAAGAGATCGTTGATCCAAAGGAAAAGTGCACTGGACACGACACATGAGATTCTTGAAATATTGGAGGCGCATCTAAGGGCTGAGGGTGAGTTCACGGTAACTGCAAATATGAGAGGAAGTAGGGCAGAGGAAGTGGCTGAGCGTGTTTTGAGCCAGCCATCATTATCACGTTTGCAGGGACCCACCATAAGTCTAGTTTTTTGCAAACGTGATGGGCAAATTGCTTCTGATTACTATGCCATAGTCATATGTGTACCCAAAAAGGCGCTCTACAGGTCTGTACAACAACTGAGAGTGATTGGAGGCAGCAGGGTTCTAGTTTCCTCTTTGACCTACATTTTTTATGAAGAAACTCTGAGATGGCGCGAACTCCTCTCAGCACTTGGCCTCCAGTTCTCTCTCTGTGGTTGA

>Pp03:g1.t1 auxin transport BIG

AGTAATACGGTGCAGCTTTTGGAGATAGCGTTGGTTGATGAAATGGATAAGGCACCTGACACACTGCAACCTTGCTCTGTTGATAGTCTGGTGGACTTGTTACCATCTGTTACCAGTAGTTCTTGTGGTAATGAGTTTGATAACCACACTAAATTTGGTCCACAAGGTGTCAATTGCTCAGGGTCAGAGATACCGCTAGATCGTCTGGATATGTCTTTAGCTTATGAGTGCATACAATCTGATCGGCAAACCAATATGTTTGATGAGAAGATGGTTGGGACAAACTTCTTGAAGAGACTGTCCTTCAGTTTGAGGATTTATAAAGCTACTTCGAAGTCTCTCAAAGGAAATCTAGATATTGAATATGAAGCCTCATTGGTGCAAGCAGTTGGTTCTTTTGCTGATGTAGTGCCTGGGTTGTTTAGACCTGTATTGGAGTTTGTAAATATCAATGTGGCAATCGAGGGTAGTTTTGAGACCCCATTCTGTTGCTACTGGAAGACTTTCTTGAACTCATTTGGGATTAGTCAGGCTGTCATTTGGAAGGAGTTAGACACAGAACTTGATGGCAATAGCGTCAATATTCTTGATTCCCCTAGATGCCTTGTTCATTCTGAGAAATTCCTTTACTTCACAGGCACACATTTGAACATCGACTCTGAATTAGAAATATTGCGTGCCATGAAGATGAAGCCTTTTTTGGAGACCTTCTCTATGAAGGTGGTTGTGGAACTACAAATGGATACGATCAGCCAGCAATTGTTGCTAACTCTAACTCTAGCTCCATCTGGTTTTCATATTGAGATTCTTCTCATGTCATTTCACTTATCCTCTGAAGAGGAAAAAGCATTACATGCAAATGCGTCGCCATATGAAATTTTACCCACGGACTTGTCCTTCGTCTTTGCTTGTACACCTACGATCCAAGTTGAGGGACACTCCGTACTCCACTTCACAGCTCGGGGTGAACGATCATTTGTCATCTTGAGTCTCAATTGTATTTCAAAATATTATGCTCGAGTTGGGATGACATCTGTTCAACCATGTCAGGCTGTAATTGTGGAGGATCTTGTTATTGAAAGATACATTTTCGTACCTTGTTGGGACTTTCCAACTATTGGCACTGCAAAGGACAATCAGCTTCCTTTAGGGAGCTATCTGCAGACTCTTGGCACTTCTGAAATAGCAAATATCTTTTACTTCAGCCATTCAATTCAAGGTCGTCGTGGTGTTGATGTGAAAAATAATTTTCTTGAAGACATTGTTCATCTGCTTCATGTATCCAATTGTAGTTATTTGGACGAGACTTCAAATAGAAATGCCATGTAA

>chr4:g1.t2 Detoxification 45

CAGCTGAAAAAACAAAATGGTGGGGTGAATGAAGCTGGGGAAGGACATGAGGACCTCGGTAGTCCTCATATGAATGAAGGAGCAGCCGATGACTTCTCGCCTCCACATGAGTACGTTAGTCCGCCTACAGAACCGGCAGTAATGGAAACACAAGTCCCCGCTGATGGAGCCGAACCTTCCGCAGCCATGGTAGTCGAAGAGGTAGCAATGACTGCTTCAGTTGCTGACTTAGAAGTGCATGAAGCAGCTGAGCAGGCACAATCCGAGAAGCTGCCTACCCCGGAAGATGTGGCCGGGTGTGACGAAATCTGCCAAAGAGTGTTGTTTTTGTTGGATGATTGGAAAAAGACTCAGAGTATGGCATCGGGAGGTCCAATGAATCCTCCAAGTCTACCCACAGTTAGTATGGCTGGTGATGAAGAAGGGAGTTCGAGTGTTGAAAAAAAGGAAGTGGAAGGTAAAGGGTGCAGACAGAAGCGCCCGGTGCAGACATTGTTGAGCCCATTTACTGATCCTTTGAGGAAGAAGAGGACGATGAGTGTGTCGGCCGCGATTGCAACCCCGCCATGTTTTGATCCAACAAAACCCGTGCCCATTGAAGATGTGAAGGCAGTAATAGAGTTTTGCACTGCCTGGAAAAACGATATCAGTGCGGAGGTGCAGCTGGAATCATTTTCAGTAGGCGCAGATTTTTTCTACAAACTTATCGATGACACGGAATGGGTTAGCTCAAGGCACCTGGACATGGCAACCTTTCTTATCCGGAAAAGGCAACTCTCTCATCCGTTGGTATTTGGAACTGACTGGACAACGGCAGATTATTGCTTGCAGCAATTTCTAGAGCCGTTGAAACCGACTGCGAAGAAACGTAGAGCGAAGAAGGCAGCTGCTTCAAACACCGTTGACCTTCCACCTAACAAGCTCAAGAATATACATCACTATGTGCGCGGTACGTGGCAGCACGGGTATGGCCAAGCTTGGACAAAAGTCCGGAAGGTCTATTTTCCATATAATCTTCGAGGGTCCCACTGGGTTGCAGTCGAAATTGATTTCGTCAGACATACTGCAACTGTGTATGACTCCTATGTTGCGTATACCAAATCTTCGAGGCTGGTTACACTGCTGCACCCTATTAGAGATACGCTAGCACGAGTGCTGTACGAGATGCACTTTTATGAAGATTCTGAGGTTGAAGAGGTTAAGCAAAAGGGGCTGCAGATGTCGAGGTTTACGCCATTCTCAGTTTGCAACATTGCAGGTGTTCCACAACAATTAGATGGTACGTCTTGCGGAATCATGACCGTCAAATTCATCGAGCATCTTAGTGCTGGGATTTCGGTGGATAAAGTTGACCCTTTGAAGATCAAATATTACCGACTGAAGCTTGCAATTGAGGGAACTCCTTCATATGAACATGCAGACGTAGATGCTTTGGTGGGGTCCCTCCCTTCCTTTGTCCTTCTTGATCTTACAAGGGCAAGCAAGGTGATGGGGGTTACAAGATTTGATACCTATCTTAAACATTGTGAAGCTGATATGAGAAACATTGCTAACAGATTCTTGGTCCTGTCTATTTCAGCCATTGATCCCTTAGCACAGCTAATGGAGACTGCTTACATTGGTAGATTGGGTTCTGTGGAGTTGGCTTCAGCTGGTATTTCAATGAACATCTTCAATTATATATCAAAGCTATTTAATATCCCTCTCCTCAGTGTTGCTACATCTTTTGTTGCTGAAGATCTTGCAAAGAGTGAAAGTATAGCTTCTACTTCAGAAAATGGTTGGCTAGGAGACATTACTAATGGTAAACCCGAACGTACTGATGGAGTAACTGAGAGAAAGCAGCTATCTTCAGTGTCTACAGCTTTACTATTATCAGTGGGGATTGGGATTTTTGAGGCCGTAGCCTTGTCTTTGGGATCTGGATTGTTTCTGAATATGATGGCCATATCAATGGACTCACCTATGCGCATTCCTGCAGAACGGTTTCTTTCACTAAGACAATTTTCTTACAACCACACATGTATTGGTAATCTCCTAGCTGCATTTTTATTACCCATCCTTATGTTTTATTTTTGTTTGGGTGTAACTGTTGCAGCCCTTTCCACTACTATATCTCAATACACTGTCACCTTTTTAATGATCTGGTTTTTAAATAAGAGAGCTATACTATTGCCTCCAAAGTTAAACCTCGAGATAATTCTCTCTTGTCTTCCTTTTCGAAGTTCTTTCCCTAATTGCTTTTGTTTTCAACTCTGGGATGATACAGGTGGTTTTCTTCTTGGAACAACTCTTGCTGTTCTTACTACCTTGACATTGGGAACATCAATGGCTGCTCGTCAAGCTCCAGTAGCTATGGCTGCTCATCAGATATGTATACAAGTTTGGTTGGCTGTATCCCTTCTAACTGATGCAATGGCCGCATCTGGTCAGGCCCTGATTGCTACTTATTTATGTAAAGGTGAATACAAAATTGTGAAAGAAGTTGCTGACTCCGTGTTAAAGGTATAG

>Pp03:g1.t1 DNA-directed RNA polymerase III

AGTTCATCTCTCTGCGGTTTCGATATTCAAAGAAGACGATGAGTTCAGACGAAGCCAGAACCCTAGTCTCCACCAGACAGCCCTTCATCGAAGTCGAACAGGATCGCAGGGACTGCCCGGGGCATTTTGGGTATTTGGCACTTGCCCTCCTTGTTTACAATGTTGGGTATATGGGCACTGTCGTGGATATTCTCAAGTGCATCTGCAAGTCATGCTCTGGTCTGCTTTTGAAGCAAGAAATGCGGGTTGAGCTTTTGAGGAGGATGAGAAATCCCAAGCTGGGTCCTTTGAAGAAAACTGAGTTAATGAAAATGGTGGTGAAGAAGTGTAATGGTTTGGCTGCTAACAATAGGCCAGTTGAGTGCTCCAAATGTGGATATTTGAATGGTTCTGTGAAGAAGGCTACCGGCATGGTTGCTATTATTCATGATCGTTCTAAATTTAGTGGTGTTATGGATGAGTTGAGGTCAACAATTTCTGAAACTAAAGTTTCCAGTGCACCATTTAGCTTGGAGACTCATTTGATGAACCCTGGAGAAGTTTTCTCTCTGTTTAAACAGATGCTTGATGAGGATTGTGAGTTACTTTATCTTTGTACCAGGCCAGAGAACCTATTGATCATGAACATTCCCGTGCCTCCTACTGCGATTCGTCCTTCAGTTTTGGTGGACGACTCACGGACAAATGAAAATGACATTACAGAGAGGTTAAAGAACATTGTTCAAGCAAATGCTCGTCTTCTACACGATTTAACACAAGATTTACCACCAGCTTATGCAGGCGGCATATATGGCAATCCGTTGGGTGGTTTTATGCAGCGGATCAAAGGGAAGCAGGGGCGTTTTCGTGGGAATTTATCTGCCAAGCGGGTTGAATATACTGGCAGAAGTGTTGTATCACCTGACCCTAATTTGAAAATTAATGAGGTTGGGCTCCCTATCCAAATGGCTCAAATCTTAACCTACCCTGAACGGGTTTCTCGTTATAATATTGAGAAGTTGAGGAAGTGTGTCAGTAATGGAATTTATACGTACCCTGGTGCCAGATACGTACGCCTTGAACGTCATCTAGAAGACGGGGATTTTCTTCTTTTCAACAGACAACCAAACCTGCATAGGATGTCCGTCATGTGTCACAGGGCTAAGGTTATGTCTTGGAGAACATTGAGGTTTAATGAATCTGTTTGCAACCCTTACAATGCTGATTTTGATGGAAAGCAGTTATTTAGTGTTCTTGTACGCCCGAATGCAAATGTGAGAGTCTATCTAAATCTGACTGTTAAGGAGAAGTCCTACTCCAAGACTGATGAAGATGGAAGGGCAATTGAGGTAATGTGCCCAAATGATGGGTTTGTCTATTTTCGTAACAGGGAGCTTATAGCCGGGCAACTTGGGAAGGGTACTTTAGGAAATGGCAATAAGGGTGGGTTTTTCTCCGTTCTTCTCAGGGACTATAAAGCTCATGCTGCTGCTGCTTGCATGAATCGTGTAACTAAGTTAAATGGGCGATGGATTGCACAATCACTTGAGTCTGAGATAACTTGTGCATTAAACAACATCAGAGAGCAAACTGGAAAGTTGTGCATGCAAAAGCTACATTGGAGAAATAGCCCCTTGATCATGTCCCAGTGTGGTTCTAAAGGATCTGCTATCAATATCAGCCAGATGGTTGCCTGTGTTGGTCAGCAATCGGTTGGTGGTTGTCGTGCACCTAATGGATTCATAGACCGAAGCCTTCCTCACTTCCCTAGAGATGCCAAAACCCTTGCAGCTAAAGGCTTTGTTGCTAGTTCCTTTTACAGTGGCTTATTGACCAAAGTTATGGAGGACCTATGTGTTCAGTATGATAACACAGTACGGAATTCAAGTGGATGTGTAATTCAGTTTTGTTATGGAGATGATGGTATGGATCCTGCAGTAATGGAAGGAACAGAAGATGGAGCTCCATTAGACTTACCCCGATTGTTTCTGAAAGCCAAGGCGACATGTCCTGCTAGAGAAAATGAATACTTGTCTCCTGAACAAGTGAATGAGATGGTGAGAAGCAGGCTTTCAAAACAAGATATGACTCTTGATGGTGGTTGCTTCGTTGGTTTCAAAACTTCTTTGGAGCAATTCCTGAATAAATATGTTGAAGCATTCAGAAAAACACACGAGACGTTTTTGTTGGATGATCATAGTGCCTTGAAGCAGAAGATTGTGCAAAACATATCTGGTGTTACTTTTAGACAGCTGGAAATGACGCTGAAAACTTTCCACTTTGCTGGAGTTGCCAGCATGAACATTACACTTGGTGTCCCTCGTATCAAGGAAATTTTAGACGCAGTGAAAAAGATCCGTACACCTGTCATCTTTGCAACACTTGAGTGTGAAACGAATGTGAAATTTGCAAGATCAGCATCTATAGTTGTCACGCTTGATATGGCCATGATTCAAGATGCACACTTGTCTATAGATGCTAATGTGGTAAAAGAATCAATTTTGCAAACTCGGGGAATCAAACTGAAGCAAGAGCATGTTAAGGTTTTGGATGTCAGAAAATTAGAAGTCGTTCCTGAAGAGGCTGATAGGAGCAGACTTCATTTTCGCCTCTACAATCTAAAGAGTATGCTCCCAAATGTCATGGTGAGGGGTATAAATACAGTTCAGCGTGTTGTCATTAATGAGGTAAAAGAAGAGAAAGAAGACAATAAGTACAAGTTGCTTGCAGAAGGCACAAGGCTTCTAGCAGTAATGGGAACCGAAGGAATTGATGGTTGTAAAACCACAAGTAATGATGTTTTTGAAGTGCAGAGAACACTTGGCATCGAAGCTGCAAGAAATTGCATCATTGGGGAGATAAAGAGTACTATGAAATCTCATGGAATGAGCATCGACGATCGACATATCATTTTTATAGGGGATGTGATGACATGCATGGGTGAAGTTCTTGGGTTCACAAGATTTGGAGCTAAAAAAATGAAGAAGGGTGTTTTGGCATTGGCTTCATTTGAGATGCCAGCTGATCATCTGTTTAATGGTGGTGTAAATGGGAGAGTTGAAAGTGTTAGGGGAGTAAGTGAAAGCATAATCCTGGGTCTGCCAATTCAGATTGGCACTGGAATGTTTAAAGTTAGAACAAAAGTTTCTTCTGGTTTGGGGCAGCTCACAGGTTCTGGACCTCCATATTCAGATGTGCAACTGCCTTATGGGCCTGCAGGCACTACTTTTCCAACTTAA

>Pp08:g2.t1 F-box/kelch repeat

ATGCTACCTAAGCCGGTTCAAGGAGGGCGGGTTTGTGGTTGTTCTAAAGGATGGTTGGTTATGATCAAAGAAAAAGTATTAAACTCCAAACTGTATCTCGTAAACCTAATTTCAAGAGACCAACACCAATTTCTATCTTTGAGAACAATTCCATCTTTCCATAAGTTTGTAAACACAAACAAGTCGAAATGTGGCGGTGCATTGCTTTTCTGTAATTCATTCGCGTTATCTACGGAGGATGTTAAATCAAACAACTGTATTGTTGCAGCAGTTTTTGATGAGTTGACGTTGGGTTTATGCAGACCTGGAGACAAAACATGGAGTGTCTTCTATGTATTAGATGAAAACGAATATCTTGCTAAATTATTGTTTTCTTCTTCCGGCATGCTATACGCCTTAGTTTTTTCGGGTAGTCTTAGTAAGAATTGCATCGTTATTGCGTCTCACAGCTTAAACTTCGGAGATGATACAGTGAAAATTAAATTGGTATATGACAAAACAAAAAGCACAAGGGATTTAAATCCAAGCATTGAGTATCGTGATGACTTCCAGATCTTGTTGAAAGGAATGTACGCCTCTTATTTGTTACAATCAGCAACAAATAATGAAGTCTTCGTGATCCATCAAATACTAGATTGCTTTTTAAGAAGGGATGGTGATGTTGAGGATGAGGACGAGAATGAGGAGGAAGGGACTAATGATGAGGATGAGGAAGGGATTGATGATGAGGAAGAGGCTGATGATAAGGATGATGAGGATGAGGATGAGGATAAGGACGAGGAAGAGATTAATGATGAGGATGAGACTGATGATGAGGACGAGGAAGAGACTGATGATGAGGACGAGGACGAGGAAGAGATTAATGATGAGGATGAGACTGATGATGAGGACGAGGAAGAGACTGATGATGAGGATGAGGACGAGGAAGAGATTAATGATGAGGATGAGACTGATGATGAGGACGAGGAAAAGACTGATGATGAGGATGATGAGGATGAGTACGAGGATGAGGATGAGATTGATGATGAGGATGAGACTGATGATGAGGATGATGAGGAAGAAAAGACTGATGATGAGGAAGAGGAGGAAGATAACAATAATGATGATCAAGATGAAGGAGTTAACAATGGTGAATTTCGCCGCATCGATATTGCTTCTGAAGATGCATTTGGCTCTAAACCATATTTGAGGACAAGTGAATTTAGAGTATACAAGCTTGACCAAGAGAATGACAAGTTTGATAGGGTACAATGTTTGGGGGACGACCAACTATTCTTTTGCTCTGATGCTGAATGCGTGTCCCTGCATCTAGCTAGTAATATCCAAGGATTTGACGGAAATTGCATTTATTTTGCAACAAACAACATATGGAAGTTACATTGTTTAGAAACATATGCATCTCGTGAGATTGGTGTATTTTACCTAGATTCTAGGAGAATTAGGCGACCATTTCCAAGTGTTAAGTTGTCAGTACTGACCAAACCCACTTGGTTAACTCCAAGTTTATAG

>chr5:g1.t1 multidrug resistance-associated protein 9

GATTGGATAGGCACACTGAAAACTCCACAGAAAAGGCTTTTACTAGCCTGAACATAGAGAGTAAAAGCTCTAAGAATGGAAGTTCTTCTGGAGGTAACAAGTCTCAGAAGAACTGGAAGGAAAATGGGAAGAATTGGAATAACAAGTCCAATTCTAATTCCAAACCAAATGCATCAAATGAAGGAACTAAAACACCCTGCAAGCACTGTGAAAAACTGCACTATGGCAAGTGCTGGTTTGAAGGAAAACCAAAATGCAGAGGATGTGGAAAGTTTGGTCACATGGTTAGAAATTGCCATGACAATCAACCTGTGCAGAAGGTGAATTATGCCAATCAGGTTGAAGAAACTAGAACATTGTTTTATGCATGCAATGTCGTGACAGATGTGAAATTGAACAATTCTTGGTATGTGGACAGTGGCTGTAGCAACCACATGACTGGAGATGAAAGGCTACTGATAGATATTCGAAGGGATGTAACTTCCAAGGTGAAAATGGGGACTGGAGAGATAGTTCAAGTTGCAGGAAAAGGTACACTTGTGATAGAAACCAAGACAGGCAGAAAACACATTCAAGAAGTGATGTTGGTGCCTGGTTTAGAAGAGAACTTGCTCAGTGTTGGTCAGATGATGGAACATGGGTATTGTTTAGTATTTGGAAAGGGAATGGTGACAATTTTTGATGATTGGTCACTGCAAAATCCTATAGCTAAGGTTCCAATGACTAGCAATAGGTGTTTCTCTCTCACAATGGTACTTGCTACTCAGTTGGTGCTGAGAGCAAGTGTGACTCACAGTTTGCAGACTTGGCATAAAAGGCTGGGCCATCTAAATGATCAAAGCATCAGGATGCTGGCAAATCAAGACATGGTTCATGGACTGCCTAGTCTGGAGAAGGATTTTGCAGTTTGTGAAGGCTGCAAGCTGGGAAAGCAACATAGAGACTCATTTCCTGCAGAATCTACTTGGAGAGCTCAGTTTCCACTTGAATTAGTTCACACTGACATCTGTGGTCCAATGCAGATTGCATCAATGTCAGAAAACAGATATTTCCTGTTATTCATTGATGACCATACAAGAATGGCTTGGGTTTATTTTCTCAGAAATAAATCTAATGCTTTTGAATGTTTCAAGAAGTTCAAAACAATGACAGAATTGCAGAGTGGACACAAAGTGAAATCACTTAGAAGTGACAGAGGTGGAGAATTCATGTCAAATGAATTTCTTGCATGCTGCAGTGAGGCTGGAATACAAAGACAGCTGACAGTAGCATATTCCCCTCAGCAAAATGGAGTTGTTGAGAGGAAGAACAGGACTGTGATTGAGATGGCAAAGTCTATGCTACATGAAAAGAGTCTTCCATATGAGTTTTGGGCAGAAGCTGTTCATACAGCAGTCTACCTTCTCAACAGGGATGTCTATTTTGATGAAGAAGTCTCATGGAAATGGGAGAATCCTAGCAATGCAGATGTGAGAATGCCTATGCCAGATGAAAATCAAGGTACTGCAGAAACTGAACAGAGGGTATTAGATGAACAATCTCAGTTTGTGGATACTCAAATGCAGATGGAAGAAGAAACTGCACCTCAAGGGGAAGAAATGCTTGATGAGACTCAAAGGCTTGATCACACACCTCACAAGTGGAGGAGTATTAATCACATCATGGCACAATGCAACATGTGCATTGTAGAACCTGACAGCTTTGAAGAAGCAGATTTGGATGAATCATGGAGAAGTGCAATGGAGGCTGAATTGGAGATGATTGAGAAGAACAATACTTGGAAATTAGTTGACAGGCCATCTAACAAACCAGTAATTGGTGTTAAGTGGGTCTACAAGGTCAAACTGAACCTTGATGGTACTGTGCAGAAGAATAAAGCTAGGCTTGTGGCTAAAGGCTATTCACAGAAGCCTGGAATCGACTATAATGAGACATTTGCCCCTGTGGCAAGACTTGACACCATCAGAACCTTGATAGCTCTTGCAGCACAAAAGGAATGGAACCTATTTCAATTGGATGTGAAGTCTGCCTTTCTCAATGGCATTCTAAAGGAGGAAGTTTATGTTGAACAACCTCAAGGATATGTTCAAGAGAGCAAGGAAACCAAAGTGTACAGGTTGAACAAGGCTCTATATGGACTGAAACAAGCCCCAAGGGCCTGGTATGATGAAATAGATGCCTATTTCAACACTGCAGGTTTTAAGAAGAGCTTAAGTGAAGCTACTCTCTATATCAAAACAAGTGACACCTCAGGTATTATCATTGTCTCACTCTATGTAGATGACATTATATATACTGGAAGCTATCCTAAAATGCTTGAAGAATTTAAACAAGATATGATGCAACACTATGAGATGACAGACTTGGGTCTGTTGCACCATTTTCTTGGCATGGGAGTGGAACAAACTGATAAGCATATTTTCATTCATCAAAAGAAATATGCCATGAAAATTCTTGAGAAGTTTGGAATGAGAGACTGCAAGTCAGTGGCAATTCCATTAGTGGTGAATGAGAAATTGTGTAGAGAAGATGGAAGTGAAGCAGCAGATCAAAGTGAATTCAGACAGATTGTAGGAAGTCTGCTTTATCTGACTGCCACAAGACCAGATGTCATGTTTGCATCTAGCTTGCTTGCTAGGTTTATGCACAATCCCTCAAAGAAACACATGGGAACTGCTAAAAGGGTGCTGAGATACATACAGGGCACATTGGATTTTGGAATTGAGTTTGAAAAGGGGAAAACAGCTACTCTAATTGGGTACTGTGACAGTGACTGGGCTGGGAGTGAGGATGATATGAGGAGCACCTCAGCATATGCTTTCGCACTAGGCTCAGGCATGTTCTCTTGGGCTTCCATCAAGCAAAACACAGTTGCTTTATCTACAGCCGAAGCTGAGTATGTGAGTGCTGCAGAAGCAACTTCACAAGCTAAGTGGCTCAGATTTGTGCTTGAAGATTTTGGTGAAGAACAGATAGAAGGGACTCCAATTCTATGTGACAACACCTCTGCAATTGCAATGGCAAGGAATCCAGTTCATCACCAAAAGACAAGACACATCAGCAGGAAATTTCATTTTATAAGGGAAGCCATTCAAGCAAAAGAAATTGAACTGGTGTACTGCAAGACAGAAGATCAGATAACAGACATTTTGACTAAGGCTCTGCCTAAGGATCGGTTTGTGAGGCTGAGAAGCCTGCTTGGAGTGAAATCAGCTAAAGGATTAGAAGGGAGTGTTGAAATATAA

>Pp05:g1.t1 non-LTR retroelement reverse mRNAase

ATGAAGGTGATGGTGGCTAGAAAAATCAAATCTCTGACAGGGCAAGTTCTAAAGGTTGATCAATCTGGGGGGAGGGATTGTATTGGAAGATTCTTATGGGTCAAGATAAGGATGGATGTATCGCTACCGCTCATGCGAGGGATATTCATGGGATTTCTGGAGGAAGGGGCCAAATGGGTGGATTTTCGATACGAATATCTTTTGGAGTATTGTTCTCATTGTGGGTGTTTGGGGCATCCAATGTCGAGCATACGCAGCTGGGGACGACACCAGGAGAAATATGGAATCATTAAGGGAGGAAAAAAAAGTGTTTGTAGGTCTGGAGGCTTTGACGAACCTCTAAGGCTGGGCGTTGAAAGCGGGAGGAAGACGACCACAATACAGTTTGCCCAACTCTTCAGGCCTTGTGAAGTGGATACAAAAGCAGTGAGAAAAGGAGGAACGCTAGTGGTTGATGCAAGAATAAGCTTGGGAACTTGGTCGGAAGGGGTGGCACGTAGATCCTTAAAAATTTCTGAGGAGGATTGGAATGCTATCAACATGAATATGGTGATAAAGTTGGATGGGGATACGAATATTCAGATTGACAACGGTCAACTTGAGTTGCTTACTATAACCATGGGAGAAGAGGGACTGAGAAACTTGACTCAAGACTCTGACCCATTTAACTTGGGGGGATTGGAAGACCCAAGCGGATCATGGCATGAAGGTCTTGCTTATATTAAACACATTGCAGTATCTTACTTTAAAGAGCTCTTTACTACTAGCCACCCTACAAGAATTGCAGAGATAATGAGGTGTGTTCAAGAGAGGATATCTGCACAAGACAATCTTCTTTGGACACAGCCTTTGGATGCGAATGAGGTGGTTTGGGCAATGAAGCAGTTTCACCTCACGAAGTCCCCAAGACTAAACGGATTTATAGAGGTGGAAGGGGAAGGAAATATTGAGATGGCTCTCAAGTTAGATATGGTGAAGGCATACGATCGAGTGGAATGGGCAAATGAGCAGGAGGCAGTACGAATCCGTCAGTTGCTAAAGCAATATGAATTGGGATCTGGACAAACTATTAATCCATCAAAGAGTTCTTTATTTTTTAGTAATAACTGTTTGATAGCTGAAACTAAGCAGTTGAAGTTGTTGACAGCTCAGTCCAGCACAGACACACTCAGTCCAGCCTGGATCAGAAGTTTCTACCCAGGCAGAAGGAAGGAAATTCTGCTCAAAGTCGTGGCCATGGCCATGGCATTGCCAAATCATGCCTTTTCATGCTTGAAGCTACCGTTTGGTTTGTGCAACGAGCTTAAAAGACAGATTGCAAATTATTGGTGGAAAGAAAACGAAGACATTAAAAGGATTCATTGGGTTGCTTGGAAAACAATGAAGAGGTTAAAGAAACATGGAAGCATGGGTTTCCGTGATCTGGAAAAATATTTTGAGGGAAAGGTGTTTATGGTGGCAGAAGCTGGGCCAAGAAAGCTTAGGAGTGCTACTTTTAATGACTACTGGACGAAATTAATCACAATGCGGCTATCTTTGAAGGAAATGAGTGAAGTTGGACGGGTGGTTCATTCTTCCTTAGGCTTCCTACTCATGACAGGAGGATCTAGTAACATCCTATGTGAATTTGCTCTCGTGATGGAAGTTGAAACAGTTCTCGAAGCAATGATGGCGGTGATGGATGTAGGGGTCGACAAAGTTATGGTAGAAACTGATTGTCAACAGCTTATTAAAATGTTACAAGACAAGGACACACTTGATATTACCTTAGAAGGGTTAATTCATGATATTAGGGTAAAGTTTCTCATTGCCTTGAGAACCATTAACTGGTCAACAAGTCAACTTTCTTTAGCAGAACTGCCTTCTACAAAACTAGAAAGGGAAAAATCAACTCTAGAAAGACAAAAGATAGCTGGAATTCCATTTCTGCCTGGACAGAAGCTTCCGATCTGGATTGGACTGTGTATGTCTGTGCTGGACTGGATTGTCAATAACTTCAACTGCTTGGCTTTTGCTGTGAATCGATACAAAAGTTCAAGTTTGGCAGAGCTTAATCTATTTCAAGCCCTGGAAGGCTTTTTGAAAGGACAGAATTGCGTCCTCTTCAGTCTGAAAGGGGCAGAAGTGGCTGGATTTGAGGATTTAATGAGCTGA

>chr6:g1.t1 retrotransposon

ATGTTTTTCATTCTCCTACTTCATTTACCATCGCACCACACTCTGTTTTTTCCCACCCAGACTTCCCTTCTGCCCACCCCAAACTTTTCCCATGGATCTCTGAGGCCACTTAAACTTGATCTCGAACGCTTTTCCGAGGATGACCCTTACGGATGGATCGCTTCTGCGGAGCACTTCCTCAAGTATTATGGGGTGCCTGACGAAGATAAAGTTATTGTCCCTGCTGTTCACCTCTCCGGCGACGCTTCTTTATGGATGTGTTGGTTCGAACAGCGCTTTCCAAAAGATTGGACTTTTTTTACCACATCCTTGCTTCAGTACTTTGGCTCGACAGATATGTGCGATTTTGAAGCCTCCCTCTCTCATGTCCAGCAAACTGGATCTTTAGCTGACTATCTTACCCTCTTTACCAAGCTCGCTTGCAGAGCCTCGGAATGGCCCGACGTACCTCTGCCCGAACCTTTTACCCCCGTACCTCTCCCAACCCCCAACCACACTCCCACAACTCTCCTTCACCTATGCCACCTTTATCTTCACCCTAGCCCTTACCCCCTTCTTCCCGCCACCTCTCCCAAGCCGAAGCACAGAAGCGTCGCTCCAAGGGCCTCTGTTTTACCTGCGACGAGAAATACAGACCTGGTCATCGTTGTCTCAAGCCCATGCTTGCTCTCATTGAGGCCACTGTTCCGGATGATGACTCATCAGTATTTCATGATTGTCACCAGGAGTTGGCCTCTCCAGAGCAGATTTTGGAGCAACATTCAATTTCCTCAACCCAAGCCTTGCTACATGTTTAGGCTTCCCAGTTTATCACTCTGCCCCCCAAACCCTATACACAGCCACATGGGAACAATTACTCATGCAAGGCACCATTCAAAACCTCTCTGTCCAGATTCAAGATTACTCTCTTCTAATTTCAAGTTATTACCCGTTTCTGGTTGTGATATATTATTGGGCGCCGAATGGCTCGAGACATTGGGTCTTATTGAATGGGACTTCAAGAATAAGATTATGCATTTCCACCTTGGCGAACACTCCTATTGCTTGACAGGTATCCATAGCTCTCCCACCACTGCAATTGATGCCAAGCTCATGACTCAAACTCTCTTGGCTGAACCAGAGGGCTTCTTGGCACAGCTTATCCTGTGCTTACCAGACCATGGAGACAATACAACCACTGCACCACCTCTTGCTCTCCACCACCTTTTGCATACCTTCTCTGACTTGTTCAATACCCCTGCCGCATTACCGCCACCTTGCCATATTGACCACCGTATTCCCTTACTTCCGGGTGCTACCCCCGTCAACGTCCGGCCTTATCGATACCCGCATCTCCAAAAATCAGAGATTGAAAGCCTCATCCATGAAATGCTTGCAGTGGGCATCATTCGACCAAGTGCAAGCCCTTACTCCTCACCAGTTCTTTTGGTTAAAAAAAAAGGACGGCTTTTGGCGTCTATGCGTCGATTACCAACTGATCACCTCTTTGTGAAGAAATCCAAGTGTGTTTTTGCCCAACCACAGATCGAGTACCTTGGCCACACCATTTCCTGCCAAGGCATTGCAATGGATCAAACAAAAATTGATTGCATTCAAACATGGTCCAAGCCGTCTTCCCCAAAGTCTCTGAGCGGCTTTCTCGGACTTGCAGGCTACTACCGTAAGTTCGTGAGGAACTTTGGTTTCATTGCTCGCCCACTAACGCAGCTACTCAAAAAAGACAATTTTGTGTGGAACCATGAAGCTGACGCTGCTTTTGCTGCCCTAAAAAATGCATTATCTTCCACGCCAGTGCTTCAGCTGCCCGATTTTTCCAAACAGTTTACAATAGAATGTGATGCCTCTCAAGGAGGACTAGGTGCTGTTCTTTCACAAAATGACCACCCAATTGCCTTACTTTCCAAACCTCTGTCAGGAAGAAATTTGGCACTCTCCGTCTACGAAAAAGAAACGATGTCCGTCATTTTTGCAATTAAAAAATGGCTCCCATACCTTCTTGGCCAACAATTCCGCATCATCACAGACCACCAGACCCTTCGCCACTTCTTGGATCAACGTATTACGACCCTGATCCAACAACGCTGGCTGCTCAAACTAATGGGTTACAACTTTGTGCTCCATTACCGACCTGGCTCCCAAAACTCCGCTGCGGATGCTCTGTCACAACGCCATGAACTCCTTCCTCTCTTGGGCATCTCTCAACCCATTTTTACATATCTGGATACCATCAAGGAGGATTGTCATAAAGACCTCGATATACACAGTTGCTTATCCAGTGCCTCTGCACCAACACCCCCCCTCTACCTAAGGGATTTTCGTGGCAAAATAACACCCTCTTCTACAAGGACAGGTTGTTTGTTCCTCCGATCAATGATTGGCGCTCCAAAATCCTCCAGGAATTCCATGCCTCCCCCACGGCTGGCCATTCTGGTTACCCCCGCACTGTTAAACGCGTTCAAAGTAACTTCATGTGGCCCGATTTACGCTCTGATGTCAAAGCATTTATCACCACCTTACCTGCCAACGCCAACACTACGAAGCCATCCACCCTCCCGGTCTATTGCAGCCTCTTCCAATTCCAGCCGCTTCTTGGCAATCTATCAGGATCCAAACTCTGCCAATCATCTGCGTACCATACTCAGTCAGATGGCCAAACTGAGGTCATCAATCACATTTTGGAACAATACCTCCGCTGTACCATTGGCGACAAACCCATCTCTTGGACCACATGGCTCCCATGGGCCAAATTTTGTTATCGCATACTCCCCTGGTTCTACAGCAGTCCATGTCGTTGACACAGCCCTCATTGATCGTGACCAGCTATTGAACACCCTCAAATCCAATATCCAAATGGCCCAAAATCGAATGAAGGTTTATGCCGACAAACATCGTACCGAGCGCCACTTTAACGTCGGTGATTATGTCTACCTTCGGCTACAGTCATACCGCCAACACTCTGTCATTTTCCGCCAGAATCACAAGCTTTCTCCTCGCTTCTATGACCCCTACAAAATTGCAGCCCGTGTAGGCCAAGTGGCGTATCGCCTAGAGCTGCCAACCAACAACAAAATACATCCTGTTTTTCACGTTTCATTGCTCAAACTCAAGCTTGGCACTACCAGCACTGCAAGCACTCAGCTTTCCCCCATGTCTTCTTCGGGCGCACAAACTTGGAAACCTGAAGCAATCTTATAA

>chr8:g1.t2 Retrovirus-related Pol polyprotein from transposon TNT 1-94

GCGCCTTTGCTGAGCATGAGAAAAGGTTTAGGGAAGACATAGAAAAGGTGAAATGCTGGAGAGTTGCTTTAACAGAAGTTGCCAATTTATCTGGGTTGGATTCAAAGAATGAGTGTGAAAGAAAGCTCATTGAAAAGATTGTTGAATGGGTGTGGGGGAAAGTTGTTGGTTGTAAGACAGCTAGAGAAGCATGGCTTAATCTCACAGACCATTATGCATCTGTATCAAGGTCTCGTGTCACACAGTTGAAAACTGAGCTTCACACAATTAGTAAAGGTGCAGATTCTATTGAACGATTCTTACTCAGACTTAAACATCTTAGAGATCAACTGACAGCTGCTGGTGTCAAGCTGACCGATGATGATATTATTATTGCGGCTCTTAATGGTCTTCCTGCTGAGTTTGATATAATCAGGACTGTTATCTTTGCTCGTGAGACTCCCATCACTATGAAGGAATTCCAAGCACAATTGCTTTCTGCTGAGCGTACCATAGAGTCACGGGTGATGGCATTGTCTCATCAAATGTCTGGTCTCATGAGTTCTGTTTCTTCCACTCAAGGCTCTAGCTCACAGTCTTCTTCTCTCTCAGCATCGACTGTTGGTATTATTCATGCACAGCCTACATCTTCTCATGGTTTTACTGGTGTGAAACAGTCAGGGCATTCTGGTTTTCGATTGACTTCTTATTCCCGATATCCACAACCCTCTCAAGGATTTTCTTCAGGGTCTGTCAGAGGCAGAGGCAATTACAATTCATCACCACAGTATTCAAATCCAAGAGGGAATTTTCAGTCTCGTCCTTCTTTTGGTTCCTCTGTTCAAAGGTCTCAAGTAATTCCTGAGTGCCAAATTTGCAACAAACGAGGACATACTGCTCCAAACTGCTTCTATCGAGTTCCTGACAATTCTTATGTGCCTAATCCTGTTGTGGAATGCCAGATTTGTGGAAAGAAAGGTCACACAGCCTTGAACTGCTACCATAGAGCTAACTTTTCCTATCAAGGCACCGCATCTCCTGCTCAGCTCCATGCAATGACAGCTCAAACCTCTCCTTCATTTTCTGCTGATGAGTTTTGGATCGCCGACAGTGGTGTATCTCATCATATGACCAATAATGTGACTCAGTTGGCTCAAGTTGCTCCTTATACCGCTGATGAGAAAATAACTATTGGGAATGGCGAAGGTCTGTGCATTGCTTATGTTAGCAATGCCTCCATTCCTAGTATTTCTGGTTCTCTTCGCCTTAATCAAGTTTATCACGTGCCTCAGTTGGCTGCCAGCTTGTTATCCATTTACCCGTTGTGTAAGGATAATAAATGCTGGGTGATTTTTGATGACTCATATATCTATGTGCAGGACAAAGCAACGAAGGTTCTGCTGTACAAGGGTCGCAGTAATAAGGGTCTGTATCCAATTCCTCAAGCTCTGGGTGATTTGTCAGTAAGGTCCAATCCCAAGTCTTCACCAATTATAGTCGAGGGGATTGCCTCTACTCCAACAGCTTTACTTGGGAAACCAGTATCTTCAGTTCTTTGGCATCAAAGATTTGGTCATGTGTCCAACGAAGTTCTTACTCAGATGTTGAAACAGTCTCAAATTTCTAGTGTTTCAGATTCCTCTCAGAGTCTATGTAGTTTTTGTTTGAGTGGTAAAATGCACCGCCTTCCTTTTGCTAGTTCACAGTCTCATTCTTCATTACCTTTTCAGCGATTACACTCTGATGTATGGGGTCCTTCCTCCTCTGTAGCATTTGGTGGATATAGATATTATGTGTCAATCATCGATGACTGTACTCGATTCCTCTGGATTTTTCCATTAATCAATAAATCTGAGGTTTTTCCCACGTTTGTGAAATTTCATGCGTATATTACTAAGCACTTTCAAGCTTCTGTGCAATATTTTCAATCTGATGATGGTGGGGAGTATAATAGTAAAGCATTTAAAGATTTCTTGGCTTCTAAGGGAATTCTCCATCAGATTTCATGTCCTTATACCCCTCAGCAAAATGGGGTTGTTGAGAGGAAGAATAGGCATATTATTGAAACCACAATTACATTACTTGCTACTGCTGCCTTGAATGACAAGTTTTGGTACTATGCTGCAGCTCATGCTGCCTTTCTCATAAATCGTATGCCTTGTCAGCTTCTTCAAATGACATCTCCATACTTCAAATTGTTTGGTCATAATCCTGAGCTTCAATCATTAAAGGTTTTTGGATCCGCAGTATATCCACTTCTTCGACCCTATAATTCTCACAAGTTAGAGCCACGATCAGCAGAGCATGTGGTCCTTGGATATTCTCTTGGTTATAAGGCTATAGCAGTTTCATTGCCTTATAGATATGTTACTACCATAGAGTCTGATCACTCTCCTCTCCCATGTTCAAATGAGAATTCATCTTCTTCAGTATCTACATCTGAGGCTCAAGAGGTCTTAGCATGGTCATTCTATATGCTAACTCGTCTTAAAAGTGGTATTAGCAAGAAGAAAGATTTTGGTGATTTTCAATGTTTTTCCACTTGTTTATCTACAATTACTGCTCTTGATGAGCCTCATACTTTTCGAGAAGCTAGTACAAAGTCTGAGTGGCAGCAGGCTATGATAGAGGAAATTCAAGCATTACAAACTCAAGGTACATGGGATTTAGTTCCTCCACCCTTTGATAACAATATTGTTGGTTGTCGTTGGATTTACAAAATTAAAAGACATGCTGATGGACGTATTGCTCGATATAAAGCTCGTTTAGTTGCACAAGGTTTTAGTCAAGAACAAGGTATAGATTTTGACGAAACCTTTAGTCCTGTAGTTCGTCATACCACAGTTCGATTGATTTTAAGCTTGGCTGCATCTCATAGATGGTGTTTGCGCCAATTGGACGTTAAAAAAGCCTTTCTTCATGGTGACCTACAGGAGGAAGTGTACATGAAACAGCCTTTGGGATTCATTGATGATCATTATCCAGATTATGTATGTCGGCTACGAAAGTCTCTTTACGGTCTTAAACAGGCACCACGTGCTTGGAATGCCAAGTTCACTGGATATCTTCCAGCTTTGGGCTTTGTTTCTTCACACTCCGATCCTAGTCTCTTTGTGAAGCATGATGGTCCTAATGTGGTTATTCTTTTGCTGTATGTGGATGACATTATCATCACTGGGTCAAGTTCTACCCTAGTGCAATCTGTGATTGATGATTTGGGACAAGTGTTTGATATGAAAGATATTGGCCAGCTTACTTATTTCTTGGGCTTGGAGGTTTCTTATCAGTCCAATGGTGATTTGTTTGTCAACCAAGCTAAATATGCTCGTGATTTACTTAAGAGAGCTGGCATGGAAACTTGTAAACCATCAATTACACCTTGTAAACCTCATTGTCATGTTCTGACAACTGATGGTACATTACTTCCTGATCCTACAATGTTTCGCAGCTTGGTTGGAGCTCTTCAATACCTCACATTCACACGCCCAGATCTGGCATATGCTGTGAACACCGTATGCCAGTTTATGACTGCTCCTACTGATATTCATGACTTTAATTGGGCTGGGGATCCCAATACTCGTCGCTCTACTACAAGGTACGTTGTATTTCTTGGACAGAATCCTATCTCTTGGTCCTCTCGTAAGCAATCTTCTGTCTCTCGTAGTTCTACAGAGGCAGAGTATCGAGCTCTTGCCAACTGTGCTGCTGATATCTCTTGGATTAGATATGTTCTTCAAGACTTACATATTGTTGTTCCTGAAGCTCCAGTCTTACATAGTGACAGTTTATCAGCATTGGCCTTAATCCAGTTTTGCACTCTCGCATAA

>Pp02:g1.t1 reverse mRNAase

ATGGAGAACATCAAGAGCCAGTGTTTTGAGCTTACCCAGCGGAGATTTGAGGCATGCACTGCTGCCAGGTGGAGGTCTCAAGAATTGGGGGAAGTTCCTTCTTCTTCACTTATAATCCCTTACATGCATTGGTGGTTTTTTCTTTGGGTTAATCATTATTACCTACGTACATTCCCTATTTATACTGTTGTTTGGAAGAGTTTGCTTATCTGCTTTCAAGTCTCTCATTGCTTCTACAATCTCTGCTTTAAGTTGTGTTCTTGTGTCACTATGAATCCCCAACTGACTGTCAGCTCTTCTCACACTAGGTCTCGTAGTACCAGTCCTCGATCCCCTTTTAACCTTAGGAGAACTCCTCCCCGTAAGCCTTCCATAATTCACAAAGGTCTTTTTACCTTCACCATGGAAAATAACGAAACTAGGGTATCCACCACTGCTCTAGCTTTTAGTGTGCATAGTCATTTTGAAGCTCTCTGTTTTGTTGGGAAAGTCTTTGGCGTCCCTGTTCCTGGGAGGGCCATTAGGAATAGGTTGAAAAGCTACTTGAAAGACCTGCAAAAAGAAGTCTCTGTTGACCACATTGGTAGAGACTGGTGGAGACCTGACTTCTCTCCATTTCATGCTTCTATTGATTCTATTGTTTGTTGGGCGAGAATTCCTTTCTCGCCGTTACATTATAAAGATCCTGAAGTTTTAAGTGATTTGGTCTCCATCCTTGGGACCCCTATTTGCATTGACCAAGCTTCCATGATGGGAAAACAAATGGAGAAGCTGAGCAATGAACCTAGTTTCTTTCCAAGGGACTTGGTGTTGGATGAGGAAGATAGGAGGGATCTGCAAGACGATGTGATCTTTGTTTTTCCTCAACCCATTATAGTAGAAGACATTCATAAGAATGAGAATGATGAGGAAGTAGAGGAAGGTAACATGCAGAATACTGAGGAAAGAAAGGTGGGTTGGACTGTGGTGTCTAGTCAAAAAGGAAAAGGCAAAGGAGCCATAAGGGATGACAGATCCTTTAAGGAAGTGGCGAAAGGTATTAGAATTGCTGATGAGAACTCTGTTCCATTCCCCAAAGTCCACTTTAAAGGCGAGGGGCCTAGTGGGGTAGATAAAGGAAAGTTGGTGATTTCGGAGCCGAGCCTTTCTCTGGAGGAGCATAGCTCTTCCAATTGTAAGGCTTTTAAGAGCCCTAAAAAGAGAGCTAGAGAGCTCTTTGAAGAAGTTTCGGAGGAGTCAAAGCCAAGTGTCAAAATTCTCGGTGTTTTTCGCCCAGCTGACCGTGAAGATTACACTCCCAATCCATGA

>chr2:g1.t2 reverse mRNAase

GATTATATGGAGAACATCAAGAGCCAGTGTTTTGAGCATAGCCAACGGAGATTTGAGGCAAGCACTGCTGCCAGGTGGAGGTCTCAAGAATTGGGCTCTTCTCACCCTAGGTCTCGTAGTACCAGTCCTCAATCCCCTTTTAACCCTAGGAGAACTCCTCCCCGTGAGCCTTCCATAATTCACAAAGGTCTTTTTACCTTCACCATGGAAAATAACGAAACTAGGGTATCCACCACTGCTCTAGCTTCTAGTGTGCAGAGTCCTTTTGAAGCTCTCTGTCTTGTTGGGAAAGTCTTTGGTGTCCCTGTTCCTGGGACGGCCATTAGGAATAGAGACTGGTATAAGACTGAGTTTGGTGCTGAGGAGGATGTCGAGTTTGGGCTTAAGCATAGACCCTGGTTTGTCCAAGGTCAAATTTTTGCTTTACAAAGGTGGAGACCTGACTTCTCTCCATTTCATGCTTCTATTGATTCCATTGTTTGTTGGGCGAGAATTCCTTTCTCGCCGTTATATTATAAAGATCTTGAAGTTTTAAGTGATTTGGTCTCGATCCTTGGGACCCCTATTTGCATTGACCAAGCTTCCATGGTGGGAAAACAAATAGAAGACATTCATAAGAATGAGAATGATGAGGAAGTAGAGGAAGGTAACAGGCAGAATACTGAGGAAAGAGAGGTGGCCTGGACTGTGGTGTCTAGTCAAAAAGGAAAAGGCAAAGGAGCCATAAGGGATGACAGATCCTTCAAGGAAGTGGCGAAAGGTATTAGAATTGCTGATGAGAACTCTGTTCCATTCCCCGAAGTCCACTTTAAAGGCGAGGGGCCTAGTGGGGTAGATAAAGGAAATTTGGTGATTTCAGAGCTGAGCCTTTCTCTGGAGGAGCATAGCTCTTCCAATTGTAAGGCTTTTAAGAGCCCTAAGAAGAGAGCTAGAGAGCTTTTTGAAGAAGTTTCAGAGGAGTCAGAGCCAAGTGTCGAAATTCTGAGGGTTTTTCGCCCAGTTGACCTTGAAGATTACACTTCCAATCCATGA

>chr6:g2.t1 rust resistance kinase Lr10

ATGGATAGCCTGCTGCTCCTGCTTCTGTTTTTCTTGCTTGTAACTACTGTACCAAGTAAATCTGCAGCTGATGATCATCACGTTGACTGCCCAGTTTCCCGGTGTAGTAGGGATGATGGTCCAGTTGTCCAGTTCCCGTTCCGTCTCAAAGACGACCCCCTTCACTGTGGCCATCCACAGTTTGAGGTTTTTTGCTCCAAAAATATGACAATGATTCAGCTGTCGTCATCATCAGGGCTGTTTCCCATACTAATAATCGATTATGAATGGCGAAAGTTTGATGTCTACGATACGAATGATTGCCTCCCGAGACGCCTTCTGAACTTTACCATCTCCAGCAACTCACTCTTTCATGTCCACAATTCCGGCTGGAATTCCTATTCTCATTATACAGAGTGTCCTGATGTGTCAGCAAACTTGACATTATTAAATTGTTCCACGGCACAAAACTTCAATGCAGCCTCTATTATGCCAATCAGCTGCCTCAGTGTCCCGGGGCATCAAGTTGTGGCTGTTCCACCTTCAACTTCTATCACTGAATTTCCTGGGCCACTGCCAACTTGTGGCACCTTGGGACAACTCTATCTTCCGGTACGAAGAGTGTATCTTCCAAATTTTAACGAGCACCTCTGTGAACTGGACGATGTTTTGTTGCTGGAATGGGAATCATGGGATTATTTCCCACAATGTCAAAACTGCACAGCAGGTAGAGGAAGGTGCGAATTCAATAACACCATCAATCAACTTGAGTGTTATCCTTACCCAAGAGTAAGGATTCCACAAATGCCGCCACCACAACACCCAAAAGGTCATATATCGACTAGGCATATAATCATAGGAGTGAGTTCGGCTTGCTGTTTTGTTCTCATATGGGTAGTGGCGGTAGCAATTTTCTTCCATGTAAAACAACAAAGAAATAGCATAGAGGAAAAGGAGAATCAAATAAAGGTTGAAAAGTTTCTAAATGATCACAAATCTCATGTACCAACAAGATACTCTTATGCTGATATAAAGAAGATTACAAATGGATTTAAGAAGAAGTTAGGGGAAGGCGGTTTTGGAAGCGTTTTCAGGGGAAAGCTTCCCAATGGAGTTCCAGTTGCCGTAAAAGTCCTCAGTGATTCTAAGGGAAATGGGGATGATTTTGTTAACGAAGTGGGAACTATTGGCAGAATTCACCATGTTAACGTGGTTCGTCTACTTGGGTTTTCTGCTGAAGCAGGCAAGCGTGCAGTTATTTATGAGCTCATGCCAAACAGGTCCCTGGAGAAGTTCATCTCGTCTAAAGATCAATCTAATAATGCTTTGTTCGATTGGGAGAAACTTGACAACATTGTCAAAGGTATAGCAAAGGGAATTGAGTATCTTCACCAAGGGTGTGAACAGAGGATCCTCCACTTCGACATCAAGCCTCATAACATCTTGCTAGACCATGACTTCAATCCAAAGATCTCTGATTTTGGTGTGGCTAAACTGTGTTCCAAGGAAGACAGCATTATATCTATCACTGCCGCTAGAGGCACAGTAGGCTACATTGCACCAGAAGTGTTCAATGGGAACTTTGGAAGCGTGTCCCACAAGTCAGATGTGTATAGTTTTGGGATGCTAGTGCTTGAAATTGTTGGAGCTAGGAAGGAAGCTGCTCTTACATCTGGCATCACAAATGAGGCCTACTTTCCAGAATTGATTTATAAATGTCTCATTCAAGGAGAAGCACTGGGTTTGGAGCTAATAAACATGGATGAGGATGCTGAGATTGCTAAGAAGCTGGTGATTGTTGCACTTTGGTGCATCCAGTGGTACCCGGTGAATCGGCCTTCCATGAAAGCAGTTGTTAGAATGCTAGAAGGAGCCTCTGAAAACCTGATCATGCCGCCGAATCCGTTCGCATCCGCAACAAGTACTCAGAGTCAGACAGAGCAACCAAAAACAACAGAGTCAAGTTAA

>Pp03:g1.t1 transposable element 1

ATGACGTTTGTGATGCCTAAAATCGAAGGACTTCTAGGAATTCTTACCATTCGTTTGTCTGAGGATAACTTTGTTAAGTGGCACTATCAGTTCCAATCAGTGCTACAAGGTTACGATTTCTATGGTCACTTTGATGGCTCTAGTAGTTGTCCACCTAAGTATGTTGTCACAGAGAGTGAGGGAGTTACTGGTGAACTTACTGAAGCACACAAGCAGTGGATACAAATTGACAAAGCTCTATTGAGTTTATTGATTGCAACTCTTTCAGATGATGCGATTGAATATGTTGTTGGTTGTAAGACAGCTAGAGAAGCATGGCTTAATCTCACAGACCGTTATGCATCTGTATCAAGGTCTCGAGTCACACAGTTGAAAACTGAGCTTCACATAATTAGTAAAGGTGCAGATTCTATTGAACGATTCTTACTCAGACTTAAACATCTTAGAGATCAACTGACAGCTGCTGGTGTCAAGCTGACCCATGACGATATTATTATTGCGGCTCTTAATGGTCTTCCTGCTGAGTTTGATATAATCAGGACTGTTATCTTTGCTCGTGAGACTCCCATCACTATGAAGGAATTCCGAGCACAATTGCTTTCTGCTGAGCGTACCATAGAGTCACGGGTGATGGCATTGTCTCATCAAATGTCTGGTCTCATGAGTTCTGTTTCTTCCACTCAAGGCTCTAGCTCACAGTCTTCTTCTCTCTCAGCATCGACTGTTGGTATTATTCATGCACAGCCTACATCTTCTCATGGTTTTACTGGTGTGAAACAGTCAGGGAATTCTGGTTTTCAATCGACCTCTTATTCCCGATATCCACAACCCTCTCAAGGATTTTCTTCAGGGTCTGTCAGAGGCAGAGGCAATTACAATTCATCACCACAGTATTCAAATCCAAGAGGGAATTTTCAGTCTCGTCCTTCTTTTGGTTCCTCTGTTCAAAGGTCTCAAGTAATTCCTGAGTGCCAAATTTGCAACAAACGAGGCCATACTGCTCCAAACTGCTTCTATCGAGTTCCTGACAATTCTTCTGTGCCTAATCCTGTTGTGGAATGCCAGATTTGTGGAAAGAAAGGTCACATAGCCTTGAACTGCTACCATAGAGCTAACTTTTCCTATCAAGGCACTGCACCTCCTGCTCAGCTCCATGCAATGACAGCTCAAACCTCTCCTTCATTTTCTGCTGATAAGTTTTGGATCGCCGACAGTGGTGCATCTCATCATATGACCAATAATGTGACTCGGTTGGCTCAAGTTGCTCCTTATACCGCTGATGAGAAAATAACTGTTGGGAATGGCGAAGGTCTGTGCATTGCTCATGTTGGCAGTGCTTCCATTCCTAGTATTTCTGGTTCTCTTCGCCTTAATCAAGTTTATCATGTGCCTCAGTTGGCTGCCAGCTTGTTATCCATTTACCAGTTGTGTAAAGATAATAAATGCTGGGTGATTTTTGATGACTCATATATCTATGTGCAGGACAAAGCAACGAAGGTTCTGCTATACAAGGGTCGCAGTAACAAGGGTCTGTATCCAATTCCTCAAGCTCTGGGTGATTTGTCAATAAGGTCTAACACCAAGTCTTCACCAATTGTAGTTGAAGGGATTGCCTCTACTCCAACAGCTTTACTTGGGAAACCAGTATCTTCAGTTCTTTGGCATCAAAGATTTGGTCATGTGTCCAACGAAGTTCTTACTCAGATGTTGAAACAGTCTCAAATTTCTAGTGTTTCAGATTCCTCTCAGAGTGTATGTAGTTTTTGTTTGAGTGGTAAAATGCACCGACTTCCTTTTGCTAGTTCCCAGTCTCATTCTTCATTACCTTTTCAGCGATTACACTCTGATGTATGGGGTCCTTCCTCCTCTGTAGCATTTGGTGGATATAGATATTATGTGTCAATCATCGATGACTGTACTCTATTCCTCTGGATTTTTCCATTAATCAAGAAATCTGAGGTTTTTCCTACATTTGTGAAATTTCATGCGTATATTACTAAGCACTTTCAAGCTTCTGCGCAATATTTTCAATCTGATGGTGGTGGGGAGTATAATAGTAAAGCCTTTAAAGATTTCTTGGCTTCTAAGGGAATTCTCCATCAGATTTCATGTCCTTATACCCCTAATCAAAATGGGATTGCTGAGAGGAAGAATAGGCATATTATTGAAACCACAATTACATTACTTGCTACTGCTGCCTTGAATGACAAGTTTTGGTACTATGCTGCAGCTCATGCTGCCTTTCTCATAAATCGTATGCCTTGTCAGCTTCTTCAAATGACATCTCCATACTTCAAATTGTTTGGTCATAATCCTGAGCTTCAATCATTAAAAGTTTTTGGATCTGCAGGTGTTATTTGTTTTCATCCTGACACTCATAAAGTGGTTATTTCAAGACATGTGATTTATGATGAAACTCAGTTTCCAATGAAGAATTATTCAGTATCCTCAACCCAATTTTCTTCTGTCATGACACACTTGTCTCCTTCAGCTATAGCAGTTTCATTGCCTTATAGATATGTTACTCCCACAGAGTCTGATCACTCTCCTCTCCCATGTTCAAATGAGAATTCATCTTCTTCAGTATCTACATCTGAGGCTCAAGAGGTCTTAGGTTCTGCTCAGACTATGCAACCTATTTTGGATCCTGCTCAATTACAGCATGGTCATTCTATGCAAACTCGTCTTAAAAGTGGTATTAAGAAGAAGAAAGATTTTGGTGATTTTCAATGTTTTTCCACTTGTTTATCTACAATTACTGCTCTTGATGAGCCTCATACTTTTCGCGAAGCTAGTACAAAGTCTGAGTGGCAGCAGGCTATGACAGAGGAAATTCAAGGTACATGGGATTTAGTTCCTCCACCCTCTGATAAGAATATTGTTGGTTGTCGTTGGATTTACAAAATTAAAAGACATGCTGATGGACGTATTGCTCGATATAAAGCTCGTTTAGTTGCACAAGGTTTTAGTCAAGAACAAGGTATAGATTTTGACGAAACCTTTAGTCCTGTAGTTCGTCATACCACAGTTCGATTGATTTTAAGTTTGGCTGCAACTCATAAATGGTGTTTGCGCCAATTGGACGTTAAAAACGCATTTCTTCATGGTGACCTACAGGAGGAAGTGTACATGAAACAGCCTTTGGGATTCATTGATCATCATTATCCAGATTATGTATGTCGGCTACGAAAGTCTCTTTACGGTCTTAAACAGGCACCACGTGCTTGGAATGCCAAGTTCACTGGATATCTTCCAGCTTTCGGCTTTGTTTCTTCACACTCCGATCCTAGTCTCTTTGTGAAGCATGATGGTCCTAATGTGGTTATTCTTTTGTTGTATGTGGATGACATTATCATCACTAGGTCAAGTTCTACCCTAGTGCAATCTGTGATTGATGATTTGGGACAAGTGTTTGATATGAAAGATATTGGCCAGCTTACTTATTTCTTGGGCTTGGAGGTTTCTTATCAGTCCAATGGTGATTTGTTTGTCAACCAAGCTAAATATGCTGGTGATTTACTTAAGAGAGCTGGCATGGAAACGTGTAAACCATCAATTACACCTTGTAAACCTCATTGTCATGTTCTGACAACTGATGGTACATTACTTCCTGATCCTACAATGTTTCGCAGCTTGGTTGGAGCTCTTCAATACCTCACATTCACACGCCCAGATCTGGCATATGCTGTGAACACCGTATGCCAGTTTATGACTGCTTCTACTGATATTCATATGACTTTAGTTAAGCGTATACTGAGATATGTGCAAGGCACTTTATCTTATGGTTTGACATTTACATCTGGTTCTTCTGTTTTGGTTGGCTATAGTGACGCAGATTGGGCTGGGGATCCTAATACTCGTCGCTCTACTACAGGGTATGTTGTATTTCTTGGACAGAATCCTATCTCTTGGTCTTCTCGTAAGCAATCTTCTGTCTCTCGTAGTTCTACAGAGGCAGAGTATCGAGCTCTTGCCAACTGTGGATATCTCTTGGATCAGATATGTTCTTCAAGACTTACATATTGTTGTTCCTGA

>Pp06:g1.t1 transposable element 2

ATGGTCTGCAACTCGCATGGCTTCGAGCACTCCCACAAAACCCTACACTTCAACCTCGACCGCCACGTCATCATCGTCACCAACCCCATCACCACTGCATCCCATTTTGACTATGAGACTGCAGTTAATGACTACTTGGCAAACCGTCCTTCTTTTGGTTCCTCTGTTCAAAGGTCTCAAGTAATTCCTGAGTGCCAAATTTGCAACAAACGAGGCCATACTGCTCCAAACTGCTTCTATCGAGTTCCTGACAATTCTTCTGTGCCTAATCCTGTTGTGGAATGCCAGATTTGTGGAAAGAAAGGTCACACAGCCTTGAATTGCTACCATAGAGCTAACTTTTCCTATCAAGGCATTGCACCTCCTGCTCAGCTCCATGCAATGACAGCTCAAACCTCTCCTTCATTTTTTGCTGATGAGTTTTGGATCGCCGACAGTGGTCTGTGCATTGCTCATGTTGGCAGTGCTTCCATTCCTAGTATTTCTGGTTCTCTTCGCCTTAATCAAGTTTATCATGTGCCTCAGTTGGCTGCCAGCTTGTTATCCATTTACCAGTTGTGTAAGGATAATAAATGCTGGGTGATTTTTGATGACTCATATATCTATGTGCAGGACAAAGCAACGAAGGTTCTGCTGTACAAGGGTCGCAGTAACAAGGGTCTGTATCCAATTCCTCAAGCTCTGGGTGATTTGTCAATAAGGTCTAATACCAAGTCTTCACCAATTGTAGTTGAAGGGATTGCCTCTACTCCAACAGCTTTACTTGGGAAACCAGTATCTTCAGTTCTTTGGCATCAAAGATTTGGTCATGTGTCCAACGAAGTTCTTACTCAGATGTTGAAACAGTCTCAAATTTCTAGTGTTTCAGATTCCTCTCAGAGTCGATTACACTCTGATGTATGGGGTCCTTCCTCCTCTGTAGCATTTGGTGGATATAGATATTATGTGTCAATCATCGATGACTGTACTCGATTCCTCTGGATTTTTCCATTAATCAATAAATCTGAGGTTTTTCCTACATTTGTGAAATTTCATGCGTATATTACTAAGCACTTTCAAGCTTCTGTGCAATATTTTCAATCTGATGGTGGTGGGGAGTATAATAGTAAAGCATTTAAAGATTTCTTGGCTTTTAAGGGAATTCTCCATCAGATTTCATGTCCTTATACCCCTCAGCAAAATGGGATTGCTGAGAGGAAGAATAGGCATATTATTGAAACCACAATTACATTACTTGCTACTGCTGCCTTGAATGACAAGTTTTGGTACTATGCTGCAGCTCATGCTGCCTTTCTCATAAATCATATGCCTTGTCAGCTTCTTCAAATGACATCTTCATACTTCAAATTGTTTGGTCATAATCCTGAGCTTCAATCATTAAAAGTTTTTGGATCTGCAGTATATCCACTTCTTCGACCCTATAATTCTCACAAGTTAGAGCCACGATCAGCAGAGCATGTGTTCCTTGGATACTCTCTTGGTTATAAGGGTGTTATTTGTTTTCATCCTGACACTCATAAAGTGGTTATTTCAAGACATGTGATTTATGATGAAACTCAGTTTCCAATGAAGAATTATTCAGTATCCTCAACCCAATCTTCTTCTGTCATGACACACTTGTCTCCTTCAGCTATAGCAGTTTCATTGCCTTATAGATATGTTACTCCCATAGAGTCTGATCACTCTCCTCTCCCATGTTCAAATGAGAATTCATCTTCTTCAGTATCTACATCTGAGGCTCAAGAGGTCTTAGGTTCTGCTCAGACTATGCAACCTATTTTGGATCCTGCTCAATTACAGGTTTTGCTTCCTCTCTCTGTCACAGATGCCTCTAGTTCTTCTTCTCATATTTCCATTCCACAGCATGGTCATTCTATGCAAACTCGTCTTAAAAGTGGTATTAGCAAGAAGAAAGATTTTGGTGATTTTCAATGTTTTTCCACTTGTTTATCTACAATTACTGCTCTTGATGAGCCTCATACTTTTCGTGAAGCTAGTACAAAGTCTGAGTGGCAGCAGGCTATGACAGAGGAAATTCAAGCATTACAAACTCAAGGTACATGGGATTTAGTTCCTCCACCCTCTGATAAGAATATTGTTGGTTGTCGTTGGATTTACAAAATTAAAAGACATGCTGATGGACGTATTGCTCGATATAGAGCTCGTTTAGTTGCACAAGGTTTTAGTCAAAAACAAGGTATAGATTTTGACGAAACCTTTAGTCATGTAGTTCGTCATACCACAGTTCGATTGATTTTAAGTTTGGCTGCAACTCATAAATGGTGTTTGCGCCAATTGGACGTTAAAAACGCCTTTCTTCATGGTGACCTACAGGAGGAAGTGTACATGAAACAGCCTTTGGGATTCATTGATGATCATTATCCAGATTATGTATGTCGGCTACGAAAGTCTCTTTACGGTCTTAAACAGGCACCATGTGCTTGGAATGCCAAGTTCACTGGATATCTTCCAGCTTTGGGCTTTGTTTCTTCACACTCCGATCCTAGTCTCTTTGTGAAGCATGATGGTCCTAATGTGGTTATTCTTTTGCTGTATGTGGATGACATTATCATCACTGGGTCAAGTTCTACCCTAGTGCAATATGTGATTGATGATTTGGGACAAGTGTTTGATATGAAAGATATTGGCCAGCTTACTTATTTCTTGGGCTTGGAGGTTTCTTATCAGTCCAATGGTGATTTGTTTGTCAACCAAGCTAAATATGCTCGTGATTTACTTAAGAGAGCTGGCATGGAAACATGTAAACCATCAATTACACCTTGTAAACCTCATTGTCATGTTCTGACAACTGATGGTACATTACTTCCTGATCCTACAATGTTTCGCAGCTTGGTTGGAGCTCTTCAATACCTCACATTCACACGCCCAGATCTGGCATATGCTGTGAACACCGTATGCCAGTTTATGACTGCTCCCACTGATATTCATATGACTTTAGTTAAGCATATACTGAGATATGTGCAAGGCACTTTATCTTATGGTTTGACATTTACATATGGTTCTTCTGTTTTGGTTGGCTATAGTGACGTAGATTGGGCTGGGGATCCCAATACTCGTCGCTCTACTACAGGGTATGTTGTATTTCTTGGACAGAATCCTATCTCTTGGTCCTCTCGTAAGCAATCTTCTGTCTCTCGTAGTTCTACAGAGGTAGAGTATCGAGCTCTTGCCAACTATGCTGCTGATATCTCTTGGATCAGATATTTTCTTCAAGACTTACATATTGTTATTCCTGAAGCTCCAGTCTTACATAGTGACAATTTATCAGCATTGGCCTTAAGTAGTAATCCAGTTTTGCACTCTCGCATAAAGCATTTAGAACTTGATTTTCACTTTATCAGAGAACGGGTTCAGCGTCATGATTTGGTGGTTCGATATGTCAATACTGAAGACCAAGTTGCCGACATTTTCACAAAGGGCCTGCATAGTCCATTGTTTTTGAAACACTGTCACAATCTTAGTGTTGGTCCAGCCACCACTGCGATTGAGGGGGGATGA

>Pp08:g1.t1 transposable element 3

ATGGCTACCAACACCTCAAGTGATGCTCCTACCAATGATGCAAACCCACCATCACCCACAACCAACAATTCTCTCACACCCACCCATGCTGCTCCACCTGCAGTCTCCACTATTGTTACCATTAAACTCGATAGAACCAACTATCCACTTTGGTTGGCTCAAATATCTCTCATTCTTCGCAGCCGTGACCTTTTTGGCTATGTTGATGGCTCGGTGCCATGTCCACCCAAGCATTCCCCAACCACAAATACTTCTCAAATGAACTCTGCTTATCTCAATTGGGTGCAACAAGATCAGACCATTCTCAGTTGGATCAACAACTCATTGAGTCCGGCAGTATTGGCTACTGTCGCTCTTTCCCCCACTTCTGGCCAGTCTTGGCAATCGTTGGAGCGTCACTATGCGTCCACCTCACAAAATCGGATTTTACACTTGCGAAATGAGTTGCTGCGTACCACTAAGGGAGATATGCCCGTTTCTGATTTTTTGGACAAAATCAATTCCATTTCTGATAATTTGGCTCTTGCAGGAAATCCTCTCACAGATGCCTTGGAGGCTCTTCTACTGAGTGCTGAACGACGTTTGGGGGAGCAGTCACAGCCTGTTCCTAATCTGAACACAGGAGTTACTGCCTTTGCTGCTTCACGTGGCTGTGGCAATCGTGGCCGTGGTTTTTCTTCCAGTCGTGGTTTTTCATCCAACAAAAATTCTGCTGCTCCTAATGGGCGTGGTCAATTTCGGTCAAACTTTCCCAATCAACCACCTTTCAATTCTCCAGGGGAGTCTTCTTCTTCAGTCCCTGTTCGCTCTATTTGCCAGATTTGCAACAAGCCTGGCCATCAAGCTTTGGACTGCTTTAACAGGATGAATACTGCATATGAAGGCCGAATTCCAGCAGGCCGTCTCTCTGCTATGGCCACCCAGTCTAACACTGCAAATCGATATGCCTCAAATGCTTGGCTCGTTGACACAGGTGCCAATGCACATATCACACCAGATATTGGTAATCTCAATAATGCTCAGGAATATGTTGGTAATGAACAGGTTGGCGGTATAGTGTTTCATCATCCGAGTCACAGTTTCCTTTACAACTTGTACACTCTGATGTATGGTGTTCTCCAGTTAATTCAGTCAAAGTGTGATGAAGGTGGAGAATACACCAAAAAGACCTTTTCTCAATATTTGGCATCTCAAGGTATCCATCAAAGGTTCTCTTGTCCAAAACATCCAGAACAAAATGGCTTGGCCGAAAGAAAACACCGTCACATAGTCGAAACAGATCTAACAACAGGTAGGGTGTTTTTGTCCCGCCATGTGCTGTTTGATGAAGGTACCTTCCCATTTGCACATCTTGCACTCACATCTCTCAGGCAAGGTGTCCTCTCTAACTCAGGTACAGAGTCTACTCTCTCCTTTAACTTTCCCATTACTAAACCTGATCTTGTTTACCCTGAGCCCATTGCAGCACCTAATGATCTTGTGTCGAGCCCAAGCCCACAAATAACACACCCTATACCATCACAGCCGTCATCTTCATCTCCTTTAAGCCAAGACAGAGGTCGTGACACTTCAACATTTTCTGCTCCAGTCCAAGAATTAGTTGTGATGCGTGAATCCTCTCCATCTACCGTACCGTTAGAAGCTGCCACTTCATCTATTCCACCTCTCATCACCAACATACATGCTGATACTACCACCACTTCGACTGAAACTGCTATATCTACACAACCTGTTCGGCATCAGATGGTGACTCGATCTCAAATAGGGACTTTAAAACCCTCTTCTCGGTACGCTTTGCATCTCCAAGTTGACTCTACAAGTGTTGAGCCTTCTTGTTTCAGCAAAGCTATTAAACGCACAGAATGGAGGACTGCTATGGCCACAGAATTCAGTGCCTTGCAACGGTGTGGGACGTGGACTTTGGTTCCTTTTCAATATCACATGAACGTTCTTCCCAACAAGTGGGTGTTTAAGATTAAAAGGCACTCCGATGGATCTATTGAGCGTTATAAAGCGCGTTTGGTGGCCAATGGTTTCCATCAACAGGAAGGACTCGATTATTCAGAGACGTTTAGTCCTGTGGTTAAACACACCACAATTCGAATGGTTCTTGGGCTTGCTGTGTCTAATAAATGGCTTGTTCGTCAATTGGATGTTCAGAACGCATTTCTTCATGGTTTTTTGACAGAAGATGTATACATGAAGCAACCGGCTAGCTTTGTTGATTCGCAGTATCCCAATCACGTCTGCAAGTTGCAGAGGTCACTTTATGGTTTAAAACAAGTACCTCGAGCCTGGTTTAAACGGTTCAGTGATTTTCTACTACAACTTGGGTTTCAAGAATCCAGGTGTGACTATAGCTTATTTGTGTATAAACACAATGAGGTCTTTCTTATTCTACTTATTTATGTAGATGATATTTTGGTGACTGGAAATAATTCTTCTCAGGTTATGATGCTTATTCAGAGATTGGGAAAATTATTTTCTATGAAGGATTTGGGTCGTCTAAATTATTTCCTGGGTATTGAGGCTACTTATGAAGGTTCCGCGTTGCATTTGACTCAAAAGAAATATGCCTATGATCTTCTTACTCGCACTGGTTTTGCAGATTGCAAGCCTATATCTACACCATGTATTTCTGGTCAGAAACTGAGTCTCCACGGTGGGGAGCCTCTTGTAGATCCTTCTGAATATCGCCAAGTTGTGGGTGCCTTACAATACCTCACGATTACTCGGCCAGACTTATCTTATGCTGTGAACCAAGTGTGCTAG

>chr3:g1.t1 uncharacterized 2

ATGGAGGTGCTGCATAGAGAGAAAGGCTTGTTTCCTGCCGTTCCCTTATTTTTTCAGTATCCATGTGGTATCAACAAGGGTTGGTCAGATTGGGTTGATCGTGAACTAAGGGACCCGTCTACCTGTGACATCCTATGCTGGACGGGGGTGCTGGATGCCATCTTTCTCTCTAAGGCATGTGACATCCACATTGAAGCCAAGATGCTCCGTCATGTAGTTAGGCGATTGAAGGGCAAAAGGAAGCCTGAGATCTTACGGAAGGGTGTCCAGACTGGTCCTAGCACTTCTCTGCGGTTTAGCAACTGGATACAGTATTTCGGGGATGCGAACAGGAATGCATCGTGTCGTTTTGCCGAGCGCTTTAAAGGGATAGATGTGCCTCCACTTCCTCATTCACAAGGCAGACTGCTTATCAATTCCAGTGAGGGCTCTTATGTAGCGAAAAGACTCCATCTGCTAGAAGAATTTAAACATGTGCCCCTTTACGCTAATACTGATGGTCTAGTTGAGGCTCCTGTAATGATGGCACAAGGTCATCATTTGAGAAAGGATGCCCTTCGATTCCATGCTGCTTATTATGATAGACTGCCCCGCACAACAGTCTGTCATGCCCATTCCTTTTATTCTAAAGAAAAGGTCAACAGTCGATTCCATGCTGCTTATTTAGTGTCGAAAGAAAAGGTCATCTCTTTGAGTGAGAAGCAAAACTTGCCTTTCACTTCCAAGAGTGGCGAAATTGTTGGTGACTTTTCCAAGTTGAAACAGAAGATGGAGGCTCTCACGATGTCGGGAGGAGCACTGCACGTGGAAAGCGAAAGCGGGAGAAGATTTACGTCGCTGAGAAAAAGCAAGCTATCTAGGAGACAAAGAGGTTCATCCCTAAGATGGCAGCAGGTGGTCCCCCTAGAACCAAAGGGAATGCTCCCTTGGAGCCCTCACAGCATCGGGAACCGATGGCTTCTAGCAGTAGCAAGCATGTTGGCAAGGCGCAGGAAGCTTTGCCTTCTCATGGTGAGTACCCCTAAGAGTAAAAGCAAAGACAAGGAAACCCCGGTGATCTCGAAGCATCAAAGCATGCGCATTCATCAAGCAAGGTTCGTGGATACCAGTAAAGGCAAGGGTGAACGCTCAGGAGCCAAGGTTGTGGTGGCACGAACGAGGATCTTGATGAGGATCAATAATACAATCCTGATGAAGGCACATAAATGGGTCCTGACACTCGTTCAGAGTAGCTGGATGACTTGGATGTGGCTCCCAAATTTGCTGGAGACCGTTGCCCATACTGTTGAGAACATGGCAGACCGTTCAACAGAGGCTAGCCTTAGCCTTCCTGCTGCTATCACTGATTGCACGCAAGATGTCGGCAAGATTGGGGATGCTTCGCAAGGTGCAGAGCATACGCTTCTTGCCCTTCCTCCTCTTGAAACACTCGATGCAACTGCTAACTGGTTGTCAGGTCGGCAATGCTCCTACCGAGAGTGCTCCCGCAGGGCATTTATCAGAGAGAGGCTTGTCCTGGCAGGACTGGGAAAATTCCTTCACTGCGTTCAAGACCTTCTTTGTCACAGATCTTATGCTTCTTTGTTGGCGGAATGCAATCTGCGAGGCCATAACTTTGGGGTGCCGTATTTGGGGCAAGAGCCATTCATAACATGGAGTCGTCACTTGGCTCTTGGCCCTAATGAGGTAAAAGCTGCAGCTGACACCTTAAACCTCAAGCAGCGAGAACTGGAGGACCAACACAGGGAATTGCATGCCCTTCTTCTTGCTAAAGGTGTCTCTGTCGATGGTGCATAA

>Pp08:g1.t1 uncharacterized protein 1

ATGACAAAACAAGTGCCGCTGCAAGTGTGCTTCACTTTCACAAGGATTCTGAAAGTAATGGCGGCTCCTACTCCTACAATTCCAACTTCAAAAGCTATTGTTGTCGGTCCTCCAACACTGCTCACAAGAAGATTAATGGCTTCCAACTCCAACGGTGACATGGCCAAGAAGATCTCCATCTCCCCTATTTCCTCCTCCTTCTCTTCCTACGGTGATCCCTGCCATGATCTGTTTTTCCAGGTGACACGACCTGAAACACGCAGCGATGAGGAGACGACCCAGCAGCAGCAACAGCAGAACCAGGTGTCCCTTGACTACCTGAAGACACTGCCGCTGGCCTGGTCCCACAATCCCCTAACCACCCTCAAGCTCATCTTCAATCTCCATGCTATTCGTAGCAGCGGAAAATGTTATTCGGAAGGCTTCTACACGGCTGTGTTTTGGCTCCAACAGAAGCACCCCAAGACGCTATTATGCAACCTGCCGTCCATTGCTGATTCGTTCGGTGGTTTGTATGTCCTTATCGAGATTCTCTGCTGCCTTCTAGAACAAGACCAAGACGCTGCAGAGAGGCTCCACTCTGACCCGGACTATCAGTTGTTACACGACCGGGCAATGGATGTCTTCGTGGAGCGGTTGAAGTCTGATATTGACCAAATGAAGCAGCACAAGCTAGATTTGAAGCCATCAGATTATATAACTAACGGTGATGATGACGACGACGATGAAGATGATAAAGATGGTACTCTTGACGCTGACCCTTATGCTGATCTTTTTGTTAGCGAGGCTGCAGGGTGTTGCATTACCAAACAACCCCAGGACTCCTGCGCTGCCCGCACCATTTTTCTGTGTGAAAGCATTGCGAGGAGGCTTTGCCCACCCAAATCAAACCAACCAAATCAATCTTATGAATCCGAAGAGTGGGAGTGGCTTAGGAATGAGGTTTTGGCGCCCTTGAACAAGTACTGGAAGCGTCAAGGCATGTTTATTGGACGACAACGCTCTGAAGTTAAGATGTATTTGGAGGAGGTGAAAAAAGGAGGAAGAGGAGGCAATTTGAGTGGCCATGGCGGAATAATAAAGCCAGATGCTATGCTCCCAAATGAGATCATACGGTATGTAGTAGAAGATGGGGATGTCAGGGAAGGGGCTGAGCTTCAGTGGAAGGCAATGGTGGAGGATATGTACCTAAAGCAGCAGCAGCAGCAAAAGCAGGGGGAGGGTTTGGGAAAATTTAAAAACTGCTTGGCAGTGTGTCACATAAGCGATTACAATGGCCTAACGCGTTTGGCGGTGAGTTTGGGACTTTTGGTGTTTGAACTGAGTGAAGAGCCGGCATGGAAAGGAAAGGTGATCAGTTCTGGTCATTTGCTGGATCAGCTGATGCTGCATTCGATACAAGGGGATGATCTCAAGTGCGAGTTGATGATGAGTACATGCAACAGAAACTTTGTATCTTTTGCTGATAATTGGCAGATATGGGATTTTATTCTGGAAGTGGCTGCGAAAGAGAACTTGAAGGCAGATGAGATGGTTAAGAAGGTGTTTGTGTTCGCCGACTACTATGGATATGTTGGGGGTACATCCTGGAAGACTCTGTATGAGGCAAAACAGAGAGAGTTTAAGGAGAAAGGGTACGAGGATGATGCAGTGTCACACATTTTGCACTGGAATATTTCGTACCAGAACATGCCTCGTATAGAAGAACATCATCCAGGGGTGACGCTGTTGAGTGGCGTCTCTGACAATTTGGTCAAGTCCTTCTTGGACAACTATGGGGAAATTGGCCCGCACCATCTAATGGAAGCAGCCATTGCTGACAAAGCGTATCAAGCTCTCAGTGTGGTCGACTGA

>Pp02:g1.t1 uncharacterized protein 2

ATGGGTAGAGAGCGAGGTTATGTTGATGACGATATTGATGAAAGTAAGTTCAAGACTTCGGAAGCAATGAACCGCTACAAGAAAATCTTCAGTGTTCAAGCTGTGACGGTTGAGCGGGAGGTCAAATTGAGTGACTTCGAGGATCTTGGGCTTCCCAGAATCTTCAAATCAAGAGGTTGGCTTTTGGCAATGGGTCCTTCAGAGCCTGCTAATATCCAGATTGTCCAAGAATTCTACGCAAACATTCCTCCTTTCTCCGCCGAGCAGAAAACCCCACCTGGGTTGGGCCCTTTTGGTTCTTGTTTGTGGGATGAGACTCTGCATTTCTCTAGCGGTCGAGAATACCCTGACTCATTTGATGTGTATTTGCGAGGTAAGGTGTTGAATTTCTCTGTTTCTGGCATTGCCCAGTTGCTTAAACTTACTAGGCCAAACCCAAATGAAAAATCACCTGGTTTTCCTGGGCTTGTTGTTGATAATCTTGACTTGAAGGTGGTGAAATCTACTTTGGGTTGGAACAAGAAGGTCAGGGTTTTGCGTGAAAATCGACTGAGTGATTTGTACAAAGTGCTTAACAGCATAGTGAGATATAATATCGATCCTCCTTGTCACGTTATCCCTTCTTTGCTTAGCCCTGATAGAGCTCGCCTTCTTTATGCCATTGGGAACAATGTACCTATTGATCTGGCCACCTACATTTTTCGTGCCATCTGCCGTGCTGCATTCCCAACTTCCATGCCCGACTCCCTGCCTTTTACTTCCTTGATCACACGCTTCGCCATGGCTTCTCACGTGCCAGTTGAGCCTACAGATAAGCTTTATTCTCCTTGGTCGCCTTTGGACAACGTGGATGTTTTTGGCTATAGCTTTACTCCATCTTCTCCGGCAGTCAAGCAACCTCAAGCGGTGGTGAGCCACACGCCCAGTATGGCCATACTTACCAATGGCGTTGCTGAGAACAGGGGCGATGTGCACCCCGTTTCCACCCACTCGGTCATCCCACCTTCTTCGAAACAGCCATCTGCAGGTGGTAAAAATTTCCGGGGGTTTTCGTTGGTAGCATCATAG

>chr7:g1.t1 F-box family protein with DUF295

ATGATTCTAGGATCGTGTAGGATTCGACGGATTGCGAATCGGAGTCCCGGATACTCCGAAATCGCGAACCCTGGGGCTAGGGAACCTTCAGAGGAGCCCAGAGTGGCCATCAGAGACCGTGGACCCACGGGATCCCGGATCGGCCGATCTGGCAGCTTATCGCTTATTGAGGCTTCGGGCACCAGAGGTCCGGTCGACCCGCAGCCGCACCAGAGGTCATCTACTTGGTGTCCCGATACCTGCCAGGATTGCGGCTCGGCTGACTCTGTGTCCCCGAGACCTGCCAGGATTGCGGATCAGGCTAACTACGGTCCTCTGCATCCTGCCAGAGCGACTCGAGCTGACTTGGTGTCATCGAGGAATCTGCCGGCAGATCAGGCTGATCATAGTCCCCTGATTTCGCCAGTTTGTGGCTCGGGTGGACTGCGTGGCGCCCGAGACCTGCCAGGGGAATTGACGGATATGACAAGGGCCGTAGAAGTACGCGGGATCCACCACCGGGCCATTTATAGCTTCCGCGCCTCAAGATCAG

>Pp05:g1.t1 hypothetical protein 1

GTTGGCTTGGCAGGTTTCCTTTGAGAAGGATGCAGATAGGTCCTTCATACGCTGTGGAATTGCCGCGCATGACGTGCCTGTGCAGGCCCTTCATGACGACCCACCGCATTCCTCACATGACATGCATGTGCATGCCCTTCATGACGACCTACCGCATTCCTCGCATGACATGCATATGTATGCCCTTCATGACGACACACTACAATCCCCGGATGATGTGCCTGTGCATGCCCTTCATGACGACCCAATGCTGTCCTTGCATGACGAAGACGATATAAAGGATAAGCAATACTTATTTGACGGGGTGAAGCTCACTAAAGCAGAAGATCGCGAACTACAGTCAGATGAGGTTGAGGATACCAATTTTGAAGCAGCAACACAACCAATCTTCAATTCATTGCCTTCCATAGCTTCTTACTTAGAATTTGAACACAACTCGTCATCACAGTCGCCTCAACTTCAAACCCCAATTGATTGGCCTCTTGATGGCAAGCTCACCCTCAACTGGGTCCAAAGCCTCATGTCCGTCTTCGACTGGGCATCTAGAAATCTTGAGCCGACCCAATTGCCAAACGTGTTTCTTGTTGAGGTTTTTGATAGCCTGGCCCTATTTAACTCCAAGATCCTCCACAAAGAGGCCAATTGCGTCACCATTAATAACTTAGCCTTTGAATCCACGGTCGTTGTCGTCGGAGACCTTCATGGGCAGTTGCATGACCTTTTTTTCCTTCTCCATGATGTTGGCTTTTCCTCAGAAAATCGATTCTTCGTCTTCAATGGCGATTATGTTGACAGAGGTGCTTGGGGTCTTGAAAGTTTCTTAATTTTGTTAGCTTGGAAAGTGCTCATGCCAAAGAGGGTGTATCTATTGCGAGGAAACCACGAATCAAAGTATTGCACTTCTGTTTATGGTTTTGAGAAGGAAGTGCTGACAAAGTACAATGATAGAGGCAAACACGTGTATTGTAAATGTCTTGGGTGCTTTGAAGGTCCCAATGTGATTCCTGGTGATGTGCTGTGGTCGGATCCCTCAATATCTCCCGGCCTGTCTCCAAATATAGAGAGAGGCATTGGGCTGCTTTGGGGTCCTGATTGCACCGAGGATTTTCTCAAGAATTGTCAACTCAAATTGATTATTAGATCCCACGAAGGTCCTGATGCACGAGAAAATAGGCCAGGTCTTGGTGGAATGGATGAAGGGTACACCATCGATCACATTGTGGAGTCAGGAAAGCTCATCACTTTGTTTAGTGCTCCAGATTACCCACAATTTCAGAGCACAGAGGAGAGGTACAAAAATAAAGGGGCTTACATTATCTTGGAACCCCCTAATTTTGATGATTATGTATTTCATAGTTTCGAAGCAATTACTTCAAGGCCAAAGGCGGATCCCTTTTACAATTTTGAAGAAATGATTGATTCTGATGAAGAGTTGGACTTGGCATCAATGGTACAATCATAA

>chr6:g2.t1 hypothetical protein 1

ATGCCTTCGGCACCCCCGTCATTTTTCGGCGAGAGCGATGCCTTCGGCACCCTGCCATCATTCGGCGAGCGTGATGCCTTCGGCACCCTGCCATCATTCAATGAGAGCGTGATGCCTTCGGCACCCCCGTCATCATTCGATGCCTTTGGCACTCCGTCATCATTAGACGAGAGCGATGCCTTCGGCACCCCGTCATCCTTCGATGAGAGCGATGCCTTCGGCACTCTGTCATCATTAGACGAGAGCGATGCCTTCGGCACCCCGTCATCCTTCGATGAGAGCGATGCCTTTGGCACCCAATCATCATTAGATGAGAGCGATGCCTTCGGCACCCCGTTATCATTCGACGAGAGCGATGCCTTCGGCACCCGGCCGTCATTCGATGAGAGCGATGCCTTCGGCACCCCGTCATCATTTAGCACGAGCCATGCCTTCGGCACCTCGTCATCATCGACGAGAGCAATGCCTTCGGCACCCCGTCATCATTCGGCGAGAGCGATGCCTTCAGCACTCCGTTATCATTGGGCGAGCATGATGCCTTCGGCACCCCGTCATCATTCGACGAGAACGATGCCTTCGACACCCCCGCCATCATTCGGTGAGCGTGATGACTTCGGCACCCCCGTCATCATTCGACGAGAGCGATGCCTTCGGCACCCCGTCATCATTCGACTAGGGGCGATGCCTTCGGCACCCCCGTCATCATTCGGCGAGAGCGATGCCTTCGGCACCCCCGTCATCATTCGACGAGCGATGCCTTCGTCACCCTACCATCATTCGACGAGAGCGATGCCTTCGGTACTCCACCATCATTCGGCGAAAGTGCCTTCGGCACCCTGTCATCATTCGGCGCGAGCGATGCCTTCGGCACCCCCGTCATCATTAGGCGAAAGTGCCTTCGTCACCCCACCATCATTCGACGAGAGCGATGTCTTCGGCACTCCACCATCATTCAGCGAAAGTGCCTTCAGCACCCTGCCATCATTCGGTGAGAGCGATGCCTTCGGCACCCCCGTCATCATTCGACGAGCGATGCCTTCGTCACCCTACCATCATTCGACGAGAGCAATGCCTTCAGCACTCCACCATCATTCGGCGAAAGTGCCTTCGGCACCCTGCCATCATTCGGCGAGAGCGATGCCTTCGGCACCCCCATCATCATTCGGCGAAAGTGCCTTCGGCACCCGGTCATCATTCGGCGAGAGCGATGCCTTCGGCACTCCACCATCATTCGGTGAAAGTGCCTTCGGCACCCTGCCATCATTCGGCGAGAGCGATGCCTTCGGCACCCCCCATCCACACCACACTACTTGGAAGAAGACTAAGGTGTTGCTCAGTCAGTGCATTGGAAATTACAAGTTCTCAACCAAGGAGTACCTCCTTACTTAA

>chr4:g1.t1 hypothetical protein 2

GGGAGAGGAGGATTGAAGAGAAGTTGTGTCTGAGTGAATTAGCTAAAGTTTATATGGATGAATTTATTGTTGCCATTGTCTCTACTGCCGTGCTCATGGGGTTTCGGTGTTCTGCCTTACCTATTGCCGTGCTCACAGGGTTTCTGTGTTCTGCCTTACCAATTGTTGTGCTCACACAGTTTCTGTGTTGTGCCTTACCTACTGCCGTGCTCACAGGGTTTCTGTGTTCTGCCTTATCTACAGCTGTGCTCACAGGATTTCTGTGTTCTGATGGCTCCGCTCCTACAGATGCAGACTTTGACCCTGAACCCGATCCAGAATCAGGATTCTCTGTTGACGACCCCGACCTCGACCCCGACTATGACTACGACGACCCTGGCCCCGACTCCGACCCCGACTATGACGACGACGACCTCGACCTTGACTCCGAAGACGACCTCGACCCCGACCCCGACTCCGAAGACGACCCCGACCCCGACCCCGACTCCGAAGACGACCCCGACCCCGACTATGACGACGACGACCCCGACCTCGACTCCAAAGACGACCCCGACCCCGACCCTGACTCAGAAGACGACCCCGACCCCCACTCCGATGATGACCCCAACCCCAAATTTGTCTCTTATTCAGACCCTGATCCCGATTTCGACTTTGACTCCGACCTCGACTCAGGCTCAGATTCTGATCCCAATTCCTACTCAACCT

>chr5:g1.t1 hypothetical protein 3

GCGGCTCGGGTGGTCTGCGTGGCGTCCGAGACCTGCCAGGGAATTGACGGATAAGACAGGGGTACAATTAGGTGGTGAAATTTTTTGGGATTGGTCAAAATACAGGGGAGACTCTGCCGAAATTTCGGCAGAAGTCGAAGAGAAATTTGAAAAGAATTTGAGACAGGAAGGCTTCATTTCGTGGCTGTTTCCGGCGACAGCAGCGGCGGATCAAGGTGGCGGTGGGTTGGAACGGAAAGAAGGAAGTGTGGTGGTTCCAATAGAACCGGTGCCGTTCAGTTTGGAGGTGTGTGGTGACGATGGTGGAGGCGTGAAGGTGCTGTCGTGA

>chr1:g2.t1 receptor kinase 3_2

ATGGCTTCCTCTTCTTCTTGCCCCAATTATTTCAATTTAAATGATGCTCCCACAACAACCAGTGACGCCAAAGTTTGGCGTCCATCATTTATATCCCAAAATCGTCATCTCACAGTTAATGATTCTGCGATGATGAATGACGCTACTGCTGTCATAGTAGCTAGGAATTTCATTACTCCAATGGATGAAATGCTGTTAACAGGGAGGTCTGAGGAAGAGGCTATTAATGACTTAATGGCTTCTAGTATTCAGAGTGCTGCTTCTGTTTCTAACATGACTGATCGTTTGCGTGCTAGAGCAAACGAGGTTCAGAAGCTAACAATTGAAAATTCGTCTATTCAAAGAATGCTTCATGAGTCTCAACAGGAGGTTGAGAAACTTAATGGAGAGAATAATGCCTTGTTGAAACTGGTGAGTTCATACTCCGTTGATACACTGAGAAGGCTAGACATGAAAGTAGCTCCGTGA

>chr2:g1.t1 receptor kinase 3_3

CTAGCATTCAGAGTGCTGCTTCTGTTTCTAACTTGGCTGATCGTTTGCGTGTTAGAGCAAACGAGGTTCAGAAGCTGACAACTGAAAATTATTCTCTTCAAAGAATGCTTCATGAGTCTCAAAAAGAGGTTGAGAAACTCAAAGGAGAGAATAATTCCTTGTTGAAACTGGTGAGTTCGTACTCTGCTGATACACTGAGAAAGCTAGACATGCTGCAGGTCTCAAATGAAAGAATTTTGGGAGACCATGAGAGGCTCATGGCTAGGCTTAAGAGGCGCCGTCCTCTTCCTTCAGAGGCTTCCAGAACATAA

>chr1:g1.t1 receptor kinase 3

ATGGCTTCCTCTTCTTCCTGCCCAAATTATTTCAATTTAAATGATGCTCCCACAACAACCAGTGACGCCAAAGTTTGGCGTCCATCCTTTGTATCCCAAAATCGTCATCTCACAGTTAATGATTCTGTGATGATGAATGATGCTACTGCTGTCATAGTAGCTAGGAATTTCATTACTCCAATAGATGAAATGCTGTTGACAGGGAGGTCTGAGGAAGAGGCTATTGATGACTCAATGGCTTCTAGCTTTCAGAGTGCTGCTTCTATTTCTAACATGGCTGATCGTTTGCATGCTAGAGCAAACGAGGTTCAGAAGCTAACAACTGAAAATTCGTCTCTTCAAAGAATGCTTCATGAGTCTCAACAGGAGGTTGAGAAACTTAAAGGAGAGAATAATGCCTTATTGAAACTGGTGAGTTCGTACTCCGTTGATACACTGAGAAGGCTAGACATGTTGCAGGTCTCAAATGAAAGAATTTTGGGAGACCACGAGAGGCTCATGGCTAAGCTTAAGAGGCGTCGTCCTCTTCCTTCAGAGGCTTCCAGAACATAA

>chr5:g2.t1 Retrovirus-related Polpolyprotein from transposon TNT1-94

ATGGCTGCAGACGAAGGGAAGATGAAAATTGAAAAGTTCGATGGTGCGGACTTCGGCTTTTGGAAGATGCAGATCGAAGATTATCTGTATCAGAAAAAGCTTTATCAACCTCTTTCAGAAAATAAGCCAGAGAGTATGAATGATAAAGACTGGACTCTTCTTGACAGACAAGCCCTCGGAGTTATCCGATTGACGCTATCCCGCAATGTTGCTTTCAACATAGCAAAGGAAAAGACCACGGCAGGTCTCATGGCGGCTCTTTCCAGTATGTATGAGAAACCATCAGCCTCTAACAAAGTTCACTTGATGAGGCGGTTATTCAATTTACGGATGACGGAAGGCGCATCGGTAGCTCAACATCTCAATGAACTCAATACAGTCACAACCCAGTTGAGTTCAGTTGGAATTGAATTTGATAAAGAAGTACGAGCATTGATACTTTTGTCTTCTCTACCAGAAAGTTGGAATGCTACTGTCACGGCTGTGAGTAGCTCGTCGGGAAGCAAGAAGTTGACATTTGATGATGTTCGTGATCTGGTTCTCAGTGAAGAGATCCGACGGAGAGAGTCGGGTGAATCGTCAACCTCTTCTGTTTTGCATACAGAGTCAAGAGGAAGAAATTCAACCAGAGGGAATGGACGTGGCAGATCGAACTACCGAGGTAGATCAAAGGACAGGAGGTCCAAATCCAGGAATCCTAATAATTCCCATAGCTCGAAGACCGTCGAGTGTTGGAACTGCGGGAAGATCGGGCACTATAAGAATCAATGCAAGAGTGCATCGAAGGACCATGAGGCGAAGGCAGAGGCAAATGTTACTTCCACCTCAGGAGGAGATGATGCGTTGATATGCTTTTTGGAGAGCAATGAAGAGTCTTGGGTGTTAGACTCTGGAGCATCATTCCATGCTACTTCGCAGAAACAATTCTTCGAGAGGTATGTCCCCGGAAACCTTGGAAAGGTATACCTTGGTAATGATCAACCTTGTGCTATTATTGGTAAAGGTGTAGTGAAGATTAAGTTGAACGGGTCTGTTTGGGAGCTAAAGGATGTCAGGCATATTCCCGACCTGAGAAAGAACTTAATCTCAGTAGGGCAGTTGGCTAGCGAAGGCTACACTACGATCTTTCATGGTGATGATTGGAAGATTTCAAAGGGCGCAATGATGGTTGCTCGAGGCAAAAAGAATGGTACTCTTTTCATGACAGCAGGGGGGCGCTGTTCAATTGCAATTGCAGCAGGAAATGAAAATCCCAATATGTGGCACCAGAGACTTGGCCACATGAGTGAGAAGGGGATGAAAATTATGCACTCGAAGGGGAAACTTCCAGGTCTAGAGTCGGTTGGGATAGACATGTGCGAAGATTGCATATTTGGAAAACAGAAGAGGGTCAGCTTTCAGACAAGTGGCAGAACCCCAAATAAGGAAAGGCTAGAGCTCGTTCACTCTGATGTTTGGGGACCAACGACCATTTCATCCATTAGTGGGAAACACTACTTCGTGACTTTCATCGATGATCACTCTCGGAAGGTATGGGTTTACTTTCTAAAGCATAAGTCTGAAGTGTTTGAGGTTTTCAAGAGATGGAAAGCTATGGTTGAAAATGAGACAGGTCTGAAGATTAAAAGGCTCAGAACCGACAATGGTGGTGAATATGAAGACACCAGATTCAAGAAGTTCTGCTATGAGCAAGGAATCAGAATGGAGAGAACCGTACCAGGTACGCCTCAACATAATGGTGTAGCTGAGCGTATGAACCGAACGTTGACAGAAAGAGCCAGAAGCATTCGTATACAGTCAGGTCTACCGAAGCAGTTCTGGGCAGAAGCAGTCAACACAGCAGCTTACTTGATCAACAGAGGCCCATCGGTTCCATTGGAGCATAGAATACCAGAGGAGGTATGGAGCGGAAAAGAGATAAAACTATCACATTTAAAAGTTTTCGGTTGTGTAGCATATGTGCATATTAGTGATCAAGGCAGGAATAAGCTTGATCCCAAATCTAAGAAATGCACCTTCATCGGCTATGGCGAGGATGAATTTGGGTACCGCATTTGGGATAACGAAAACAAGAAGGTGATCCGCAGCAGAGATGTGATTTTTAATGAAAGGGTGATGTACAAGGACATACATAAAAACGACGCCAGCAACACAGAGCAGAGTGTGCCAATATTTGTAGATACAGATGATGTCCCAGAAAGTCTCGTGACGGAGCCGATGGTAGCAAGTCCTCAGCTAGAGGAACTCGTTGGACAGAGCAGTGCGCAGCAGTCCGACACACTGCAGCCTCCTACTCCAGCTGCTGTATTGAGAAGGTCTTCTCGGCCACATGTGCCTAATAGGAGATACATGAACTATTTGTTGCTGACTGATGGAGGTGAGCCTGAATGCTATGATGTAGCTTGTCAGACTGGAGATGCTAGCAAGTGGGAGCTTGCCATGAACGACGAGATGAGGTCTCTGATCTCCAACCAGACATGGGAACTAGCTGAGTTACCCGTGGGGAAGAAGGCACTTCACAACAAATGGGTGTACCGAGTGAAAGAAGAACATGATGGTTCTAAGAGATACAAAGCCCGACTAGTTGTCAAAGGATTCCAGCAGAAGGAAGGAGTTGACTATACCGACATTTTTGCTCCCGTTGTGAAGCTTAATACTATCAGATCAGTGTTGAGTATTGTTGCCATTGAAGATCTTTATCTTGAACAGTTAGACGTGAAGACCGCATTTCTTCACGGAGACTTGGATGAGGAGATATACATGCACCAGCCAGAAGGTTTCTCAGAAAGAGGGAATAAGAACATGGTGTGCAGACTTAAAAAGAGTTTGTATGGCCTGAAACAAGCTCCAAGACAGTGGTACAAAAAGTTTGACAGTTTCATGCACAAGGAAGGTTTCCAGAAGTGTAACGCCGACCACTGTTGCTACTTTAAAAGATATAGGTCCAGTTATATCATTTTACTACTTTATGTCGATGATATGTTAGTAGCAGGTTCAAATATGGATGATATCAGAAGGTTGAAGCAGCAATTGTCAAAGAAGTTTGACATGAAGGACTTGGGTCCAGCAAAGAAGATTCTTGGAATGCAAATCACAAGAGATAAGCATAGAGGGATTTTGCAGTTATCTCAGACAGAGTACATCAACCGTGTTTTGCAGAGATTTAACATGGGTGACGCCAAGCCAGTCAGCACACCCTTGGCAAGTCACTTTCACTTATCCAAGGATCAGTCCCCTCAGACGGAGGAAGAGAGAGATCTCATGGCTAAGGTTCCTTACGCCTCAGCTATTGGGAGTTTGATGTACGCAATGGTCTGTACTAGACCAGACATTGGCCATGCAGTAGGAGTTGTTAGCAGGTTTATGTCAAATCCAGGGAAAGCTCATTGGGAAGCAGTAAAGTGGATTTTAAGATATCTACAAGGCACCACAGAGAAATGTTTGTACTTTGGTAAGGGTGAGTTAAAAGTACAAGGCTATGTAGATGCAGACTTTGGAGGTGAAGTTGATCACAGAAGAAGCACCACCGGTTATATATTCACTGTTAGAAATACAACTGTTAGTTGGATGTCACAGTTACAGAAGATTGTCACTCTATCCACTACAGAGGCTGAGTACGTAGCAGTGACTGAAGCCAGTAAAGAAATGATATGGCTTCAAGGTTTGTTAACAGAGTTGGGATTCAAACAGGAGAAGAATGTTTTGCATAGTGACAGTCAGAGTGCAATACACTTGGCAAAGAATTCAGCATTTCATTCAAGAACCAAGCATATTGGACTTCGTTCTCACTTTATCAGATCTTTGTTGGAAGATGAGGTGTTAATACTGGAGAAGATCCAAGGAAGCAAGAATCCAGCGGATATGTTGACAAAGACGGTGGCTATCGACAAACTGAAGTTGTGTTCAACTTCAGTTGGCCTACAAGAGTAA

>Pp07:g1.t1 serine/threonine-protein phosphatase 7

GACAACCCATCACCACCATCGACTGAGCTCGAAATCCCAATTGGTTGGCCTCCCGATGGCAAGCTCAGCCTCAACTGGATACGAAACCTCATGTCCGTTTTCGATTGGGCTTCTAGAAATATCGAGCCCACTCAATTGCCAGACGTATTTCCCGTCGAGGTTTTTGATAGCTTGGTTCTCTGTGCTTCCAAGATCCTCCACAAAGAGCCCAATTGTGTCGCCATTGACAACTTAGGCTCTGAATCCACAGTCGTCGTCGTAGGAGACCTTCACGGTCAGTTGCACGACCTTGTTTTCCTCCTCCACGATGCTGGGTTTCCGTCAGAAAATCGATTCTTTGTCTTCAATGGAGATTACGTTGACAGAGGTGCTTGGGGTCTCGAAAGTTTCTTAATTTTATTGGCTTGGAAAGTGTTCTTTCCAAGAAATGTTTATCTCTTGAGAGGAAATCACGAATCCAAGTATTGCACTTCTGTTTATGGGTTTGAGAAGGAAGTGCTTACAAAATATAGTGATAGAGGCAAGCATGTGTACCGGAAATGTCTTGGGTGCTTTGAAGGTCTTCCTTTGGCTTCCATCGTTGGGAAACATGTGTACACTGCACATGGAGGTATATTTCGCCATATCCCTGCGACCCCCAAGAGATCAAAGGGAAAGAAGAATCGTAAGATAGCTTTCAGTCCTGAACCAAGTTCATTATCTATTGGCTCTTTTGAAGAACTAAACAAGGCGCGAAGATCAGTTCTTGATCCTCCATGGGAAGGTTCCAACTTGATTCCCGGGGACGTGCTCTGGTCGGATCCCTCCATGACTCCCGGCCTTTCTCCAAATAAAGAGAGAGGCATTGGTCTACTTTGGGGTCCTGACTGCACTGATAACTTTCTGAAGAAATTTCAACTGAAGGTAATATCAAGATTTGGAATTTACATTTCTTATCATTGTTAA

>chr5:g1.t1 toll-interleukin-resistance domain family protein

GCAGTAGGAGTTGTTAGCAGGTTTATGTCAAATCCAGGGAAAGCTCATTGGGAAGCAGTAAAGTGGATTTTAAGATATCTACAAGGCACCACAGAGAAATGTTTGTACTTTGGTAAGGGTGAGTTAAAAGTACAAGGCTATGTAGACGCAGACTTTGGAGGTGAAGTCGATCACAGAAGAAGCACCACCGGTTATATATTCACTGTTGGAAATACAACTGTTAGTTGGATGTCACAGTTACAGAAGATTGTCACTCTATCCACTACAGAGGCTGAGTACGTAGCAGTGACTGAAACCAGTAAAGAAATGATATGGCTTCAAGGTTTGTTAACAGAGTTGGGATTCAAACAGGAGAAGAATGTTTTGCATAGTGACAGTCAGAGTGCAATACACTTGGCAAAGAATTCAGCATTTCATTCAAGAACCAAGCATATTGGACTTCGTTATCACTTTATCAGATCTTTGTTGGAAGATGAGGTGTTAATACTGGAGAAGATCCAAGGAAGCAAGAATCCAGCGGATATGTTGACAAAGACGGTGGCTATCGACAAACTGAAGTTGTGTTCAACTTCAGTTGGCCTACAAGAGTAA

>chr6:g3.t1 transcription factor bHLH110-like

ATGGATACCGGACGGCATGTCTCATTGTGCCATGCTCTCACATCTTGGGTCGGGGATTACCGCACACAGTTTTCTGATTGGATGCCATTATCCGAGACTTTTTGGCTTGTATTACTGTTCGGCGGGGCTACTTTGACCCTTCCCCAAGATGGTGGGCGTGGATGTTTTGCCGACGGCATGTTGCCGTTAACACCTTTCGAGTCGACGTCAGATTGCTTTGGCCCATACGTACGAGCATCGCCGATGAGGAGACTAGCTAGGCTTAGATGTCTTGATCAATCTTTTGGCATGCCCCCCTCAGTAAAGTATGCAATGTGGGCTCCCTTCAATAATGCTTTCCAAGGAATATGCCGAACGGTGATGCCGATTGTAAGGGTGCCGAAGGGCACTGAAGAAAGGGAGAACGACAGAGCCTTTTGCGTCGTTTCCCACAGACGGCGCCAAACTGTTGATGCTCAAAAATGGTTCAGCACTTTGAGCTCAACTTAG

>Pp08:g1.t1 uncharacterized Protein 1

GTATGTGTGTGTTCATTTCCGATTGCAATTTCAATCAGAGGAAGGGTTAGGGATGACAAAACAAGTGCCGCTGCAAGTGTGCTTCACTTTCACAAGGATTCTGAAAGTAATGGCGGCTCCTACTCCTACAATTCCAACTTCAAAAGCTATTGTTGTCGGTCCTCCAACACTGCTCACAAGAAGATTAATGGCTTCCAACTCCAACGGTGACATGGCCAAGAAGATCTCCATCTCCCCTATTTCCTCCTCCTTCTCTTCCTACGGTGATCCCTGCCATGATCTGTTTTTCCAGGTGACACGACCTGAAACACGCAGCGATGAGGAGACGACCCAGCAGCAGCAACAGCAGAACCAGGTGTCCCTTGACTACCTGAAGACACTGCCGCTGGCCTGGTCCCACAATCCCCTAACCACCCTCAAGCTCATCTTCAATCTCCATGCTATTCGTAGCAGCGGAAAATGTTATTCGGAAGGCTTCTACACGGCTGTGTTTTGGCTCCAACAGAAGCACCCCAAGACGCTATTATGCAACCTGCCGTCCATTGCTGATTCGTTCGGTGGTTTGTATGTCCTTATCGAGATTCTCTGCTGCCTTCTAGAACAAGACCAAGACGCTGCAGAGAGGCTCCACTCTGACCCGGACTATCAGTTGTTACACGACCGGGCAATGGATGTCTTCGTGGAGCGGTTGAAGTCTGATATTGACCAAATGAAGCAGCACAAGCTAGATTTGAAGCCATCAGATTATATAACTAACGGTGATGATGACGACGACGATGAAGATGATAAAGATGGTACTCTTGACGCTGACCCTTATGCTGATCTTTTTGTTAGCGAGGCTGCAGGGTGTTGCATTACCAAACAACCCCAGGACTCCTGCGCTGCCCGCACCATTTTTCTGTGTGAAAGCATTGCGAGGAGGCTTTGCCCACCCAAATCAAACCAACCAAATCAATCTTATGAATCCGAAGAGTGGGAGTGGCTTAGGAATGAGGTTTTGGCGCCCTTGAACAAGTACTGGAAGCGTCAAGGCATGTTTATTGGACGACAACGCTCTGAAGTTAAGATGTATTTGGAGGAGGTGAAAAAAGGAGGAAGAGGAGGCAATTTGAGTGGCCATGGCGGAATAATAAAGCCAGATGCTATGCTCCCAAATGAGATCATACGGTATGTAGTAGAAGATGGGGATGTCAGGGAAGGGGCTGAGCTTCAGTGGAAGGCAATGGTGGAGGATATGTACCTAAAGCAGCAGCAGCAGCAAAAGCAGGGGGAGGGTTTGGGAAAATTTAAAAACTGCTTGGCAGTGTGTCACATAAGCGATTACAATGGCCTAACGCGTTTGGCGGTGAGTTTGGGACTTTTGGTGTTTGAACTGAGTGAAGAGCCGGCATGGAAAGGAAAGGTGATCAGTTCTGGTCATTTGCTGGATCAGCTGATGCTGCATTCGATACAAGGGGATGATCTCAAGTGCGAGTTGATGATGAGTACATGCAACAGAAACTTTGTATCTTTTGCTGATAATTGGCAGATATGGGATTTTATTCTGGAAGTGGCTGCGAAAGAGAACTTGAAGGCAGATGAGATGGTTAAGAAGGTGTTTGTGTTCGCCGACTACTATGGATATGTTGGGGGTACATCCTGGAAGACTCTGTATGAGGCAAAACAGAGAGAGTTTAAGGAGAAAGGGTACGAGGATGATGCAGTGTCACACATTTTGCACTGGAATATTTCGTACCAGAACATGCCTCGTATAGAAGAACATCATCCAGGGGTGACGCTGTTGAGTGGCGTCTCTGACAATTTGGTCAAGTCCTTCTTGGACAACTATGGGG

>chr2:g1.t1 uncharacterized protein 1

ATGGGGCCTGTCCCCACGCCGGATAACCTACGCCACGCGGGACCTCAACATCATAACACAATATCTCCCCATACGCCGGTACAACCAACGCCACGTATGGGGCCTGTCGACACGCCGGATAACCTACGCCACGTGCGACCTCAACACAATATCACAATATCTCCCCATACGCCGGTACAACCAACGCCACGTATGGGGCCTGTCTCAACGCCGGATAACCTACGCCACGTGAGACCTGACTTTCACTTGGTGGTGCTTGTAGTGAAACCAAGATCCAGGGAG
